# Raptor genomes reveal evolutionary signatures of predatory and nocturnal lifestyles

**DOI:** 10.1186/s13059-019-1793-1

**Published:** 2019-08-29

**Authors:** Yun Sung Cho, Je Hoon Jun, Jung A Kim, Hak-Min Kim, Oksung Chung, Seung-Gu Kang, Jin-Young Park, Hwa-Jung Kim, Sunghyun Kim, Hee-Jong Kim, Jin-ho Jang, Ki-Jeong Na, Jeongho Kim, Seung Gu Park, Hwang-Yeol Lee, Andrea Manica, David P. Mindell, Jérôme Fuchs, Jeremy S. Edwards, Jessica A. Weber, Christopher C. Witt, Joo-Hong Yeo, Soonok Kim, Jong Bhak

**Affiliations:** 1 Clinomics Inc, Ulsan, Republic of Korea; 2 Biological and Genetic Resources Assessment Division, National Institute of Biological Resources, Incheon, Republic of Korea; 3 Korean Genomics Industrialization Center (KOGIC), Ulsan National Institute of Science and Technology (UNIST), Ulsan, Republic of Korea; 4 Department of Biomedical Engineering, School of Life Sciences, Ulsan National Institute of Science and Technology (UNIST), Ulsan, Republic of Korea; 5 Animal Resources Division, National Institute of Biological Resources, Incheon, Republic of Korea; 6 Strategic Planning Division, National Institute of Biological Resources, Incheon, Republic of Korea; 7 Chungnam Wild Animal Rescue Center, Kongju National University, Yesan, Republic of Korea; 8 College of veterinary medicine, Chungbuk National University, Cheongju, Republic of Korea; 9 Medical care team, Cheongju Zoo, Cheongju, Republic of Korea; 10 Department of Zoology, University of Cambridge, Cambridge, UK; 11 Museum of Vertebrate Zoology, University of California, Berkeley, CA USA; 12 Institut Systématique Evolution Biodiversité (ISYEB), Muséum national d’Histoire naturelle, CNRS, Sorbonne Université, EPHE, Paris, France; 13 Chemistry and Chemical Biology, UNM Comprehensive Cancer Center, University of New Mexico, Albuquerque, NM USA; 14 Museum of Southwestern Biology and Department of Biology, University of New Mexico, Albuquerque, NM USA

**Keywords:** Raptor, De novo assembly, Comparative genomics, Evolutionary adaptation, Predatory lifestyle, Nocturnality

## Abstract

**Background:**

Birds of prey (raptors) are dominant apex predators in terrestrial communities, with hawks (Accipitriformes) and falcons (Falconiformes) hunting by day and owls (Strigiformes) hunting by night.

**Results:**

Here, we report new genomes and transcriptomes for 20 species of birds, including 16 species of birds of prey, and high-quality reference genomes for the Eurasian eagle-owl (*Bubo bubo*), oriental scops owl (*Otus sunia*), eastern buzzard (*Buteo japonicus*), and common kestrel (*Falco tinnunculus*). Our extensive genomic analysis and comparisons with non-raptor genomes identify common molecular signatures that underpin anatomical structure and sensory, muscle, circulatory, and respiratory systems related to a predatory lifestyle. Compared with diurnal birds, owls exhibit striking adaptations to the nocturnal environment, including functional trade-offs in the sensory systems, such as loss of color vision genes and selection for enhancement of nocturnal vision and other sensory systems that are convergent with other nocturnal avian orders. Additionally, we find that a suite of genes associated with vision and circadian rhythm are differentially expressed in blood tissue between nocturnal and diurnal raptors, possibly indicating adaptive expression change during the transition to nocturnality.

**Conclusions:**

Overall, raptor genomes show genomic signatures associated with the origin and maintenance of several specialized physiological and morphological features essential to be apex predators.

**Electronic supplementary material:**

The online version of this article (10.1186/s13059-019-1793-1) contains supplementary material, which is available to authorized users.

## Background

Birds of prey, also known as raptors, are key apex predators in nearly every terrestrial biotic community. Species in this guild comprise a non-monophyletic set of three orders within the core landbird clade, and recent large-scale phylogenomic studies have led to the suggestion that the common ancestor of this clade may have been an apex predator [1]. There are three main orders of birds of prey: Strigiformes (true and barn owls), Falconiformes (falcons and caracaras), and Accipitriformes (eagles, buzzards, hawks, kites, and vultures). Species in each of these three raptor clades are obligate predators with adaptations for hunting, killing, and/or eating meat [2, 3]. Additionally, the common ancestor of owls evolved nocturnality, and most extant owl species are nocturnal, a habit they share with two other avian orders for which we have genome sequences (Caprimulgiformes and Apterygiformes). These independent transitions in lifestyle provide an opportunity to test for patterns of genome evolution that are linked with being raptorial and nocturnal, respectively [3–5].

**Fig. 1:**
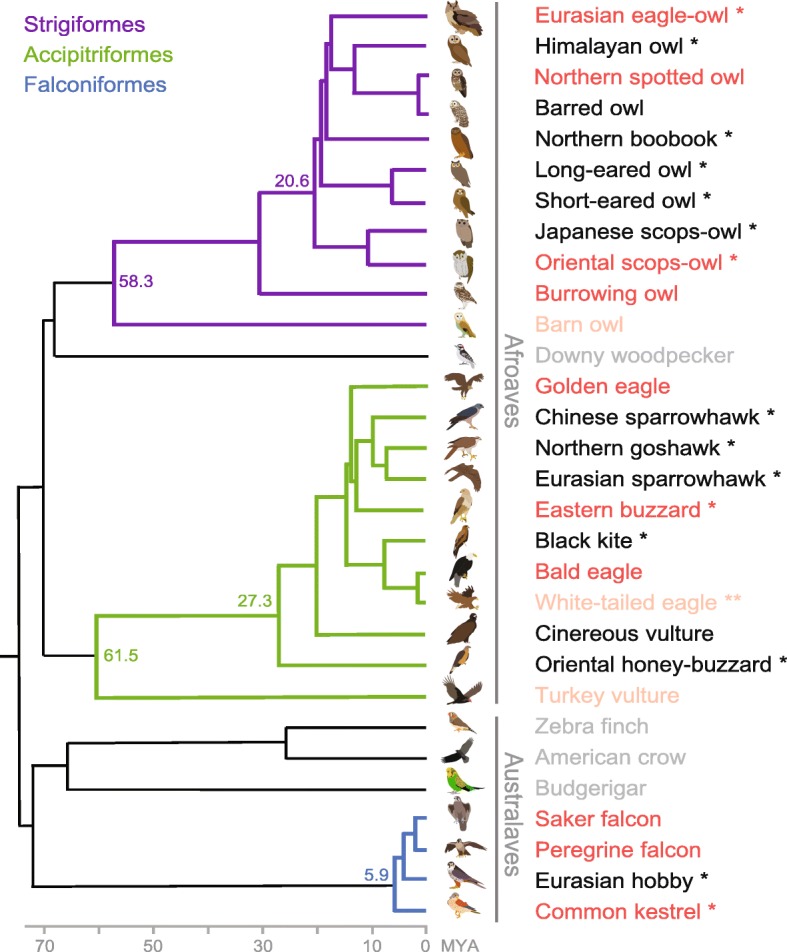
Phylogeny and genomic data of birds of prey. The phylogenetic tree topology was adapted from the Avian Phylogenomics Project [1] and TimeTree database. The estimated divergence time from present (million years ago; MYA) is given at the nodes. Dark red indicates species with higher quality (scaffold N50 length > 1 Mb) genome assemblies, light red indicates species with lower quality genome assemblies, black indicates species for which the whole genome was sequenced, and gray indicates non-raptor species high-quality genome assemblies. One asterisk denotes birds of prey sequenced from this study. The white-tailed eagle (denoted with two asterisks) was previously assembled at low quality and also whole genome sequenced from this study

Genomes have been published for more than 50 avian species, including nine birds of prey (peregrine and saker falcons, bald, white-tailed, and golden eagles, turkey vulture, barn owl, northern spotted owl, and burrowing owl) [3, 6–9]. However, the barn owl, white-tailed eagle, and turkey vulture genomes were assembled at low quality [6], and a detailed comparative evolutionary analysis was performed only for the falcons [3]. Here, we report new high-quality whole-genome reference sequences of four raptor species (Eurasian eagle-owl [*Bubo bubo*] and oriental scops owl [*Otus sunia*] in Strigiformes, eastern buzzard [*Buteo japonicus*] in Accipitriformes, and common kestrel [*Falco tinnunculus*] in Falconiformes) with a set of raptor whole-genome and transcriptome data, extending the genomic coverage of raptors (Fig. 1, Additional file 1: Figure S1 and Tables S1, S2, and S3). Our investigation revealed numerous genomic signatures of evolution that are shared among the three raptor orders or that appear to be associated with nocturnal adaptations of owls.

## Results and discussion

### Raptor genome sequencing and assembly

We applied whole-genome shotgun sequencing and de novo assembly strategies [6, 10–12] to build reference genomes of the four raptor species (Eurasian eagle-owl, oriental scops owl, eastern buzzard, and common kestrel). The extracted DNA samples from wild individuals were sequenced using Illumina HiSeq platforms at high coverage (> 185×) using various insert sizes of short-insert (170 bp, 500 bp, and 700 bp for the two owls and eastern buzzard, and 350 bp and 550 bp for the common kestrel) and long-mate pair libraries (2 Kb, 5 Kb, 10 Kb, and 15 Kb; Additional file 1: Tables S4 and S5). The four raptor genomes showed relatively higher levels of genomic diversity compared to the previously assembled genomes of eagles and falcons (Additional file 1: Figures S2 and S3). Therefore, we tried to assemble reference genomes of the four raptor species using both SOAPdenove2 [10] and Platanus [11] software in various conditions (Additional file 1: Tables S6, S7, and S8). Protein-coding genes (~ 16,000 to 18,000 genes) for these assemblies were predicted by combining de novo and homologous gene prediction methods with whole blood transcriptome data (Additional file 1: Table S9). By assessing assembly statistics, transcript mapping results, and single-copy ortholog mapping results (Additional file 1: Tables S7, S8, and S10), we obtained the final reference genomes for the four raptor species at a high quality, resulting in scaffold N50 sizes from 7.49 to 29.92 Mb; we defined as high-quality genome if the scaffold N50 length is > 1 Mb and as low-quality genome if scaffold N50 length is < 1 Mb, similar to the previous studies [1, 6] (Additional file 1: Table S11). Roughly 9.2% of the raptor genomes were predicted as transposable elements (Additional file 1: Table S12), consistent with the composition of other avian genomes [6]. Additionally, we sequenced the whole genome and blood transcriptome from another 12 raptors (five owls, six accipitrids, and a falconid) and four non-raptor birds (Additional file 1: Tables S11, S13, S14, and S15), most of which were sequenced for the first time. The whole-genome sequences (WGS) of the 12 additional raptors and four non-raptor birds were not assembled, but aligned to the reference genomes of the closely related species for comparison purposes to remove possible bias derived from a small number of raptor/nocturnal species genomes; the whole genome sequenced but not assembled genomes were hereinafter referred to as WGS.

### Evolutionary analysis of raptors compared to non-raptor birds

**Fig. 2:**
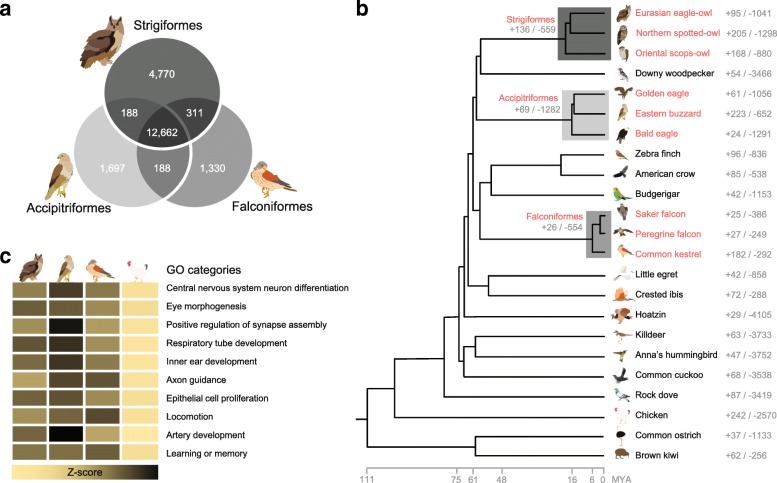
Relationship of birds of prey to other avian species. **a** Venn diagrams of orthologous gene clusters in the birds of prey. Orthologous gene clusters were constructed using 25 avian genomes. Only raptor gene clusters are displayed. **b** Gene expansion or contraction in the 23 high-quality avian species The numbers near order and species names indicate the number of gene families that have expanded (+) and contracted (−) in each branch and species. Species in red are birds of prey. **c** Heatmap of enriched Gene Ontology (GO) categories for raptor common GC3-biased genes. Bird icons from the left to the right indicate Strigiformes, Accipitriformes, Falconiformes, and non-raptor birds. *Z*-scores for the average of normalized GC3 percentages are shown as a yellow-to-black color scale

To identify the genetic basis of predation and nocturnality in raptors, we performed in-depth comparative evolutionary analyses for 25 birds of prey (including 10 nocturnal owls and 15 diurnal raptors) and 23 non-raptor bird species (including nocturnal brown kiwi [12] and chuck-will’s-widow [6], and other avian representatives genome assembled at a high quality [13–16] (Additional file 1: Figure S4 and Tables S1, S2 and S11). First, gene family clusters were constructed using a total of 25 assembled avian genomes (both 23 high- and 2 low-quality genomes; Additional file 1: Tables S11 and S16). Of the 29,115 orthologous gene families found in the 25 avian genomes, 12,662 were found in the all raptor genomes (Fig. 2a and Additional file 1: Figure S4). Based on the comparison of orthologous gene families among the only 23 high-quality avian genomes, 136 expanded and 559 contracted, 69 expanded and 1282 contracted, and 26 expanded and 554 contracted gene families were found in the common ancestors of Strigiformes, Accipitriformes, and Falconiformes, respectively, compared with the common ancestors of each raptor order and its sister group (Fig. 2b). Birds have evolved to employ many different strategies to obtain food, and raptors are specialized for hunting [2, 3, 7]. Several molecular signatures were shared by the three raptor orders, and the ancestral branches of these orders each showed an expansion of gene families associated with sensory perception of sound, regulation of anatomical structure morphogenesis, postsynaptic density and specialization, and learning functions (*P* < 0.05, Fisher’s exact test; Additional file 1: Table S17).

To further examine the shared evolutionary adaptations related to avian predatory lifestyles, we identified selection signatures shared by the three orders of birds of prey compared to the non-raptor birds (both high- and low-quality genomes) at the gene sequence level, which possibly reflects their shared requirement for highly developed sensory systems, efficient circulatory and respiratory systems, and exceptional flight capabilities necessary to capture prey [2–5, 7, 8]. Based upon *d*_*N*_/*d*_*S*_ ratio calculation [17, 18], only *RHCE* and *CENPQ* genes were commonly found as positively selected genes (PSGs) in the three raptor ancestral branches of the Strigiformes, Accipitriformes, and Falconiformes (Additional file 2: Datasheets S1, S2, and S3). In addition, we identified three genes as positively selected in the ancestral branches of two raptor orders (*SFTPA1* in the Strigiformes and Falconiformes; *TFF2* and *PARL* in the Strigiformes and Accipitriformes). A lung surfactant protein encoded by *SFTPA1* plays an essential role in the defense against respiratory pathogens and normal respiration [19]. *TFF2* gene encodes a protein that mediates gastric wound repair and inhibits gastric acid secretion [20]. Finally, we found that 148 genes showed accelerated *d*_*N*_/*d*_*S*_ in the raptor ancestral branches (Additional file 1: Table S18). Of these, *SLC24A1*, *NDUFS3*, and *PPARA* encode proteins that play roles in visual transduction cascade, mitochondrial membrane respiratory chain, and lipid metabolism, respectively [19, 21, 22].

It has been suggested that genes with elevated frequencies of guanine-cytosine at the third codon position (GC3) are more adaptable to external stresses, through providing more targets for de novo methylation that affect the variability of gene expression [23]. Therefore, we analyzed the GC3 content in the three raptor orders, and we found that regulation of nervous system development, central nervous system neuron differentiation, and locomotion-associated genes showed high GC3 bias (Fig. 2c, Additional file 1: Figure S5, Table S19, and Additional file 2: Datasheet S6). In the highly conserved genomic regions (HCRs) among species belonging to the same order, 79 functional categories were commonly enriched in the three raptor orders (Additional file 1: Tables S20, S21, S22, S23, S24, S25, S26, S27, S28, and S29). Among these categories, eye, sensory organ, muscle organ, epithelium, and limb development functions were commonly conserved in the three raptor orders, but not in Passeriformes (a control avian order in this analysis), suggesting that those functions are important in raptors for their predatory lifestyle.

### Evolutionary analysis of nocturnal birds compared to diurnal birds

Since several avian clades have adapted to a nocturnal lifestyle independently, the comparative method can be used to identify genes underlying convergent phenotypes that are associated with nocturnal adaptation [5]. When comparing the gene families among the 23 high-quality avian genomes, two nocturnal bird groups (the ancestral branch of owls and brown kiwi) shared an expansion of gene families associated with synapse organization, sensory perception of chemical stimulus and sensory perception of smell functions (*P* < 0.05; Additional file 1: Tables S30 and S31). As expected, gene families associated with vision were commonly contracted in the nocturnal birds, when comparing gene family sizes between the extant species (Additional file 1: Tables S32 and S33). Specifically, gene loss of the violet/ultraviolet-sensitive opsin *SWS1* (*OPN1SW*) was found in all of the nocturnal bird genomes, as previously reported [4, 24].

Compared to the diurnal birds, the nocturnal birds (including two low-quality nocturnal species genomes: barn owl and chuck-will’s-widow) also showed common selection signatures likely linked to their adaptation to a nocturnal environment. A total of 14 PSGs were shared among the three nocturnal groups, and 98 PSGs were shared by at least two nocturnal bird groups (Additional file 2: Datasheets S1, S4, and S5). The shared PSGs were overrepresented in the detection of mechanical stimulus involved in sensory perception of sound, wound healing, and skin development functions (Additional file 1: Table S34), although the enrichment did not pass the false discovery rate criterion. Interestingly, at least one of two wound healing-associated genes (*TFF2* and *COL3A1*) [25, 26] was found to be positively selected in the nocturnal birds. Additionally, six genes (*RHO*, *BEST1*, *PDE6B*, *RPE65*, *OPN4-1*, and *RRH*) involved in light detection, and *RDH8* that is involved in retinol (vitamin A_1_) metabolism [19, 27], showed accelerated *d*_*N*_/*d*_*S*_ in the nocturnal birds (Additional file 1: Table S34). It is well-known that rhodopsin encoded by *RHO* is a light-sensitive receptor and thus enables vision in low-light conditions [28]. Notably, *RHO* also showed a high level of GC3 biases in the nocturnal birds (Additional file 2: Datasheet S7). Furthermore, *RPE65* encodes a protein that is a component of the vitamin A visual cycle of the retina, while *PDE6B* plays a key role in the phototransduction cascade and mutations in this gene result in congenital stationary night blindness. In addition, melanopsin encoded by *OPN4-1* is a photoreceptor required for regulation of circadian rhythm [19, 27]. We also found that only *SLC51A* gene possesses specific amino acid sequences to the nocturnal birds (Additional file 1: Figure S6). *SLC51A*, also known as *OST-α*, is essential for intestinal bile acid transport [29], and it has been suggested that the bile acids affect the circadian rhythms by regulating the expression level of circadian clock-associated gene families [30, 31]. Interestingly, burrowing owl (*Athene cunicularia*), which is known as one of diurnal/crepuscular owls, showed a different sequence alteration pattern from the other nocturnal or diurnal birds in *SLC51A* locus (Additional file 1: Figure S6).

### Sensory adaptations to nocturnal environment

**Fig. 3:**
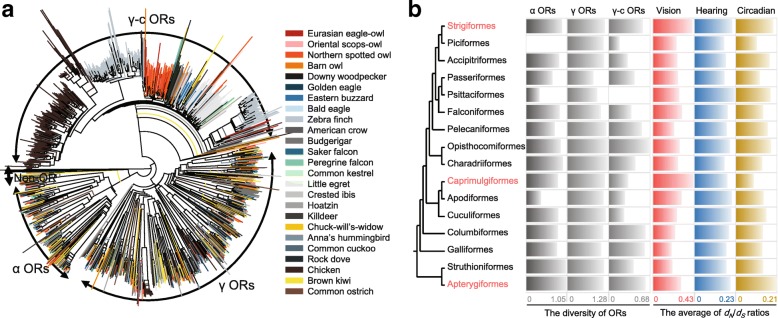
A functional trade-off of sensory systems in nocturnal birds. **a** The phylogeny of the α and γ olfactory receptor (OR) genes identified in 25 avian genomes. The phylogeny information was constructed for intact OR genes only using ClustalW2 software. Colors in the label means different avian species. **b** Selection constraints on sensory systems. Values for α, γ, and γ-c ORs are the diversity of ORs in each clade. For avian orders including two or more genomes (Strigiformes, Accipitriformes, Passeriformes, Falconiformes, and Pelecaniformes), the average diversity values were used. The diversity of α ORs in Piciformes and γ-c ORs in Psittaciformes were not calculated as the number of identified OR genes were smaller than two. Values for vision, hearing, and circadian rhythm are *d*_*N*_/*d*_*S*_ ratios of each set of sensory system-associated genes. For avian orders including two or more genomes, *d*_*N*_/*d*_*S*_ ratios of the ancestral branches were used. Three avian orders in red are nocturnal

Modifications of the major sensory systems (not only vision, but also olfaction, hearing, and circadian rhythm) are among the most common changes that occur when shifting from a diurnal to a nocturnal lifestyle [5]. Analysis of the major sensory systems in the nocturnal bird genomes (owls, chuck-will’s-widow, and brown kiwi) revealed evidence of highly developed senses for adaptation to nocturnality. First, vision system-associated genes showed significantly accelerated *d*_*N*_/*d*_*S*_ in the three nocturnal birds compared to diurnal birds (*P* < 0.05; Mann-Whitney *U* test; Fig. 3). Owls and chuck-will’s-widow (Caprimulgiformes) had the highest acceleration in vision-related genes. The total number of functional olfactory receptors (ORs) was not larger in the nocturnal birds than in the diurnal birds. However, the numbers of γ-clade ORs in the nocturnal birds and γ-c-clade ORs in the owls were significantly larger than others (after excluding two outlier species [32] showing extensive γ-c-clade OR expansion, chicken and zebra finch; *P* < 0.05, Mann-Whitney *U* test; Fig. 3 and Additional file 1: Table S36). The diversity of ORs is thought to be related to a detection range of odors [33], and we found that the diversity of α-clade ORs was significantly higher in the nocturnal birds (Additional file 1: Table S37). Additionally, the diversity in the γ-c-clade ORs was much higher in the owls and brown kiwi (Apterygiformes) compared to their sister groups (downy woodpecker in Piciformes and common ostrich in Struthioniformes, respectively), suggesting that increased olfactory abilities evolved repeatedly under nocturnal conditions [5, 12]. Hearing system-associated genes showed a relatively high level of *d*_*N*_/*d*_*S*_ ratio in the owls and brown kiwi; interestingly, two vocal learning species (budgerigar in Psittaciformes and Anna’s hummingbird in Apodiformes) had the first and third most accelerated *d*_*N*_/*d*_*S*_ for hearing-associated genes, which may be linked with their highly developed cognitive abilities [32, 34]. Circadian rhythm-associated genes showed the first and second largest acceleration in the owls and brown kiwi, but the lowest in chuck-will’s-widow, suggesting that these independent instances of adaptation to nocturnality occurred by different mechanisms [5]. Additionally, we found that 33 hearing system- and 18 circadian rhythm-associated genes showed accelerated *d*_*N*_/*d*_*S*_ in the three nocturnal bird groups (Additional file 1: Table S38). Considered together, these results suggest that selection to augment nocturnal vision and other sensory systems predictably compensates for loss of color vision, supporting a functional trade-off of sensory systems in nocturnal birds [4, 5, 12].

Changes in gene expression are thought to underlie many of the phenotypic differences between species [35]. Therefore, we carried out cross-species comparison of gene expression among the blood transcriptomes from 13 raptors (five owls, four accipitrids, and four falconids) and five non-raptor birds (Additional file 1: Tables S11 and S15). We found that several vision-associated genes [19, 27] were differentially expressed in the owls (*P* < 0.05, moderated *t* test; Additional file 1: Figures S7 and S8, and Additional file 2: Datasheets S8, S9, S10, and S11). For example, *PDCL* (lowly expressed) and *WFS1* (highly expressed) genes were differentially expressed specific to the owls. Interestingly, we could also find several circadian rhythm-related genes that were differentially expressed between the nocturnal and diurnal raptors. Three circadian rhythm-associated genes (*ATF4*, *PER3*, and *NRIP1*) were lowly expressed and two genes (*BTBD9* and *SETX*) were highly expressed in the owls, whereas *ATF4* and *SIRT1* in the falconids and *NRIP1* in the accipitrids were highly expressed. These results likely indicate that selectively driven expression switches contributed to nocturnal adaptation of owls [33]. However, the comparison of gene expressions based on blood transcriptome may not represent gene expression profiles of vision system, and therefore, further studies are needed to confirm our results (e.g., analyzing expression profiles of retinal tissue and visual brain regions).

## Conclusions

Our study provides whole-genome assemblies of Eurasian eagle-owl, oriental scops owl, eastern buzzard, and common kestrel, as well as a suite of whole-genome sequencing and transcriptome data from birds of prey. This is the first in-depth genomics study comparing the three raptor orders, and we identified a number of shared molecular adaptations associated with a predatory lifestyle. Furthermore, compared with diurnal birds, owls and other nocturnal birds showed distinct genomic features, especially in sensory systems. At the same time, it is important to note that genome assembly based on short-read sequencing methods could possess incomplete genomic regions, thus causing an erroneous result in comparative evolutionary analyses [36, 37]. Therefore, the candidate genes identified in this study need to be further confirmed with additional genomic data, and functional studies of candidate genes will be needed to understand the molecular mechanisms of adaptation. In overall, these results provide a genome-wide description and gene candidates of adaptations that have allowed each of these three raptor groups to evolve into diverse, ecologically dominant apex predators.

## Methods

### Sample and genome sequencing

All blood samples used for genome and transcriptome sequencing were collected from individuals being euthanized due to poor survival during wound treatment of rescued animals, except blood samples of *A. flammeus*, *O*. *semitorques*, and *P. ptilorhynchus* that were obtained from the live individuals during a medical check-up at the wildlife rescue center. Muscle tissue samples collected in 2017 were obtained from the fresh carcasses (Additional file 1: Table S3).

To build reference genome assemblies of the four raptor species (Eurasian eagle-owl, oriental scops owl, eastern buzzard, and common kestrel), we constructed 11 genomic libraries with various insert sizes (Illumina short-insert and long-mate pair libraries) for each species, according to the manufacturer’s protocol. The libraries were sequenced using Illumina HiSeq platforms (Additional file 1: Table S4). The remaining 12 raptor and four non-raptor bird samples were sequenced using Illumina HiSeq platforms with short-insert libraries (Additional file 1: Table S11c). Blood transcriptomes of ten raptors and four non-raptor birds were sequenced using Illumina HiSeq platforms according to the manufacturer’s instructions (Additional file 1: Table S11d).

### Genome assembly and annotation

To assemble the raptor genomes, PCR duplicated, sequencing and junction adaptor contaminated, and low-quality (Q20) reads were filtered out. The short-insert and long-mate library reads were trimmed into 90 bp and 50 bp, respectively, to remove low-quality bases at the ends of the reads (Additional file 1: Table S5). As the four raptor genomes showed relatively higher levels of genomic diversity (Additional file 1: Figures S2 and S3), we assembled reference genomes of the four raptor species using both SOAPdenove2 [10] and Platanus [11] software; the Platanus assembler is more efficient for highly heterozygous genomes [11]. When performing the SOAPdenovo2 assembler, we applied various *K*-mer values (33, 43, 53, and 63) to obtain fragments with long contiguity. To reduce the number of gaps in the scaffolds, we closed the gaps using the short-insert library reads in two iterations. To correct base-pair-level errors, we performed two iterations of aligning the short-insert library reads to the gap-closed scaffolds using BWA-MEM [38] and calling variants using SAMtools [39]. In this process, homozygous variants were assumed as erroneous sequences from the assembly process, and thus substituted for the correction purpose (Additional file 1: Table S7).

To select final high-quality reference assemblies for the four raptors, we annotated all assemblies and evaluated the quality of each assembly. We first searched the genomes for tandem repeats and transposable elements (Additional file 1: Table S9) using Tandem Repeats Finder (version 4.07b) [40], Repbase (version 19.03) [41], RepeatMasker (version 4.0.5) [42], RMBlast (version 2.2.28) [43], and RepeatModeler (version 1.0.7) [44]. The protein-coding genes were predicted by combining de novo and homology-based gene prediction methods with the blood transcriptome data for each assembly. For the homology-based gene prediction, we searched for avian protein sequences from the NCBI database using TblastN (version 2.2.26) [45] with an *E* value cutoff of 1E−5. The matched sequences were clustered using GenBlastA (version 1.0.4) [46] and filtered by coverage and identity of > 40% criterion. Gene models were predicted using Exonerate (version 2.2.0) [47]. For the de novo gene prediction, AUGUSTUS (version 3.0.3) [48] was used with the blood transcriptome for each species. We filtered out possible pseudogenes having premature stop codons and single exon genes that were likely to be derived from retro-transposition (Additional file 1: Table S9). The assembly and gene annotation qualities were assessed by aligning independently de novo assembled transcripts using the Trinity software [49] and by searching for evolutionary conserved orthologs using BUSCO software [50] (Additional file 1: Tables S8 and S10). By considering the assembly statistics (e.g., N50 values and assembled sequence length) and the completeness of the genome assembly, final high-quality reference assemblies for the four raptors were obtained. Genome, transcriptome, and protein sequences for other comparison species were downloaded from the NCBI database. Genes with possible premature stop codons were excluded in the comparative analyses. The northern spotted owl’s genome and protein sequences were acquired from the Zenodo linked in the published paper [8].

### Comparative evolutionary analyses

Orthologous gene families were constructed for avian genomes using the OrthoMCL 2.0.9 software (Additional file 1: Figure S4) [51]. To estimate divergence times of the 25 avian representatives, protein sequences of the avian single-copy gene families were aligned using the MUSCLE program [52]. The poorly aligned regions from the alignments were trimmed using the trimAl software [53]. The divergence times were estimated using the MEGA7 program [54] with the phylogenetic tree topology of published previous studies [1, 6] and the TimeTree database [55]. When we calculated the divergence times among the 23 species with high-quality reference genomes (Fig. 2b), the date of the node between chicken and rock dove was constrained to 98 million years ago (MYA), chicken and brown kiwi was constrained to 111 MYA, and common ostrich and brown kiwi was constrained to 50–105 according to the divergence times from TimeTree. To estimate divergence times among the birds of prey (Fig. 1), the date of the node between downy woodpecker and Eurasian eagle-owl constrained to 61–78 MYA and common kestrel and budgerigar was constrained to 60–80 MYA according to the divergence times from the previous studies [1, 6] and TimeTree; as the divergence times and phylogenetic topologies of the previous studies [1, 6] and TimeTree were quite different, we used the divergence times from the previous studies as the minimum and the divergence times from the TimeTree database as the maximum constraints. A gene family expansion and contraction analysis for the ancestral branches of the three bird of prey orders was conducted using the CAFÉ program [56] with a *P* < 0.05 criterion. As the gene family expansion and contraction analysis can be affected by erroneous genomic regions derived from the assembly process [36, 37], we calculated the mapping depth coverage of genes in the raptor and nocturnal bird genomes, and then filtered out genes having abnormal depth coverage (if the mapping depth coverage of genes is less than half of the average depth coverage [less than quarter of the average depth coverage for genes in sex chromosomal scaffolds] or more than twice of the average depth coverage; Additional file 1: Figure S9). The significantly different gene family sizes of the present nocturnal bird species were identified by performing the Mann-Whitney *U* test (*P* < 0.05).

To identify selection at the gene sequence level, two orthologous gene sets were compiled, as previously reported [3]: the single-copy orthologs among avian species and representative genes from multiple-copy orthologs. The representative genes from multiple-copy orthologs were selected, if all species’ protein sequences are reciprocally best matched to a chicken protein sequence using BLASTp with an *E* value cutoff of 1E−5. PRANK [57] was used to construct multiple sequence alignments among the orthologs. The CODEML program in PAML 4.5 was used to estimate the *d*_*N*_/*d*_*S*_ ratio (non-synonymous substitutions per non-synonymous site to synonymous substitutions per synonymous site) [17]. The one-ratio model was used to estimate the general selective pressure acting among comparison species. The two-ratio model (model = 2) was used to ensure that the *d*_*N*_/*d*_*S*_ ratio is the difference between foreground species (raptors and nocturnal birds, respectively) and other species. Additionally, the *d*_*N*_/*d*_*S*_ ratios for each order-level branch of raptors and nocturnal birds were used to confirm if the foreground *d*_*N*_/*d*_*S*_ ratio is not biased to a specific raptor and nocturnal bird order. The branch-site test was also conducted [18]. Statistical significance was assessed using likelihood ratio tests with a conservative 10% false discovery rate criterion (Additional file 2: Datasheets S1, S2, S3, S4, and S5).

We identified target species-specific amino acid sequences [6]. To filter out biases derived from individual-specific variants, we used all of the raptor WGS data by mapping to the Eurasian eagle-owl genome for Strigiformes, the eastern buzzard genome for Accipitriformes, and the common kestrel genome for Falconiformes. The mapping was conducted using BWA-MEM, and consensus sequences were generated using SAMtools with the default options, except the “-d 5” option (Additional file 1: Table S13). When we identified the specific amino acid sequences, protein sequences of other birds from the NCBI database were also compared. We also checked multiple sequence alignments manually to remove artifacts. To identify genetic diversity based on heterozygous SNV rates, variants were also called using Sentieon pipeline [58] with the default options, except the “--algo Genotyper” option (Additional file 1: Table S14). The heterozygous SNV rates were calculated by dividing the total number of heterozygous SNVs by the length of sufficiently mapped (> 5 depth) genomic regions (Additional file 1: Figure S3).

To identify HCRs in the three raptor orders and Passeriformes, we scanned genomic regions that show significantly reduced genetic variation by comparing variations of each window and whole genome as previously suggested [59]. In the case of Passeriformes, whole-genome data of four Passeriformes species (medium ground-finch, white-throated sparrow, common canary, and collared flycatcher) were mapped to the zebra finch genome assembly, and then variants were identified using the same methods used for the three raptor orders. Genetic variation was estimated by calculating the number of different bases in the same order genomes within each 100-Kb window. *P* value was calculated by performing Fisher’s exact test to test whether the genetic variation of each window is significantly different from that of the whole genome. Only adjusted *P* values (*q* values) [60] of < 0.0001 were considered significant. As both ends of scaffolds have usually incorrect sequences and many gaps, the middle 10 Kb of each significantly different window was only considered as HCRs (Additional file 1: Table S20).

For functional enrichment tests of candidate genes, GO annotations of chicken, zebra finch, turkey, flycatcher, duck, anole lizard, and human genomes were downloaded from the Ensembl database [61] and used to assign the avian protein-coding genes with GO categories. A KEGG pathway was assigned using KAAS [62]. Functional information of candidate genes was retrieved from the GO, KEGG, UniProt [63], and GeneCards [19] databases.

### De novo transcriptome assembly and differentially expressed genes

The blood transcriptome data were assembled using Trinity software [49]. Contaminated transcripts were searched for bacteria and fungi sequence from the Ensembl database using BLASTN and filtered by identity of > 95% and *E* value cutoff of 1E−6 criteria. Coding sequence (CDS) were predicted using TransDecoder [49, 64]. To identify differentially expressed genes, RNA reads were aligned to the reference genome (species whole genome assembled) or the assembled transcripts (species without reference genome) using TopHat2 software [65]. The number of reads that were mapped to orthologous genes was counted using HTSeq-0.6.1 software [66] and then converted into RPKM (reads per kilobase per million mapped reads) value (Additional file 1: Table S15). The RPKM values were normalized with the Trimmed Mean of M values (TMM) [67] correction using the R package edgeR [68]. The significance of differential expression was calculated by the moderated *t* test [69] (ebayes function) using the R package limma (*P* < 0.05; Additional file 2: Datasheets S8, S9, S10, and S11) [70].

### Sensory system-associated gene analysis

To compare the olfactory sense across avian clades, we collected a total of 215 chicken olfactory receptor (OR) gene sequences (functional only) from a previously published paper [71]. These ORs were then searched against the 25 avian species genomes using TblastN with default parameters. For OR candidates lacking start/stop codons, we searched 90 bp upstream to find start codons and 90 bp downstream to find stop codons. After collecting sequences for each species, the CD-HIT program [72] was used to remove redundant sequences with an identity cutoff of 100%. A Pfam [73] search against sequences using hmmer-3.1 program [74] with an *E* value cutoff of 1.0 was used to identify sequences that contained 7tm_4 domain. To assign OR clades and filter out non-OR genes, the multiple sequence alignments and phylogenetic analysis were conducted with previously clade-assigned OR and non-OR genes of human, anole lizard, and chicken [75] using ClustalW2 program [76]. The remaining OR candidates were classified into three categories: (1) intact genes with normal start and stop codons and longer than 215 amino acid sequences, thus can code for seven transmembrane domains; (2) partial genes without start and/or stop codons; and (3) pseudogenes with frameshift mutations and/or premature stop codons (Additional file 1: Table S36). OR genes have evolved by multiple duplications and display a large number of pseudogenes, which makes the assembly of OR regions challenging and complicates the annotation process of OR genes [5, 12, 77, 78]. To overcome these issues, we also calculated the diversity of OR genes from the clade-assigned intact genes by Shannon entropy [79] using BioEdit [80] as previously suggested [5, 12] (Additional file 1: Table S37). Amino acid positions with above 20% of gaps were excluded, and entropy was averaged across all amino acid positions.

The vision system-associated genes were retrieved from previous studies [5, 13]. Hearing-associated genes were retrieved from the AmiGO database [81] using GO categories related to hearing [5]. Circadian rhythm-related genes were retrieved from the AmiGO database using “biorhythm/circadian” as search keywords. The protein sequences with the same gene name were aligned using ClustalW2 and manually inspected one by one for quality. A total of 402 sensory system-associated genes (64 genes for vision, 219 genes for hearing, and 133 genes for circadian rhythm) shared by the brown kiwi, chuck-will’s-widow, and at least two Strigiformes were included for selection constraint (the *d*_*N*_/*d*_*S*_ ratio) analyses (Additional file 1: Table S38).

## Additional files

Additional file 1: **Figures S1-S9**, **Tables S1-S38**, and Supplementary Methods. Supplementary figures, tables, and methods supporting the manuscript. (PDF 4139 kb)

Additional file 2: **Datasheets S1-S11.** Supplementary datasheets supporting the manuscript. (XLS 1689 kb)

Additional file 3: Review history. (DOCX 48 kb)

## Data Availability

All the sequences reported in this study are deposited into the NCBI Sequence Read Archive under the accession number PRJNA431699 [82].
