# Supplementary material for: Raptor genomes reveal evolutionary signatures of predatory and nocturnal lifestyles
Source: Genome Biol. 2019 Aug 29;20:181. doi: 10.1186/s13059-019-1793-1 (PMC6714440; doi:10.1186/s13059-019-1793-1)
Supplement: Supplementary file 1 — Figures S1-S9, Tables S1-S38, and Supplementary Methods. Supplementary figures, tables, and methods supporting the manuscript. (PDF 4139 kb) [file 13059_2019_1793_MOESM1_ESM.pdf]

Additional file 1

List of Supplementary Figures

Figure S1. Species identification for the new 20 avian species sequenced 4

Figure S2. *K*-mer (*K*=17) analyses of 20 avian genomes sequenced in the present study 10

Figure S3. Genetic diversity in 25 bird of prey species 12

Figure S4. Composition of avian orthologous genes 13

Figure S5. Genomic context among 25 avian species 14

Figure S6. *SLC51A* gene variants in nocturnal birds 15

Figure S7. Differentially expressed genes (DEGs) in the birds of prey species 16

Figure S8. Differentially expressed genes associated with the vision system and circadian rhythm 17

Figure S9. The mapping depth coverage of genes in the birds of prey and nocturnal birds 20

List of Supplementary Tables

Table S1. Bird of prey genome and transcriptome data used in this study 21

Table S2. Non-raptor bird genome and transcriptome data used for comparative evolutionary analysis 22

Table S3. Sampling information on bird species sequenced in this study 23

Table S4. Sequencing library statistics used for the four bird of prey genome assemblies 25

Table S5. Filtered sequence information of the four birds of prey 27

Table S6. 17-mer statistics information for 20 avian species 29

Table S7. Global assembly statistics of the four bird of prey genomes 30

Table S8. Assessment of gene coverage by assembled bird of prey transcripts 31

Table S9. Protein-coding gene prediction statistics for the four birds of prey 32

Table S10. Evaluation of the completeness of bird of prey assemblies and gene sets using single-copy orthologs mapping approach 34

Table S11. Genome and transcriptome data and assembly quality information of avian species used in this study 35

Table S12. Transposable element statistics for the four bird of prey genomes 38

Table S13. Whole genome sequencing and mapping statistics for the birds of prey and non-raptor birds 40

Table S14. Variant statistics for the birds of prey and non-raptor birds 41

Table S15. Blood transcriptome de novo assembly and mapping statistics 42

Table S16. Meta information for the 25 genome-assembled avian species used in this study 43

Table S17. Commonly enriched Gene Ontology (GO) categories of expanded gene families in the ancestral branches of Strigiformes, Accipitriformes, and Falconiformes 44

Table S18. List of genes showing accelerated d_N/d_S in the three raptor orders 45

Table S19. Gene Ontology (GO) enrichment of genes that have a high level of GC3 biases in the bird of prey genomes 48

Table S20. Statistics regarding highly conserved regions in Strigiformes, Accipitriformes, Falconiformes, and Passeriformes 49

Table S21. Commonly enriched Gene Ontology (GO) categories of genes in the highly conserved genomic regions (HCRs) of Strigiformes, Accipitriformes, and Falconiformes 50

Table S22. Commonly enriched KEGG pathways of genes in the highly conserved genomic regions (HCRs) of Strigiformes, Accipitriformes, and Falconiformes 51

Table S23. Strigiformes specific GO enrichment of genes in the highly conserved genomic regions (HCRs) 53

Table S24. Strigiformes specifically enriched KEGG pathways of genes in the highly conserved genomic regions (HCRs) 55

Table S25. Accipitriformes specific GO enrichment of genes in the highly conserved genomic regions (HCRs) 56

Table S26. Accipitriformes specifically enriched KEGG pathways of genes in the highly conserved genomic regions (HCRs) 57

Table S27. Falconiformes specific GO enrichment of genes in the highly conserved genomic regions (HCRs) 58

Table S28. Falconiformes specifically enriched KEGG pathways of genes in the highly conserved genomic regions (HCRs) 59

Table S29. Passeriformes specific GO enrichment of genes in the highly conserved genomic regions (HCRs) 61

Table S30. Commonly enriched Gene Ontology (GO) categories of expanded gene families in the common ancestor of Strigiformes and brown kiwi 62

Table S31. Commonly enriched Gene Ontology (GO) categories of contracted gene families in the common ancestor of Strigiformes and brown kiwi 63

Table S32. Gene Ontology (GO) enrichment of gene families that were expanded in size the present nocturnal bird species genomes 64

Table S33. Gene Ontology (GO) enrichment of gene families that were contracted in size the present nocturnal bird species genomes 65

Table S34. Gene Ontology (GO) enrichment of PSGs that were shared in two and/or three nocturnal bird groups 66

Table S35. List of genes showing accelerated dN/dS in the nocturnal birds 68

Table S36. Olfactory receptors identified in 25 avian genomes 70

Table S37. The diversity of olfactory receptors in 25 avian genomes 71

Table S38. Sensory system associated genes showing accelerated dN/dS in the nocturnal birds 72

Supplementary Methods

Genome and transcriptome sequencing 73

Species identification 74

Sequence filtering criteria 74

Repeat annotation 75

Supplementary References 76

Supplementary Figures

Falconiformes – *COI* gene

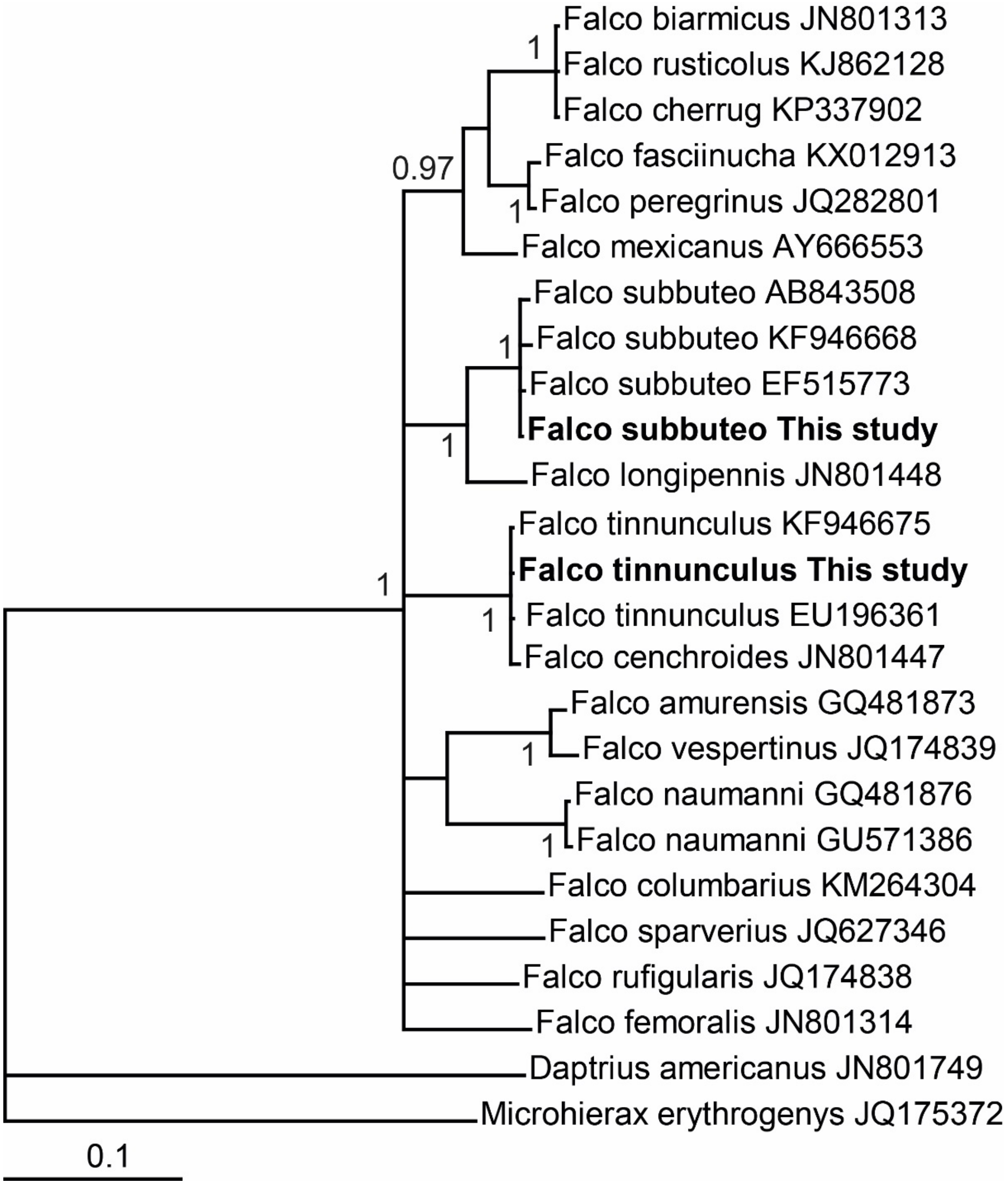

Accipitriformes – *CYTB* gene

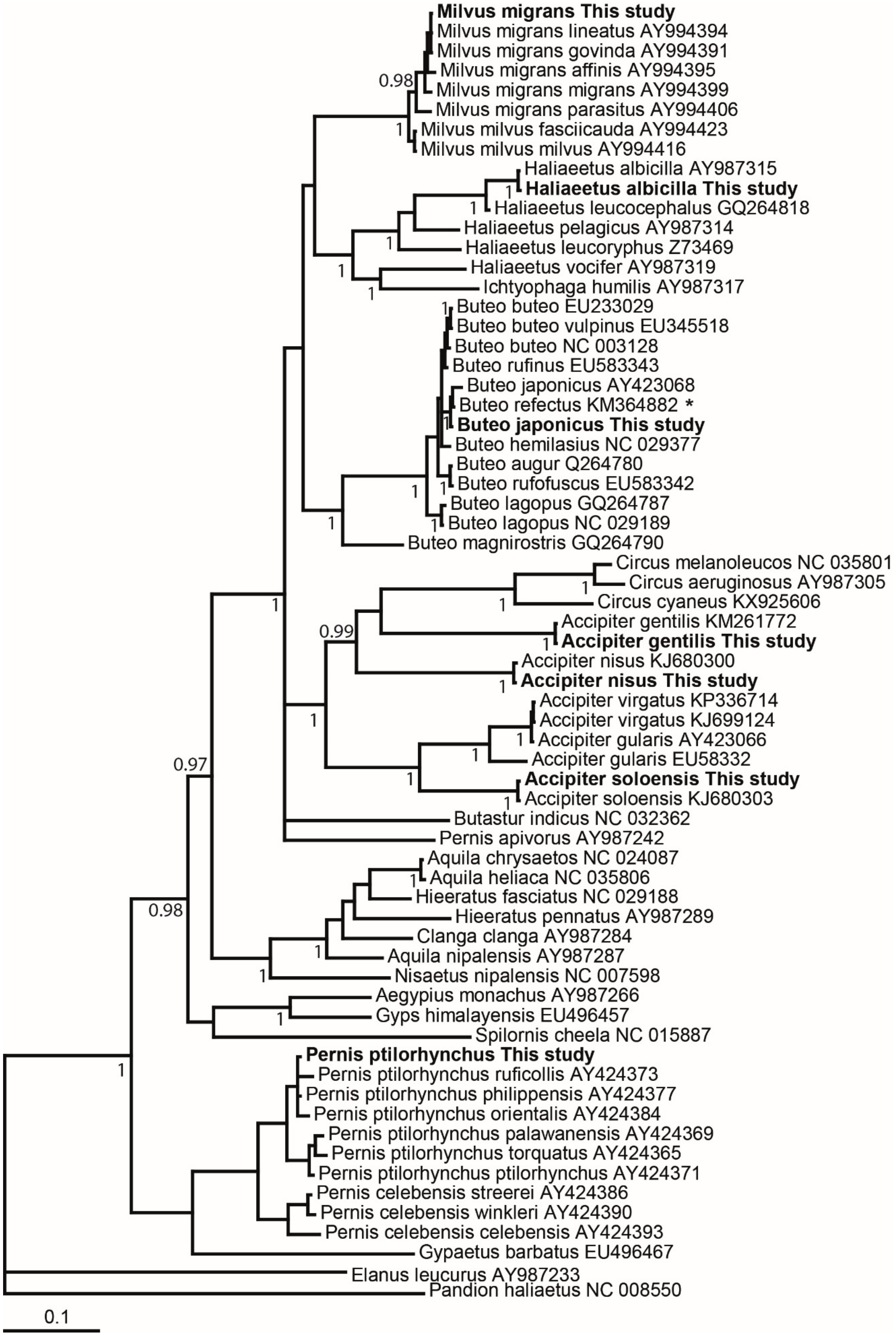

Strigiformes – *CYTB* gene

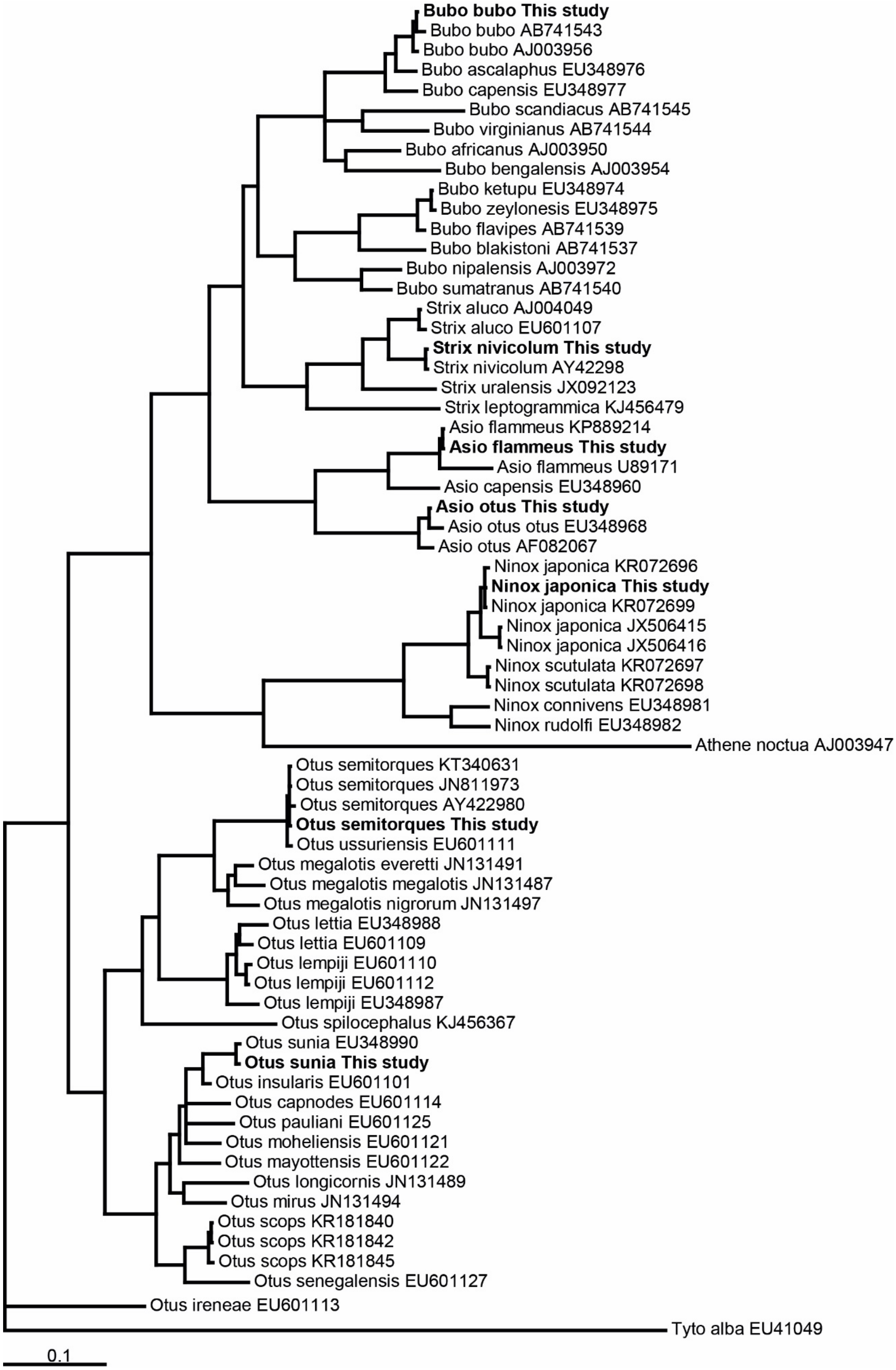

Ardeidae and Threskiornithidae – *COI* gene

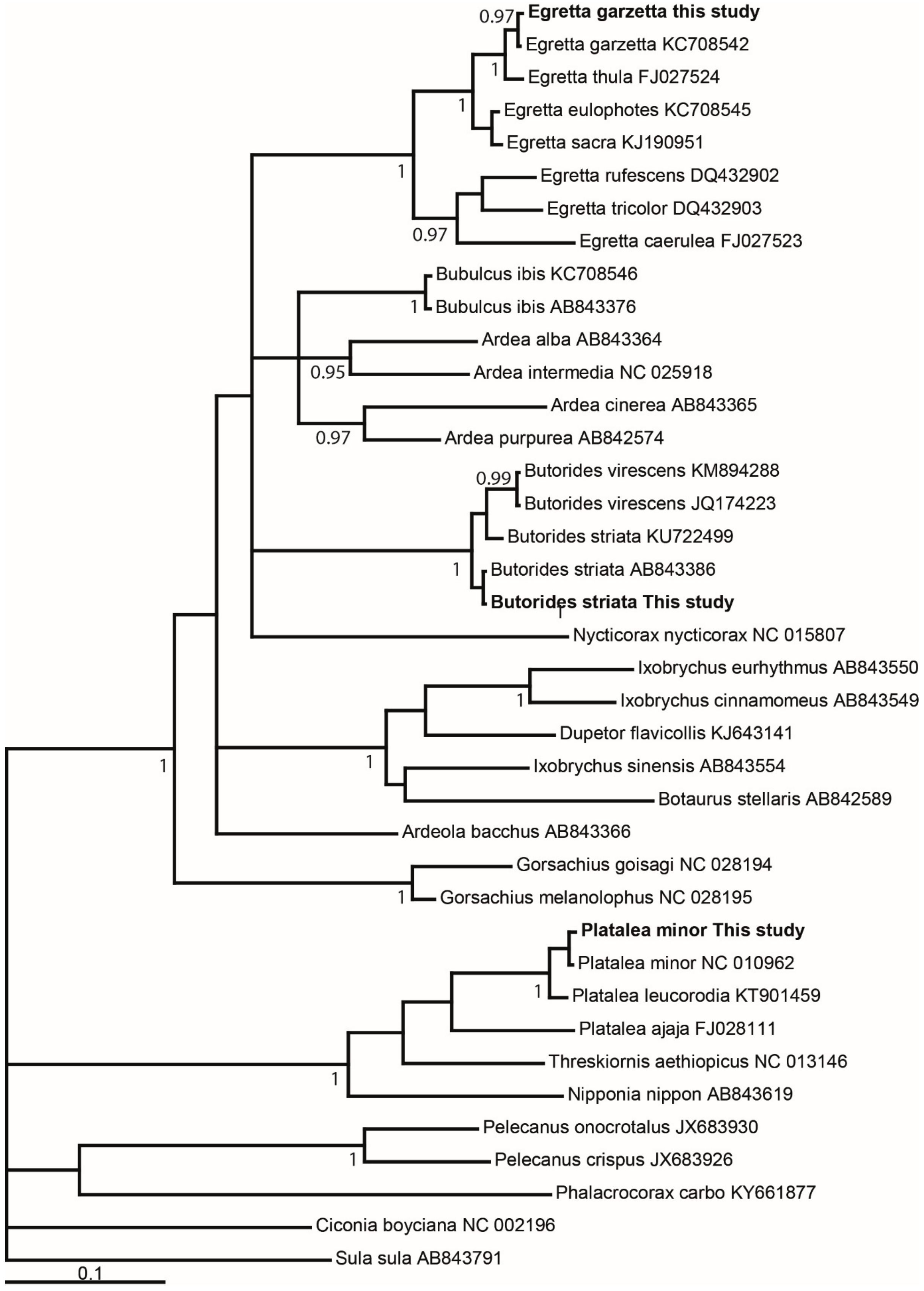

Picidae – *COI* gene

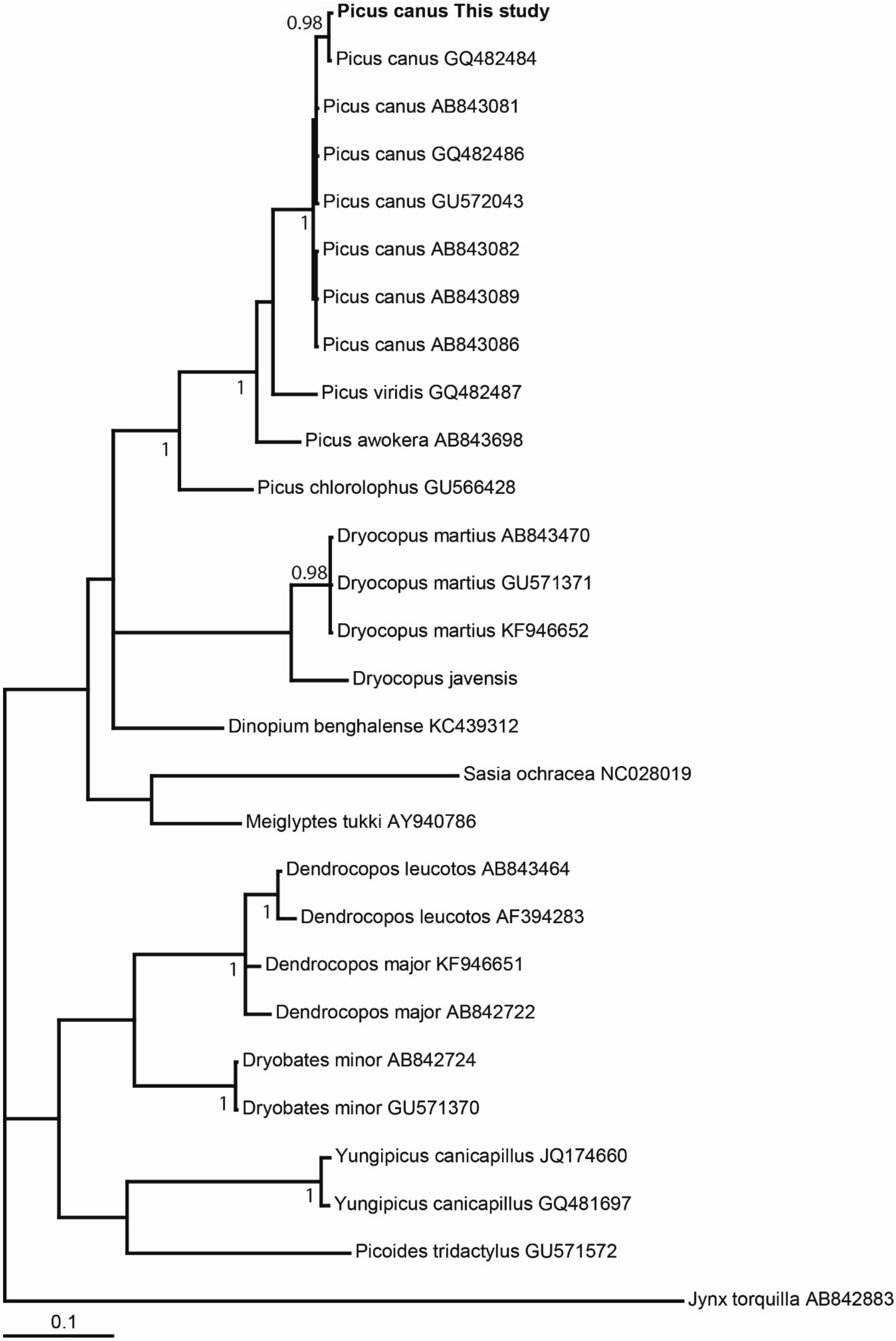

Figure S1. Species identification for the new 20 avian species sequenced. The consensus sequences of avian samples were generated by mapping their reads to previously reported mitochondrial sequences (*COI* and *CYTB* genes) for closely related species. The *COI* gene of common kestrel was sequenced by Sanger method. The names that include "This study" were originally sequenced in this study. ID numbers next to the species name are NCBI accessions. Numbers close to nodes are posterior probabilities. *This sequence was first attributed to *B. buteo burmanicus*, a junior synonym of *B. refectus*; the sampling locality is outside the known range of *B. refectus* and suggests that it is a misidentified *B. japonicus*.

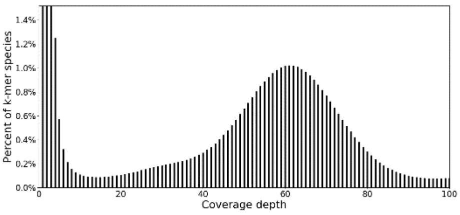

Eurasian eagle-owl

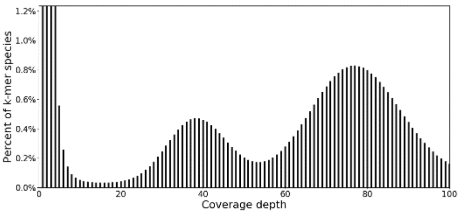

Oriental scops-owl

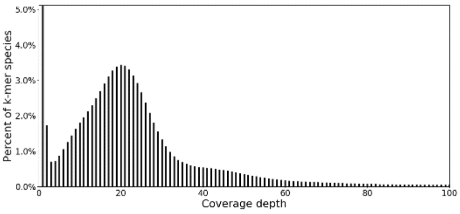

Himalayan owl

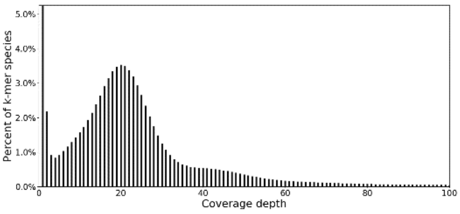

Northern boobook

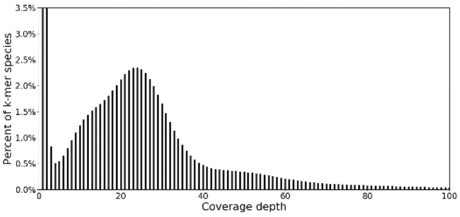

Long-eared owl

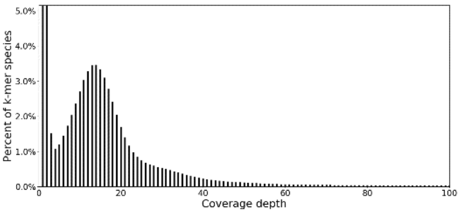

Short-eared owl

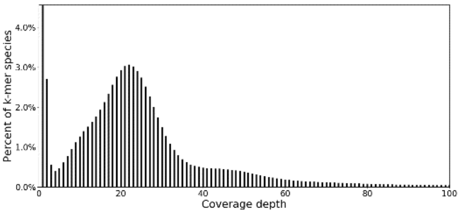

Japanese scops-owl

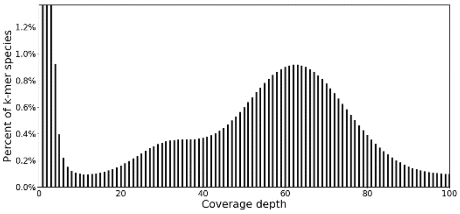

Eastern buzzard

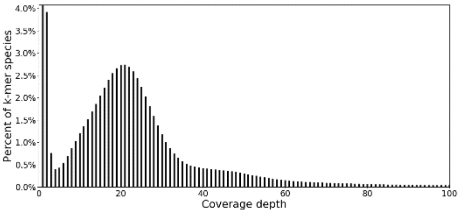

Eurasian sparrowhawk

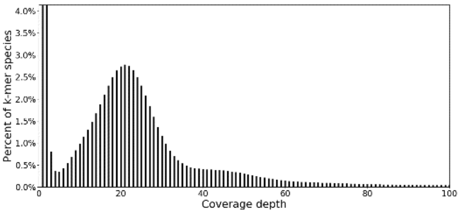

Northern goshawk

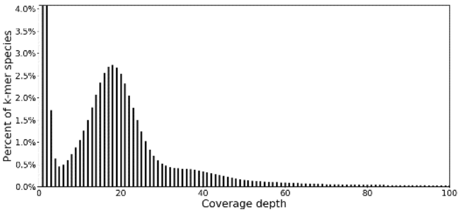

White-tailed eagle

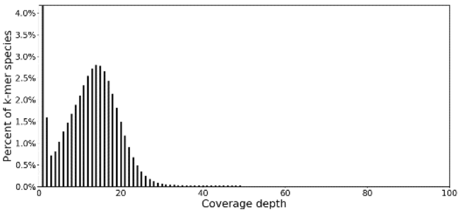

Oriental honey-buzzard

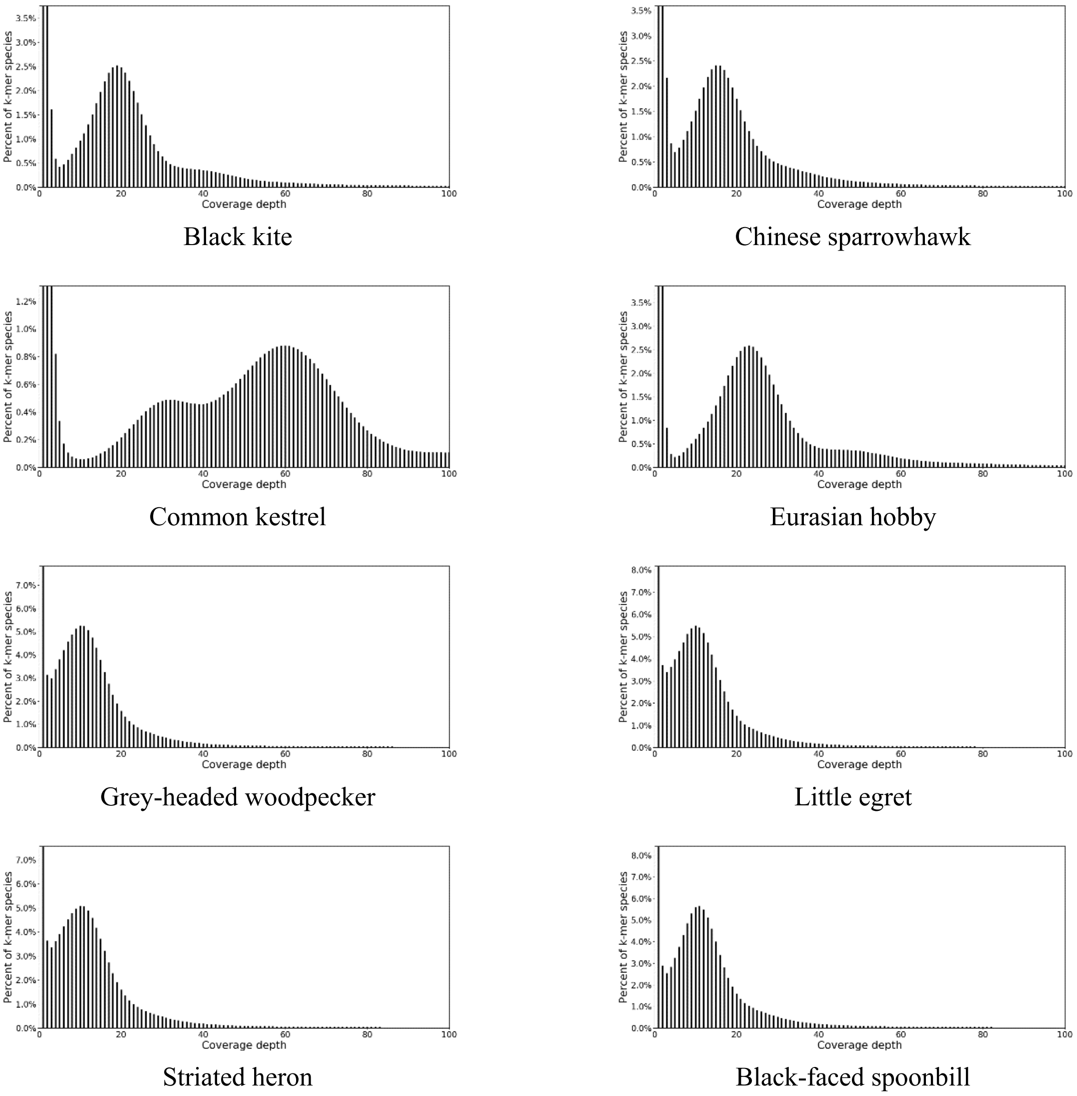

Figure S2. *K*-mer (*K*=17) analyses of 20 avian genomes sequenced in the present study. The *x*-axis represents *K*-mer depth, and the *y*-axis represents the proportion of *K*-mer count at that depth. Individuals with two peaks in the *K*-mer plot represent greater heterozygosity in them.

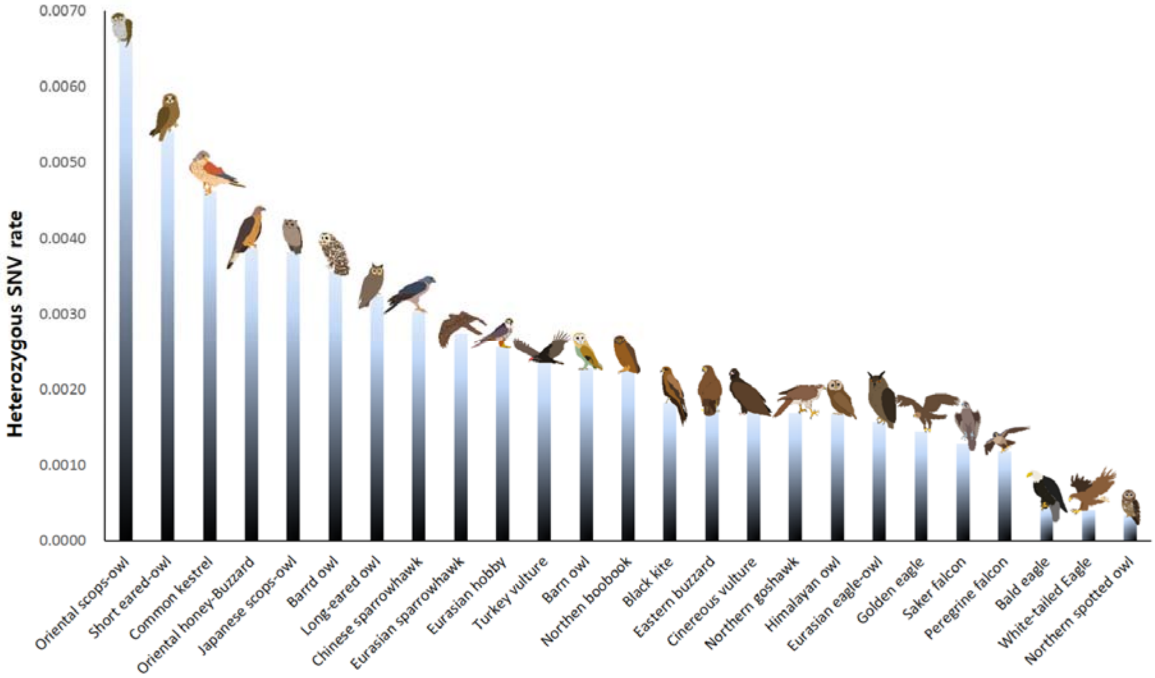

Figure S3. Genetic diversity in 25 bird of prey species. The heterozygous SNVs rates (*y*-axis) were calculated by dividing the total number of heterozygous SNVs by the length of sufficiently mapped (>5 depth) genomic regions. The estimated heterozygous SNVs rates were based on single individuals. The heterozygous SNVs rates can be altered according to which reference assembly is used and the assembly quality.

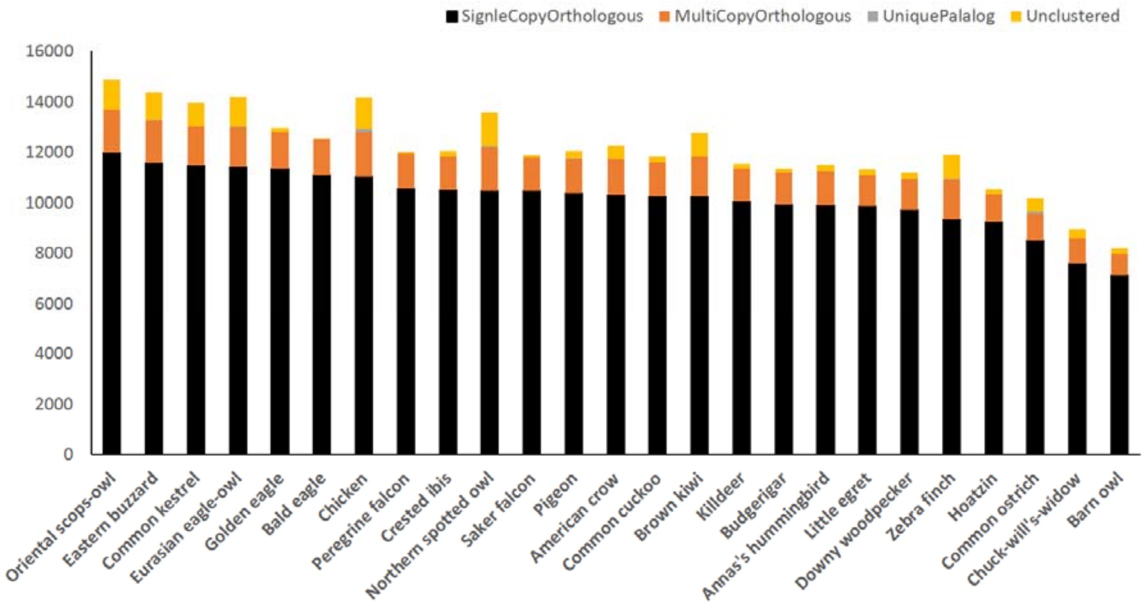

Figure S4. Composition of avian orthologous genes. A comparative representation of orthologous and paralogous genes in 25 avian genomes are shown. Two low-quality genomes (chuck-will's-widow and barn owl) showed low numbers of gene clusters.

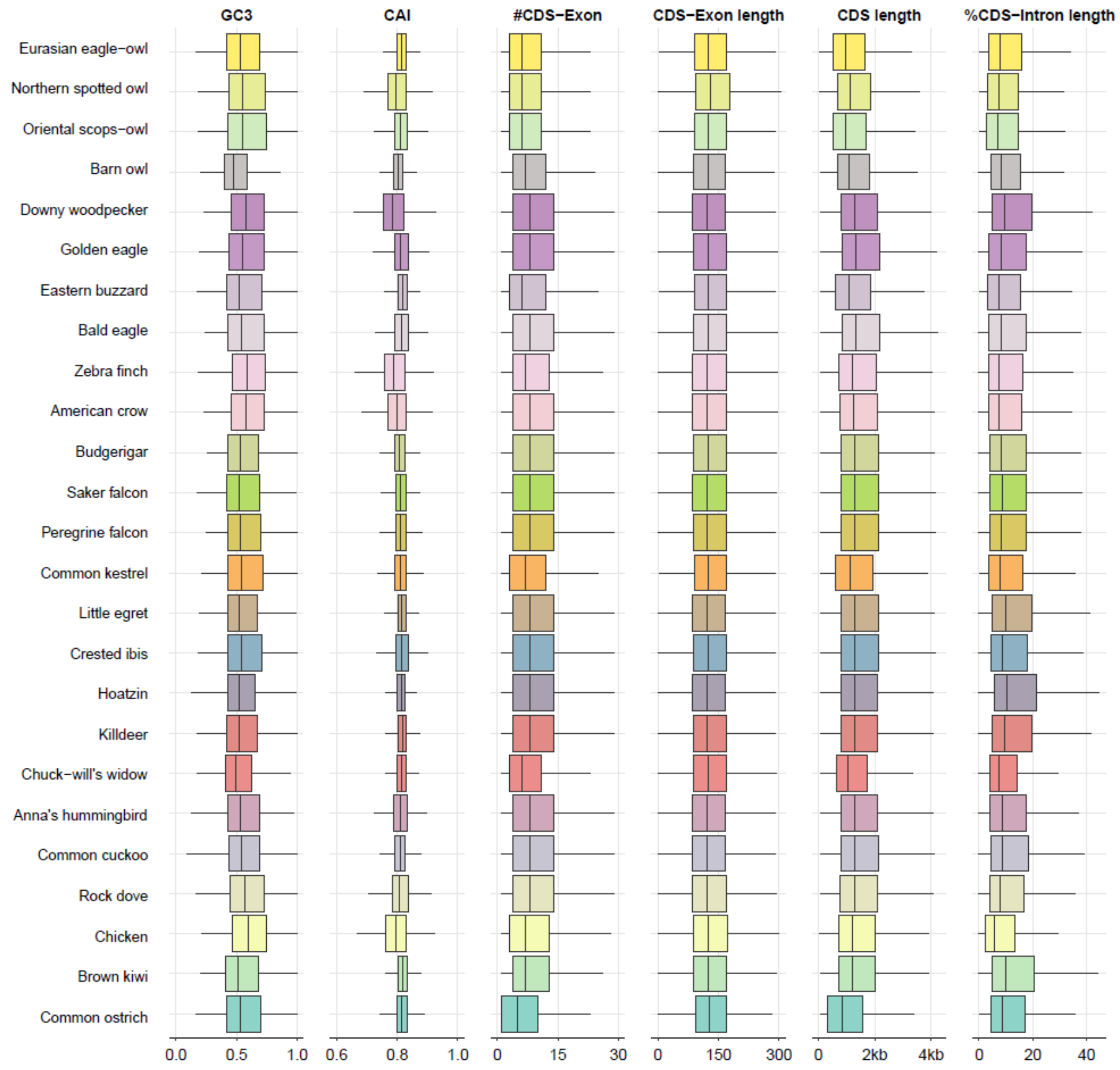

Figure S5. Genomic context among 25 avian species. GC3 ratio is the Guanine-Cytosine ratio at the third codon positions. CAI is the codon adaptation index. # CDS is the average number of CDS. Intron/coding regions is average length of sum of intron length divided by coding region length.

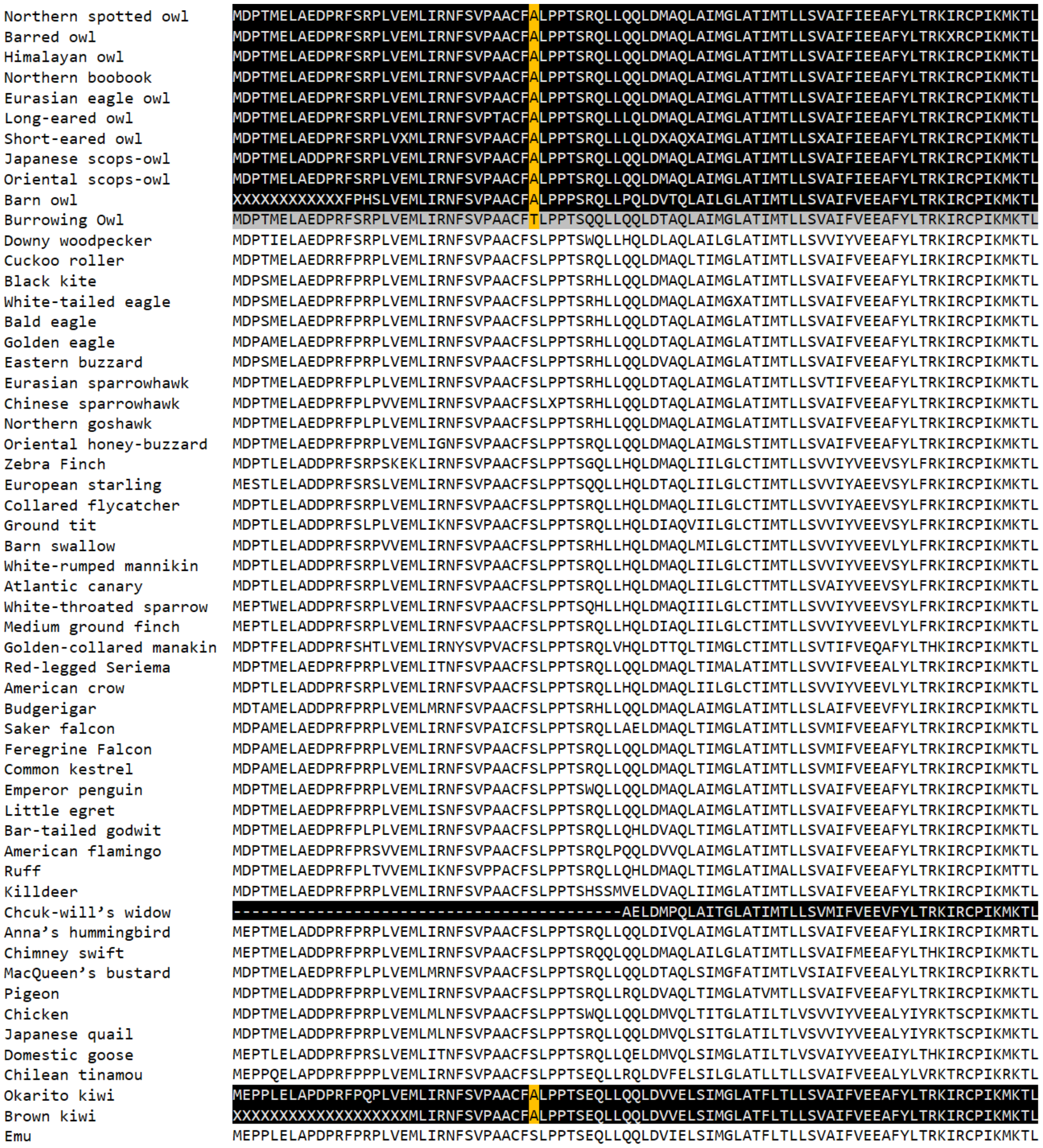

Figure S6. *SLC51A* gene variants in nocturnal birds. An amino acid unique to nocturnal birds and burrowing owl (33th residue in the chicken *SLC51A* protein sequence) is highlighted in yellow. Sequences in the nocturnal birds and burrowing owl are highlighted in black and gray, respectively. We could not find the amino acid residue in the chuck-will’s widow, as *SLC51A* gene is partially annotated in cuck-will’s widow genome.

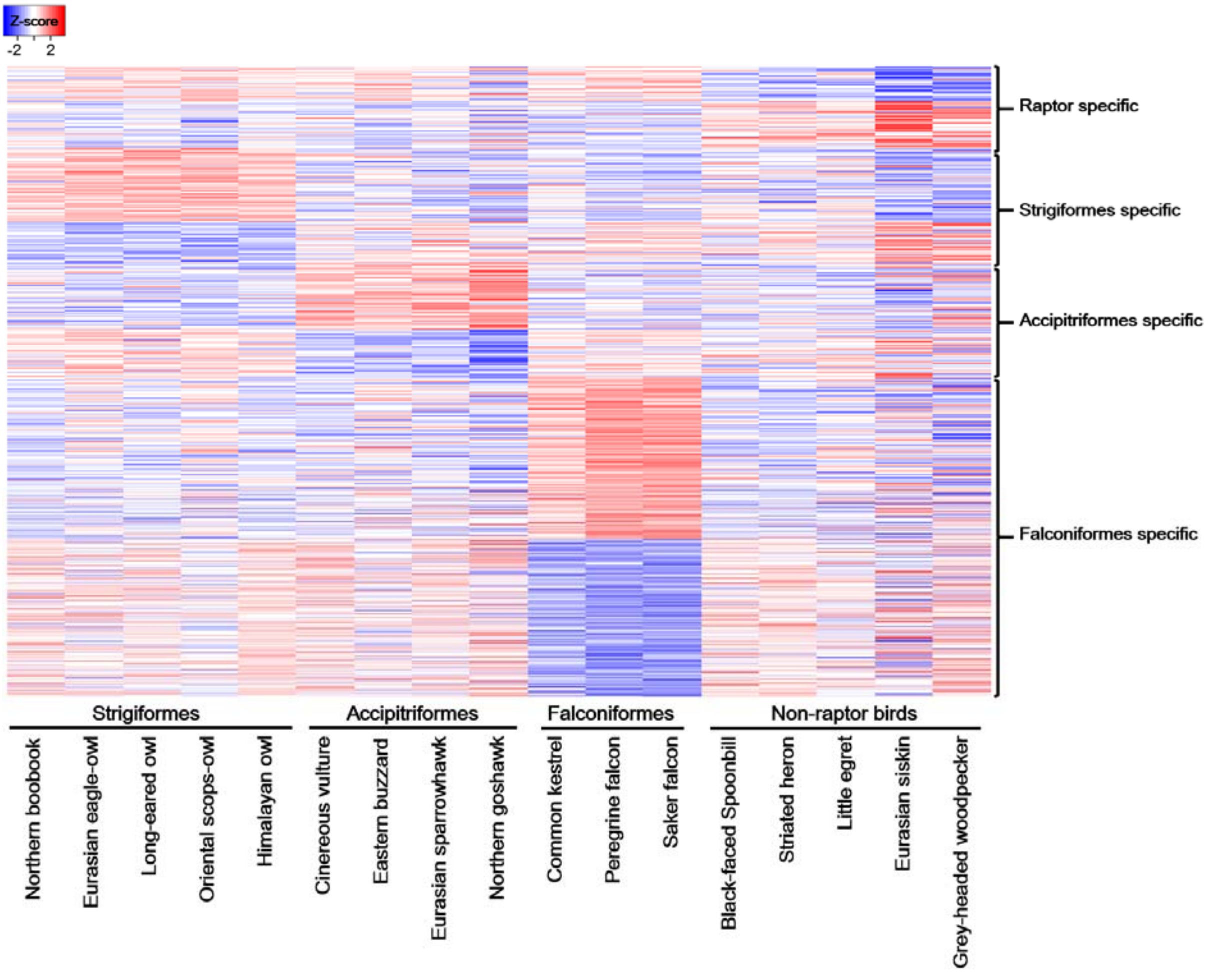

Figure S7. Differentially expressed genes (DEGs) in the birds of prey species. *P*-value ($P < 0.05$) heatmap of differentially expressed genes in the blood transcriptome of three raptor orders (Strigiformes, Accipitriformes, and Falconiformes). Greater and less than 2-fold expressions are shown in each column. The orders of DEGs were sorted by *P*-values in each target group (raptor-specific, Strigiformes-specific, Accipitriformes-specific, and Falconiformes-specific). The blood transcriptomes of black-faced spoonbill, striated heron, little egret, Eurasian siskin, and grey-headed woodpecker were used as a control group (non-raptor birds).

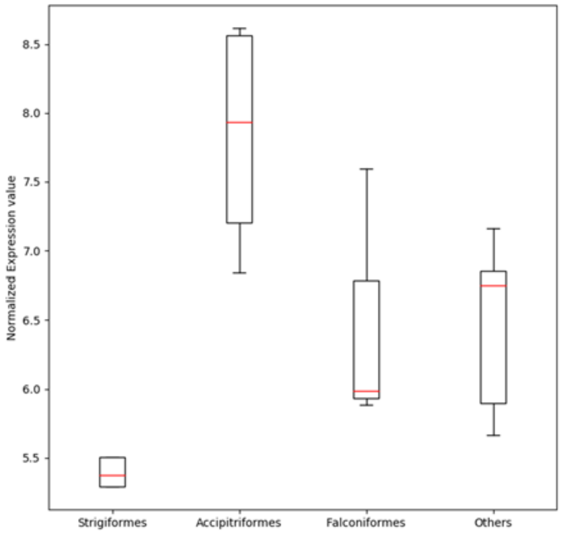

PDCL gene

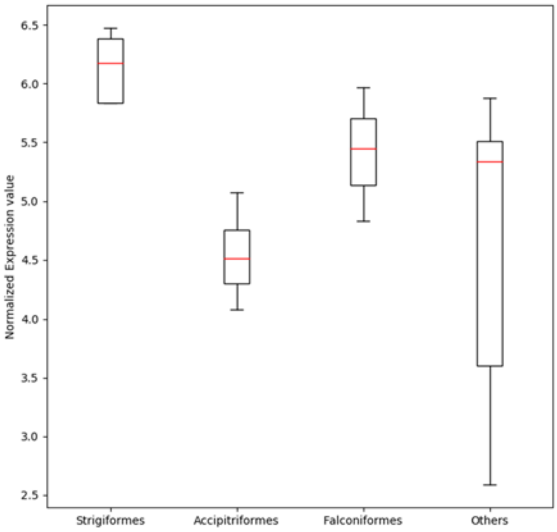

WFS1 gene

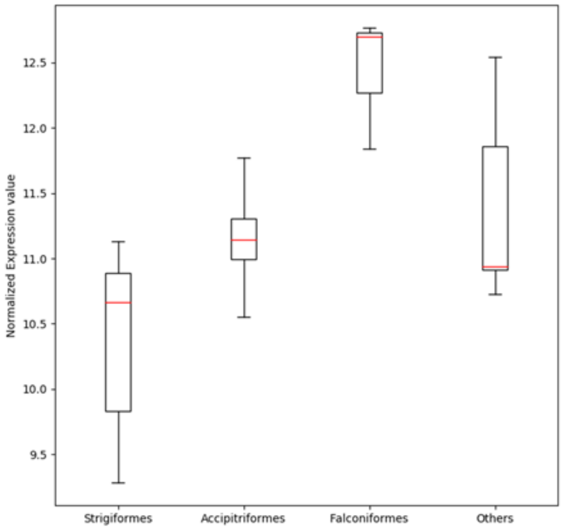

ATF4 gene

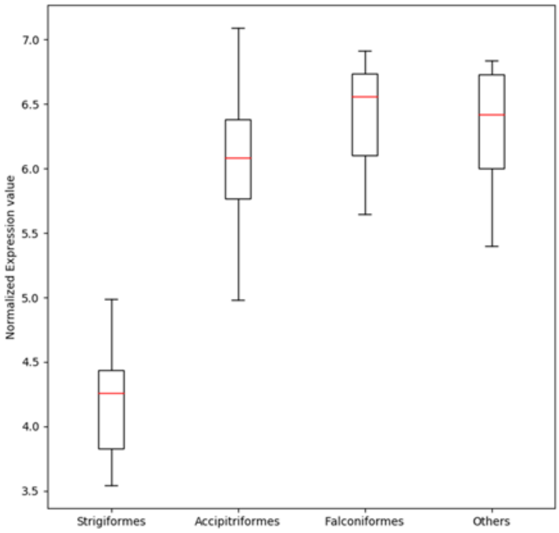

PER3 gene

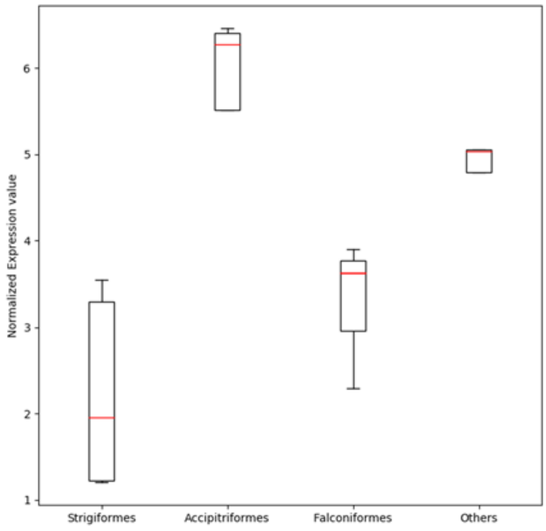

NRIP1 gene

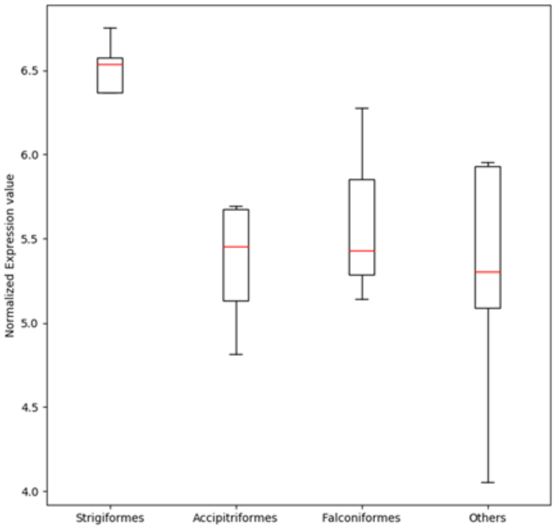

BTBD9 gene

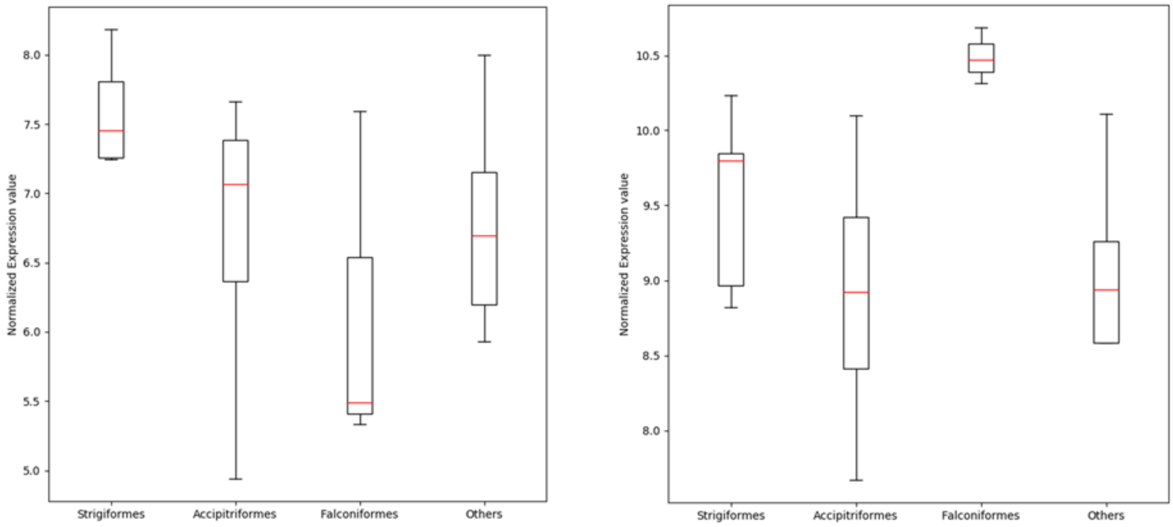

SETX gene *SIRT1* gene

Figure S8. Differentially expressed genes associated with the vision system and circadian rhythm. The normalized expression values were compared among Strigiformes (northern boobook, Eurasian eagle-owl, long-eared owl, oriental scops-owl, and Himalayan owl), Accipitriformes (cinereous vulture, eastern buzzard, Eurasian sparrowhawk, and northern goshawk), Falconiformes (common kestrel, peregrine falcon, and saker falcon), and non-raptor birds (black-faced spoonbill, striated heron, little egret, Eurasian siskin, and grey-headed woodpecker).

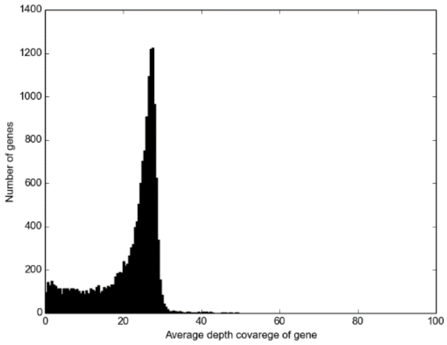

Eurasian eagle-owl

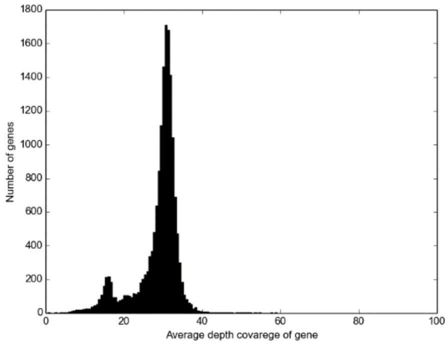

Northern spotted-owl

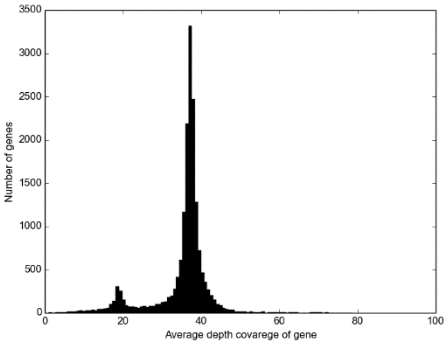

Oriental scops-owl

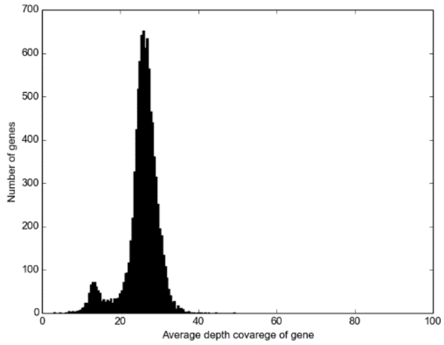

Barn owl

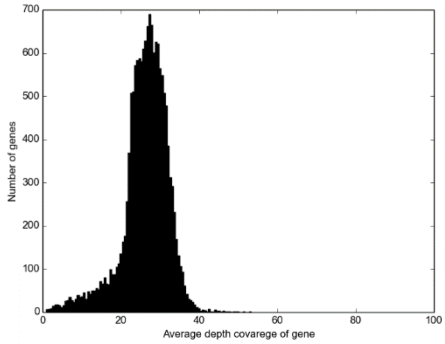

Golden eagle

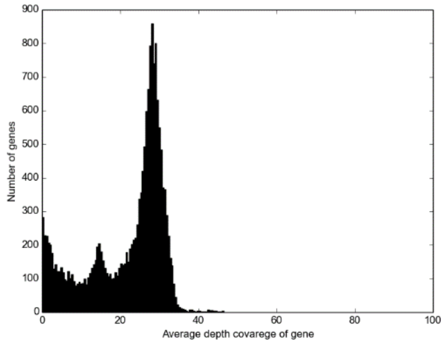

Eastern buzzard

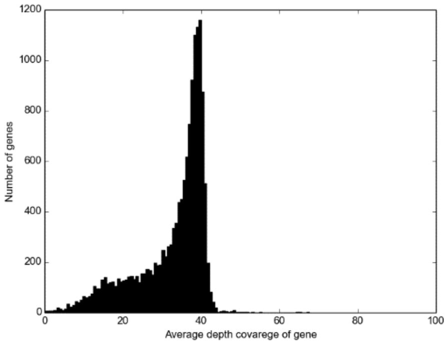

Bald eagle

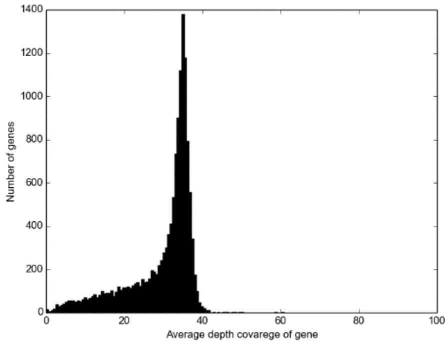

Saker falcon

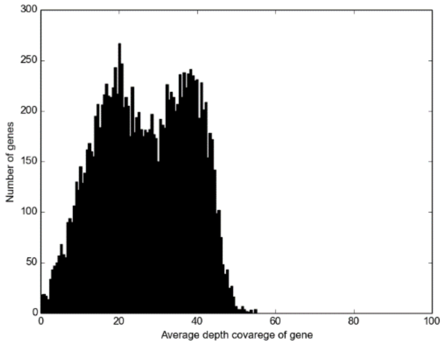

Peregrine falcon

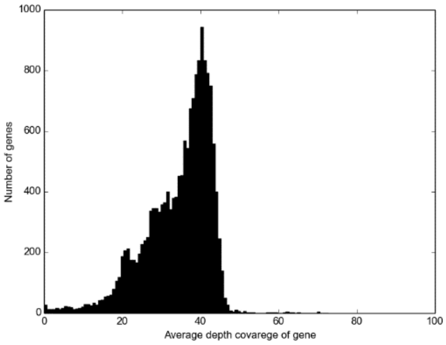

Common kestrel

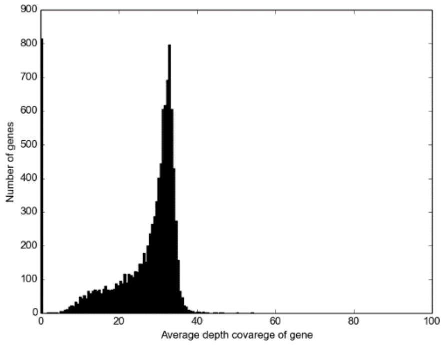

Chuck-will’s-widow

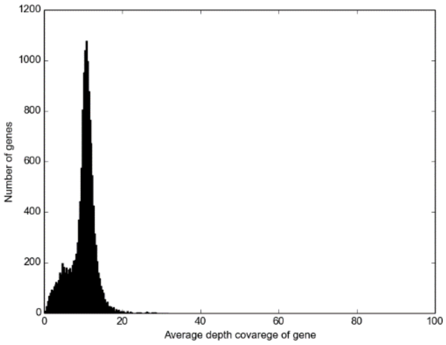

Common kestrel

Figure S9. The mapping depth coverage of genes in the birds of prey and nocturnal birds. A small peak was observed at the half of the average mapping depth in the most species, suggesting that there are erroneous genomic regions derived from the assembly process. Chuck-will’s-widow genome has many zero-coverage genes. Therefore, we filtered out the genes having abnormal depth coverage.

Supplementary Tables

Table S1. Bird of prey genome and transcriptome data used in this study

Order	Family	Species	Common name	Data	IUCN Red List	Source
Strigiformes	Strigidae	*Bubo bubo*	Eurasian eagle-owl	Assembly, Transcriptome	Least Concern	This study
		Otus sunia	Oriental scops-owl	Assembly, Transcriptome	Least Concern	This study
		Strix nivicolum	Himalayan owl	WGS, Transcriptome	Least Concern	This study
		Strix occidentalis	Northern spotted owl	Assembly	Near Threatened	S1
		Strix Varia	Barred owl	WGS	Least Concern	S1
		Ninox japonica	Northern boobook	WGS, Transcriptome	Least Concern	This study
		Asio otus	Long-eared owl	WGS, Transcriptome	Least Concern	This study
		Asio flammeus	Short-eared owl	WGS	Least Concern	This study
		Otus semitorques	Japanese scops-owl	WGS	Least Concern	This study
	Tytonidae	*Tyto alba*	Barn owl	Assembly (low quality)	Least Concern	S2
Accipitriformes	Accipitridae	*Buteo japonicus*	Eastern buzzard	Assembly, Transcriptome	Least Concern	This study
		Haliaeetus leucocephalus	Bald eagle	Assembly	Least Concern	S2
		Aquila chrysaetos	Golden eagle	Assembly	Least Concern	S3
		Accipiter nisus	Eurasian sparrowhawk	WGS, Transcriptome	Least Concern	This study
		Accipiter gentilis	Northern goshawk	WGS, Transcriptome	Least Concern	This study
		Aegypius monachus	Cinereous vulture	WGS, Transcriptome	Near Threatened	S4
		Haliaeetus albicilla	White-tailed eagle	WGS, low-quality assembly is available, but not used in this study	Least Concern	This study, S2
		Pernis ptilorhynchus	Oriental honey-buzzard	WGS	Least Concern	This study
		Milvus migrans	Black kite	WGS	Least Concern	This study
		Accipiter soloensis	Chinese sparrowhawk	WGS	Least Concern	This study
	Cathartidae	*Cathartes aura*	Turkey vulture	low-quality assembly is available, but not used in this study	Least Concern	S2
Falconiformes	Falconidae	*Falco tinnunculus*	Common kestrel	Assembly, WGS, Transcriptome	Least Concern	This study
		Falco cherrug	Saker falcon	Assembly, Transcriptome	Endangered	S5
		Falco peregrinus	Peregrine falcon	Assembly, Transcriptome	Least Concern	S5
		Falco subbuteo	Eurasian hobby	WGS, Transcriptome	Least Concern	This study

Table S2. Non-raptor bird genome and transcriptome data used for comparative evolutionary analysis

Order	Family	Species	Common name	Data	Source
Piciformes	Picidae	*Dryobates pubescens*	Downy woodpecker	Assembly	S2
	Picidae	*Picus canus*	Grey-headed woodpecker	WGS, Transcriptome	This study
Psittaciformes	Psittaculidae	*Melopsittacus undulatus*	Budgerigar	Assembly	S6
Passeriformes	Corvidae	*Corvus brachyrhynchos*	American crow	Assembly	S2
	Estrildidae	*Taeniopygia guttata*	Zebra finch	Assembly	S7
	Thraupidae	*Geospiza fortis*	Medium ground-finch	WGS; assemblies are available, but used as whole genome sequencing data for HCR analysis	S2
	Emberizidae	*Zonotrichia albicollis*	White-throated sparrow		SRR1796638, SRR1796642
	Fringillidae	*Serinus canaria*	Common canary		SRR2895902
	Muscicapidae	*Ficedula albicollis*	Collared flycatcher		ERR637368
	Fringillidae	*Spinus spinus*	Eurasian siskin	Transcriptome	S8
Pelecaniformes	Threskiornithidae	*Nipponia nippon*	Crested ibis	Assembly	S2
	Ardeidae	*Egretta garzetta*	Little egret	Assembly	S2
				WGS, Transcriptome	This study
	Ardeidae	*Butorides striata*	Striated heron	WGS, Transcriptome	This study
	Threskiornithidae	*Platalea minor*	Black-faced spoonbill	WGS, Transcriptome	This study
Opisthocomiformes	Opisthocomidae	*Opisthocomus hoazin*	Hoatzin	Assembly	S2
Charadriiformes	Charadriidae	*Charadrius vociferus*	Killdeer	Assembly	S2
Cuculiformes	Cuculidae	*Cuculus canorus*	Common cuckoo	Assembly	S2
Caprimulgiformes	Caprimulgidae	*Antrostomus carolinensis*	Chuck-will's-widow	Assembly (low quality)	S2
Apodiformes	Trochilidae	*Calypte anna*	Anna's hummingbird	Assembly	S2
Columbiformes	Columbidae	*Columba livia*	Rock dove	Assembly	S9
Galliformes	Phasianidae	*Gallus gallus*	Chicken	Assembly	S10
Struthioniformes	Struthionidae	*Struthio camelus*	Common ostrich	Assembly	S2
Apterygiformes	Apterygidae	*Apteryx australis*	Brown kiwi	Assembly	S11

Table S3. Sampling information on bird species sequenced in this study. All samples were acquired from South Korea. Genomic DNA of each sample was deposited at the Wildlife Genetic Resources Bank of National Institute of Biological Resources.

Scientific name	Collection number	Sampling date	Sample type[1]	Sampling site[2]	Accession number	Conservation Status[3]	Permission number[4]
**Strigiformes**							
Bubo bubo	CN14-281	Jun. 17, 2014	Blood	Dangjin, CN	NIBRGR0000424809	E(II), NM	CHA20150724
Otus sunia	CN15-715	Sep. 10, 2015	Blood	Cheonan, CN	NIBRGR0000424814		
Strix nivicolum	CN14-119	Jun. 17, 2014	Blood	Cheongyang, CN	NIBRGR0000424811	E(II), NM	CHA 20150724
Ninox japonica	CN14-387	Jun. 25, 2014	Blood	Dangjin, CN	NIBRGR0000424812	NM	CHA 20150724
Asio otus	CN14-021	Aug. 5, 2014	Blood	Asan, CN	NIBRGR0000424813	NM	CHA 20150724
Asio flammeus	IN1668	Feb. 6, 2017	Blood*	Cheongju, CB	NIBRGR0000424831	NM	Not necessary
Otus semitorques	CN17-040	Feb. 15, 2017	Blood*	Asan, CN	NIBRGR0000424811	NM	CHA 20170215
**Accipitriformes**							
Buteo japonicus	CN15-030	Jan. 26, 2015	Blood	Gwangju	NIBRGR0000424805		
Accipiter nisus	CN15-041	Feb. 5, 2015	Blood	Cheonan, CN	NIBRGR0000424806	E(II), NM	CHA 20150724
Accipiter gentilis	CN14-679	Nov. 13, 2014	Blood	Nonsan, CN	NIBRGR0000424819	E(II), NM	CHA 20150724
Haliaeetus albicilla	CN16-053	Feb. 7, 2017	Blood	Seosan, CN	NIBRGR0000424828	E(I), NM	MOE 2017-03
Pernis ptilorhynchus	CN11-616	Jan. 5, 2017	Blood*	Seosan, CN	NIBRGR0000424829	E(II)	MOE 2017-01
Milvus migrans	IN1594	Jun. 1, 2017	Muscle	Busan	NIBRGR0000424830	E(II)	MOE 2017-18
Accipiter soloensis	IN1803	Jun. 12, 2017	Muscle	Busan	NIBRGR0000424832	NM	CHA 20170608
**Falconiformes**							
Falco tinnunculus	CN14-642	Nov. 24, 2014	Blood	Seosan, CN	NIBRGR0000424802	NM	CHA 20150724
Falco subbuteo	CN14-472	Jul. 19, 2014	Blood	Seocheon, CN	NIBRGR0000424810	E(II)	MOE 2017-34
**Others**							
Picus canus	CN14-463	Jul. 18, 2014	Blood	Cheonan, CN	NIBRGR0000424815		
Egretta garzetta	CN14-545	Aug. 14, 2014	Blood	Nonsan, CN	NIBRGR0000424817		
Butorides striata	CN14-540	Aug. 14, 2014	Blood	Yesan, CN	NIBRGR0000424816		
Platalea minor	CN14-386	Jun. 25, 2014	Blood	Incheon	NIBRGR0000424818	E(I), NM	CHA 20150724

1. Blood samples for *A. flammeus, O. semitorques*, and *P. ptilorhynchus* (denoted with *) were obtained from the live individuals during medical check-up at the wild animal rescue center or at the zoo.
2. Abbreviation for sampling sites are: CN, Chungcheongnam-do and CB, Chungcheongbuk-do
3. Conservation status: Endangered species of Korea (E) in tier I or tier II were listed by Ministry of Environment (MOE). Natural Monument (NM) were listed by Cultural Heritage Administration (CHA).
4. Permissions for sampling were obtained from MOE for the endangered species of Korea and from the CHA for the natural monuments, respectively. Permission for *A. flammeus*, a natural monument, was not necessary because blood samples were obtained during medical check-up at the zoo.

Table S4. Sequencing library statistics used for the four bird of prey genome assemblies

a. Eurasian eagle-owl

Insert size	Libraries	Number of reads	Read length (bp)	Total bases (bp)	Depth (×) (Genome size: 1.2 Gb)
170bp	L1	498,582,512	101	50,356,833,712	41.96
500bp	L1	426,406,536	101	43,067,060,136	35.89
700bp	L1	481,281,336	101	48,609,414,936	40.51
2 Kb	L1	288,749,880	51	14,726,243,880	12.27
	L2	290,046,702	51	14,792,381,802	12.33
5 Kb	L1	336,003,522	51	17,136,179,622	14.28
	L2	252,776,316	51	12,891,592,116	10.74
10 Kb	L1	271,847,720	51	13,864,233,720	11.55
	L2	274,540,178	51	14,001,549,078	11.67
15 Kb	L1	273,358,018	51	13,941,258,918	11.62
	L2	263,444,524	51	13,435,670,724	11.20
Total		3,657,037,244	-	256,822,418,644	214.02

b. Oriental scops-owl

Insert size	Libraries	Number of reads	Read length (bp)	Total bases (bp)	Depth (×) (Genome size: 1.2 Gb)
170bp	L1	361,705,544	101	36,532,259,944	30.44
500bp	L1	509,968,954	101	51,506,864,354	42.92
700bp	L1	592,847,314	101	59,877,578,714	49.90
2 Kb	L1	250,487,712	51	12,774,873,312	10.65
	L2	236,796,524	51	12,076,622,724	10.06
5 Kb	L1	226,395,658	51	11,546,178,558	9.62
	L2	283,856,328	51	14,476,672,728	12.06
10 Kb	L1	290,050,882	51	14,792,594,982	12.33
	L2	316,864,714	51	16,160,100,414	13.47
15 Kb	L1	251,155,552	51	12,808,933,152	10.67
	L2	279,791,232	51	14,269,352,832	11.89
Total		3,599,920,414	-	256,822,031,714	214.02

c. Eastern buzzard

Insert size	Libraries	Number of reads	Read length (bp)	Total bases (bp)	Depth (×) (Genome size: 1.2 Gb)
170bp	L1	370,648,156	101	37,435,463,756	31.20
500bp	L1	383,420,678	101	38,725,488,478	32.27
700bp	L1	392,938,934	101	39,686,832,334	33.07
2 Kb	L1	264,343,284	51	13,481,507,484	11.23
	L2	228,311,168	51	11,643,869,568	9.70
5 Kb	L1	284,204,000	51	14,494,404,000	12.08
	L2	213,496,276	51	10,888,310,076	9.07
10 Kb	L1	241,959,836	51	12,339,951,636	10.28
	L2	287,893,998	51	14,682,593,898	12.24
15 Kb	L1	256,954,832	51	13,104,696,432	10.92
	L2	314,295,666	51	16,029,078,966	13.36
Total		3,238,466,828	-	222,512,196,628	185.43

d. Common kestrel

Insert size	Libraries	Number of reads	Read length (bp)	Total bases (bp)	Depth (×) (Genome size: 1.2 Gb)
350bp	L1	512,005,734	101	51,712,579,134	43.09
550bp	L2	531,996,158	101	53,731,611,958	44.78
2 Kb	L1	505,177,478	101	51,022,925,278	42.52
	L2	515,046,928	101	52,019,739,728	43.35
	L3	471,276,708	101	47,598,947,508	39.67
5 Kb	L1	532,540,204	101	53,786,560,604	44.82
	L2	541,427,728	101	54,684,200,528	45.57
10 Kb	L1	528,032,494	101	53,331,281,894	44.44
	L2	573,922,840	101	57,966,206,840	48.31
15 Kb	L1	582,372,866	101	58,819,659,466	49.02
	L2	580,606,468	101	58,641,253,268	48.87
Total		5,874,405,606	-	593,314,966,206	494.43

Table S5. Filtered sequence information of the four birds of prey

a. Eurasian eagle-owl

Libraries		Number of raw reads	Number of remained reads	Trimmed read length (bp)	Remained total bases (bp)	Remained sequence depth (×)
170bp	L1	498,582,512	473,524,892	90	42,617,240,280	35.51
500bp	L1	426,406,536	392,021,480	90	35,281,933,200	29.40
700bp	L1	481,281,336	393,095,546	90	35,378,599,140	29.48
2 Kb	L1	288,749,880	29,168,546	50	1,458,427,300	1.22
	L2	290,046,702	40,732,148	50	2,036,607,400	1.70
5 Kb	L1	336,003,522	38,862,934	50	1,943,146,700	1.62
	L2	252,776,316	28,531,004	50	1,426,550,200	1.19
10 Kb	L1	271,847,720	23,306,048	50	1,165,302,400	0.97
	L2	274,540,178	22,215,842	50	1,110,792,100	0.93
15 Kb	L1	273,358,018	22,029,430	50	1,101,471,500	0.92
	L2	263,444,524	22,083,214	50	1,104,160,700	0.92
Total		3,657,037,244	1,485,571,084	-	124,624,230,920	103.85

b. Oriental scops-owl

Libraries		Number of raw reads	Number of remained reads	Trimmed read length (bp)	Remained total bases (bp)	Remained sequence depth (×)
170bp	L1	361,705,544	350,574,648	90	31,551,718,320	26.29
500bp	L1	509,968,954	462,578,698	90	41,632,082,820	34.69
700bp	L1	592,847,314	563,845,942	90	50,746,134,780	42.29
2 Kb	L1	250,487,712	67,353,140	50	3,367,657,000	2.81
	L2	236,796,524	57,084,566	50	2,854,228,300	2.38
5 Kb	L1	226,395,658	32,235,478	50	1,611,773,900	1.34
	L2	283,856,328	37,503,362	50	1,875,168,100	1.56
10 Kb	L1	290,050,882	22,144,478	50	1,107,223,900	0.92
	L2	316,864,714	26,887,942	50	1,344,397,100	1.12
15 Kb	L1	251,155,552	16,405,590	50	820,279,500	0.68
	L2	279,791,232	21,354,764	50	1,067,738,200	0.89
Total		3,599,920,414	1,657,968,608	-	137,978,401,920	114.98

c. Eastern buzzard

Libraries		Number of raw reads	Number of remained reads	Trimmed read length (bp)	Remained total bases (bp)	Remained sequence depth (×)
170bp	L1	370,648,156	357,178,300	90	32,146,047,000	26.79
500bp	L1	383,420,678	356,087,914	90	32,047,912,260	26.71
700bp	L1	392,938,934	329,183,348	90	29,626,501,320	24.69
2 Kb	L1	264,343,284	42,693,738	50	2,134,686,900	1.78
	L2	228,311,168	84,073,436	50	4,203,671,800	3.50
5 Kb	L1	284,204,000	43,430,604	50	2,171,530,200	1.81
	L2	213,496,276	23,605,016	50	1,180,250,800	0.98
10 Kb	L1	241,959,836	23,038,434	50	1,151,921,700	0.96
	L2	287,893,998	27,225,910	50	1,361,295,500	1.13
15 Kb	L1	256,954,832	18,981,222	50	949,061,100	0.79
	L2	314,295,666	26,171,024	50	1,308,551,200	1.09
Total		3,238,466,828	1,331,668,946	-	108,281,429,780	90.23

d. Common kestrel

Libraries		Number of raw reads	Number of remained reads	Trimmed read length (bp)	Remained total bases (bp)	Remained sequence depth (×)
350bp	L1	370,648,156	357,178,300	90	32,146,047,000	26.79
550bp	L1	383,420,678	356,087,914	90	32,047,912,260	26.71
2 Kb	L1	392,938,934	329,183,348	90	29,626,501,320	24.69
	L1	264,343,284	42,693,738	50	2,134,686,900	1.78
	L2	228,311,168	84,073,436	50	4,203,671,800	3.50
5 Kb	L1	284,204,000	43,430,604	50	2,171,530,200	1.81
	L2	213,496,276	23,605,016	50	1,180,250,800	0.98
10 Kb	L1	241,959,836	23,038,434	50	1,151,921,700	0.96
	L2	287,893,998	27,225,910	50	1,361,295,500	1.13
15 Kb	L1	256,954,832	18,981,222	50	949,061,100	0.79
	L2	314,295,666	26,171,024	50	1,308,551,200	1.09
Total		5,874,405,606	2,737,928,632	-	178,015,653,600	148.35

Table S6. 17-mer statistics information for 20 avian species

Species	*K*-mer total number	Peak depth	Estimated genome size (Gb)
Eurasian eagle-owl	93,139,501,932	63	1.478
Oriental scops-owl	101,897,947,312	77	1.323
Himalayan owl	25,301,442,982	20	1.265
Northern boobook	25,148,256,432	20	1.257
Long-eared owl	29,586,880,640	24	1.233
Short-eared owl	23,353,960,560	14	1.668
Japanese scops-owl	27,992,952,456	22	1.272
Eastern buzzard	77,141,267,588	63	1.224
Eurasian sparrowhawk	29,072,816,384	21	1.384
Northern goshawk	30,522,477,568	21	1.453
White-tailed eagle	24,390,282,000	18	1.355
Oriental honey-buzzard	25,305,632,016	16	1.582
Black kite	25,712,659,392	19	1.353
Chinese sparrowhawk	22,366,586,088	15	1.491
Common kestrel	76,070,560,700	60	1.268
Eurasian hobby	30,173,856,256	23	1.312
Grey-headed woodpecker	13,036,606,884	10	1.304
Little Egret	11,702,977,902	10	1.170
Striated heron	11,750,011,056	10	1.175
Black-faced spoonbill	12,485,284,056	11	1.135

Table S7. Global assembly statistics of the four bird of prey genomes. Final assemblies were selected by assessing assembly statistics, transcripts mapping results, and single-copy orthologs mapping results. Oriental scops-owl genome could not be assembled when using SOAPdenovo2, probably because of its high level of heterozygosity.

a. Eurasian eagle-owl

Assembler	**SOAPdenovo2 (selected)**		Platanus	
Assembly level	Contig	Scaffold	Contig	Scaffold
# of sequences	133,374	35,672	119,288	72,452
Total bases (bp)	1,201,498,232	1,258,075,470	1,168,795,702	1,194,726,138
Longest length (bp)	391,237	29,915,079	374,073	33,945,943
Shortest length (bp)	55	200	1	200
N50 (bp)	37,088	8,019,613	46,324	6,858,015
GC contents	41.39 %	41.39 %	41.10 %	41.10 %
N base ratio	0.00 %	4.50 %	0.00 %	2.17 %

b. Oriental scops-owl

Assembler	**Platanus (selected)**	
Assembly level	Contig	Scaffold
# of sequences	151,529	108,835
Total bases (bp)	1,212,043,918	1,231,452,760
Longest length (bp)	406,680	81,210,816
Shortest length (bp)	2	200
N50 (bp)	52,366	18,540,887
GC contents	41.70 %	41.70 %
N base ratio	0.00 %	1.58 %

c. Eastern buzzard

Assembler	**SOAPdenovo2 (selected)**		Platanus	
Assembly level	Contig	Scaffold	Contig	Scaffold
# of sequences	127,106	29,208	112,214	65,890
Total bases (bp)	1,216,111,145	1,259,752,395	1,178,354,735	1,203,691,678
Longest length (bp)	370,481	31,377,212	389,759	32,375,696
Shortest length (bp)	55	200	5	200
N50 (bp)	36,722	7,468,869	46,157	8,836,483
GC contents	41.66 %	41.66 %	41.38 %	41.38 %
N base ratio	0.00 %	3.46 %	0.00 %	2.11 %

d. Common kestrel

Assembler	SOAPdenovo2		**Platanus (selected)**	
Assembly level	Contig	Scaffold	Contig	Scaffold
# of sequences	245,216	34,131	123,820	69,121
Total bases (bp)	1,342,122,283	1,430,330,502	1,173,072,102	1,179,916,286
Longest length (bp)	138,002	44,828,457	305,150	64,290,118
Shortest length (bp)	1	199	1	200
N50 (bp)	12,031	13,066,975	37,053	21,232,185
GC contents	42.20 %	42.20 %	42.11 %	42.11 %
N base ratio	0.00 %	6.17 %	0.00 %	0.58 %

Table S8. Assessment of gene coverage by assembled bird of prey transcripts. Final assemblies were selected by assessing assembly statistics, transcripts mapping results, and single-copy ortholog mapping results.

Species	Assembler	Transcript length	Number	Total length (bp)	Sequence covered by Assembly (%)	With >90% sequence in one scaffold		With >50% sequence in one scaffold	
						Number	Percent (%)	Number	Percent (%)
Eurasian eagle-owl	**SOAPdenovo2 (selected)**	All	192,557	247,620,874	94.20	164,428	85.39	175,583	91.18
		>200bp	192,557	247,620,874	94.20	164,428	85.39	175,583	91.18
		>500bp	98,755	216,127,215	95.15	85,725	86.81	93,076	94.25
		>1000bp	58,927	188,526,444	95.84	51,799	87.90	56,745	96.30
	Platanus	All	192,557	247,620,874	89.46	145,471	75.55	164,060	85.20
		>200bp	192,557	247,620,874	89.46	145,471	75.55	164,060	85.20
		>500bp	98,755	216,127,215	90.79	76,580	77.55	88,193	89.30
		>1000bp	58,927	188,526,444	91.68	46,400	78.74	53,814	91.32
Oriental scops-owl	**Platanus (selected)**	All	188,436	226,146,141	96.19	164,344	87.21	178,179	94.56
		>200bp	188,436	226,146,141	96.19	164,344	87.21	178,179	94.56
		>500bp	94,884	195,260,793	97.03	85,930	90.56	82,093	97.06
		>1000bp	54,707	167,470,729	97.52	50,922	93.08	53,697	98.15
Eastern buzzard	**SOAPdenovo2 (selected)**	All	177,945	231,089,047	97.84	166,819	93.75	175,078	98.39
		>200bp	177,945	231,089,047	97.84	166,819	93.75	175,078	98.39
		>500bp	95,936	203,493,171	97.96	89,978	93.79	94,883	98.90
		>1000bp	56,968	176,443,974	98.07	53,696	94.26	56,545	99.26
	Platanus	All	177,945	231,089,047	94.28	150,231	84.43	166,059	93.32
		>200bp	177,945	231,089,047	94.28	150,231	84.43	166,059	93.32
		>500bp	95,936	203,493,171	94.82	81,824	85.29	91,211	95.07
		>1000bp	56,968	176,443,974	95.21	49,053	86.11	54,562	95.78
Common kestrel	SOAPdenovo2	All	181,982	226,109,028	99.26	177,597	97.59	181,297	99.62
		>200bp	181,982	226,109,028	99.26	177,597	97.59	181,297	99.62
		>500bp	97,087	197,379,462	99.32	95,323	98.18	96,839	99.74
		>1000bp	57,126	169,661,898	99.36	56,200	98.38	57,023	99.82
	**Platanus (selected)**	All	181,982	226,109,028	97.70	171,187	94.07	178,926	98.32
		>200bp	181,982	226,109,028	97.70	171,187	94.07	178,926	98.32
		>500bp	97,087	197,379,462	97.73	91,572	94.32	95,139	97.99
		>1000bp	57,126	169,661,898	97.85	54,237	94.94	56,027	98.08

Table S9. Protein-coding gene prediction statistics for the four birds of prey. Final assemblies were selected by assessing assembly statistics, transcripts mapping results, and single-copy ortholog mapping results. For the common kestrel, only the Platanus assembly was tested, since the N50 lengths of contig and scaffold were much longer than those of SOAPdenovo2 assembly.

a. Eurasian eagle-owl

Assembler	Gene set		Number	Average transcript length (bp)	Average CDS length (bp)	Average no. of exons per gene	Average exon length (bp)	Average intron length (bp)
**SOAPdenovo2 (selected)**	*de novo*	AUGUSTUS	27,448	19,208.9	1,390.8	7.8	178.5	2,624.1
	Homolog	Bald eagle	13,546	23,302.6	1,598.9	9.3	171.4	2,605.4
		Barn owl	14,897	15,380.9	1,270.6	7.6	167.5	2,141.8
		Brown kiwi	14,218	20,732.4	1,434.5	8.5	169.5	2,585.9
		Chicken	14,105	22,318.1	1,512.0	8.9	170.6	2,646.5
		Common cuckoo	13,366	22,770.5	1,545.2	9.1	169.0	2,607.3
		Crested ibis	13,173	22,868.5	1,539.9	9.2	166.9	2,592.2
		Downy woodpecker	13,054	22,747.4	1,510.0	9.0	168.2	2,662.2
		Golden eagle	13,939	22,994.4	1,598.0	9.3	171.6	2,574.6
		Peregrine falcon	13,059	23,252.2	1,566.2	9.3	168.6	2,616.4
		Zebra finch	13,941	20,970.8	1,427.0	8.3	171.2	2,664.7
	Final		16,897	18,578.1	1,335.5	8.1	164.6	2,569.9
Platanus	*de novo*	AUGUSTUS	25,271	19,055.9	1,383.4	7.7	180.0	2,644.0
	Homolog	Bald eagle	12,218	24,168.0	1,649.1	9.6	170.9	2,604.7
		Barn owl	14,022	15,714.5	1,308.1	7.8	168.5	2,131.0
		Brown kiwi	13,417	20,572.0	1,441.8	8.5	170.5	2,567.3
		Chicken	13,035	22,596.6	1,535.2	9.0	171.3	2,645.9
		Common cuckoo	12,540	22,774.5	1,576.6	9.1	172.9	2,612.6
		Crested ibis	11,905	23,774.4	1,590.6	9.6	166.4	2,592.8
		Downy woodpecker	12,208	22,725.9	1,544.4	9.0	171.5	2,646.7
		Golden eagle	12,823	23,428.3	1,641.3	9.5	173.1	2,569.2
		Peregrine falcon	11,849	23,852.6	1,603.9	9.6	167.7	2,598.6
		Zebra finch	12,565	21,855.6	1,467.6	8.7	169.5	2,663.3
	Final		15,632	18,885.8	1,350.2	8.2	164.4	2,431.9

b. Oriental scops-owl

Assembler	Gene set		Number	Average transcript length (bp)	Average CDS length (bp)	Average no. of exons per gene	Average exon length (bp)	Average intron length (bp)
**Platanus (selected)**	*de novo*	AUGUSTUS	26,719	18,400.4	1,353.3	8.0	168.6	2,427.4
	Homolog	Bald eagle	13,736	23,055.6	1,651.1	9.7	170.3	2,462.1
		Barn owl	14,707	15,171.4	1,280.4	7.6	168.1	2,099.9
		Brown kiwi	14,492	19,803.5	1,435.5	8.5	169.9	2,466.3
		Chicken	14,445	22,156.5	1,561.3	9.2	170.4	2,523.4
		Common cuckoo	13,791	21,929.5	1,569.9	9.2	169.9	2,471.5
		Crested ibis	13,397	22,349.9	1,584.0	9.5	167.5	2,455.3
		Downy woodpecker	13,270	22,134.3	1,547.6	9.2	168.3	2,511.5
		Golden eagle	14,259	22,772.8	1,656.2	9.7	171.4	2,437.9
		Peregrine falcon	13,298	22,601.0	1,607.8	9.6	168.4	2,456.2
		Zebra finch	13,756	21,011.2	1,459.0	8.6	168.9	2,559.8
	Final		17,710	17,497.8	1,349.1	8.2	165.2	2,402.5

c. Eastern buzzard

Assembler	Gene set		Number	Average transcript length (bp)	Average CDS length (bp)	Average no. of exons per gene	Average exon length (bp)	Average intron length (bp)
**SOAPdenovo2 (selected)**	*de novo*	AUGUSTUS	23,535	21,045.9	1,515.8	9.1	167.4	2,425.9
	Homolog	Bald eagle	14,928	22,831.0	1,613.9	9.5	170.6	2,508.0
		Barn owl	15,423	15,000.1	1,247.6	7.5	167.0	2,125.6
		Brown kiwi	13,699	22,816.1	1,540.9	9.1	168.8	2,617.6
		Chicken	14,635	21,995.1	1,526.5	9.0	169.4	2,554.9
		Common cuckoo	13,680	22,806.9	1,558.7	9.4	166.6	2,542.6
		Crested ibis	13,605	22,829.6	1,557.0	9.4	166.2	2,541.1
		Downy woodpecker	13,321	22,874.1	1,524.1	9.2	165.9	2,607.0
		Golden eagle	15,039	22,920.9	1,625.7	9.6	169.9	2,485.0
		Peregrine falcon	13,778	22,611.3	1,565.7	9.3	168.6	2,539.8
		Zebra finch	14,209	20,810.9	1,433.1	8.4	170.2	2,610.7
	Final		16,897	18,578.1	1,335.5	8.1	164.6	2,569.9
Platanus	*de novo*	AUGUSTUS	22,397	20,736.4	1,452.6	8.8	165.0	2,471.6
	Homolog	Bald eagle	13,318	23,831.3	1,660.3	9.8	169.1	2,514.6
		Barn owl	14,043	15,811.9	1,309.7	7.9	166.6	2,114.4
		Brown kiwi	12,501	23,252.4	1,571.7	9.4	167.8	2,592.2
		Chicken	13,009	22,973.1	1,581.5	9.4	168.1	2,544.3
		Common cuckoo	12,521	23,183.7	1,589.4	9.5	166.8	2,532.2
		Crested ibis	12,508	23,228.9	1,582.8	9.5	166.1	2,538.4
		Downy woodpecker	12,300	23,138.3	1,549.4	9.3	166.0	2,591.2
		Golden eagle	13,524	23,740.5	1,668.9	9.9	168.9	2,486.3
		Peregrine falcon	12,445	23,228.2	1,599.8	9.5	167.6	2,531.6
		Zebra finch	12,794	21,484.2	1,467.5	8.7	168.2	2,592.6
	Final		16,047	21,996.9	1,511.8	9.2	165.2	2,514.4

d. Common kestrel

Assembler	Gene set		Number	Average transcript length (bp)	Average CDS length (bp)	Average no. of exons per gene	Average exon length (bp)	Average intron length (bp)
**Platanus (selected)**	*de novo*	AUGUSTUS	20,277	23,031.3	1,564.6	9.8	160.5	2,454.0
	Homolog	Bald eagle	13,339	23,913.7	1,688.2	10.0	168.9	2,470.4
		Barn owl	14,132	15,516.0	1,298.8	7.8	165.9	2,081.5
		Brown kiwi	13,084	23,564.1	1,594.0	9.5	167.9	2,586.7
		Chicken	13,521	24,248.2	1,653.8	9.8	168.8	2,568.9
		Common cuckoo	13,200	22,945.3	1,610.3	9.7	166.4	2,458.1
		Crested ibis	13,035	22,877.4	1,605.6	9.6	167.1	2,470.5
		Downy woodpecker	12,779	23,058.3	1,573.3	9.5	165.4	2,524.4
		Golden eagle	13,598	23,869.9	1,690.9	10.0	168.4	2,453.7
		Peregrine falcon	13,838	23,504.8	1,652.8	9.9	166.5	2,447.3
		Zebra finch	13,088	22,327.8	1,511.0	9.1	166.4	2,576.5
	Final		16,481	22,122.1	1,553.8	9.4	164.6	2,584.6

Table S10. Evaluation of the completeness of bird of prey assemblies and gene sets using single-copy orthologs mapping approach. Final assemblies were selected by assessing assembly statistics, transcripts mapping results, and single-copy ortholog mapping results. For the common kestrel, only the Platanus assembly was tested, since the N50 lengths of contig and scaffold were much longer than those of SOAPdenovo2 assembly.

Species	Assembler	Number of predicted genes	Complete (%)	Duplicated (%)	Fragment (%)	Missing (%)	Number of single-copy orthologs genes
Eurasian eagle-owl	**SOAPdenovo2 (selected)**	16,897	92.09%	1.02%	5.96%	1.95%	4,915
	Platanus	15,632	91.27%	1.00%	6.57%	2.16%	4,915
Oriental scops-owl	**Platanus (selected)**	17,710	91.35%	1.44%	6.37%	2.28%	4,915
Eastern buzzard	**SOAPdenovo2 (selected)**	17,376	96.72%	1.18%	2.38%	0.90%	4,915
	Platanus	16,047	96.38%	1.04%	2.67%	0.96%	4,915
Common kestrel	**Platanus (selected)**	16,481	93.90%	1.22%	4.46%	1.65%	4,915

Table S11. Genome and transcriptome data and assembly quality information of avian species used in this study

a. High-quality (scaffold N50 length of > 1 Mb) genome assembly

Species	RefSeq accession	Total Sequence Length	Number of contigs	Contig N50	Number of scaffolds	Scaffold N50	Genome Coverage
Eurasian eagle-owl	This study	1.27 Gb	133,374	371 Kb	35,672	8.02 Mb	104×
Northern spotted owl	GCA_002372975.1	1.26 Gb	28,713	159.1 Kb	8,108	3.98 Mb	59×
Oriental scops-owl	This study	1,23 Gb	151,529	52.4 Kb	108,835	18.54 Mb	115×
Downy woodpecker	GCA_000699005.1	1.17 Gb	31,254	24.8 Kb	31,254	2.09 Mb	105×
Golden eagle	GCA_000766835.1	1.19 Gb	17,032	172.3 Kb	1,142	9.23 Mb	88×
Eastern buzzard	This study	1.26 Gb	127,106	36.7 Kb	29,208	7.47 Mb	90×
Bald eagle	GCA_000737465.1	1.18 Gb	31,786	105.5 Kb	1,023	9.15 Mb	103×
Saker falcon	GCA_000337975.1	1.17 Gb	75,898	31.3 Kb	5,863	4.15 Mb	147×
Peregrine falcon	GCA_000337955.1	1.17 Gb	83,081	28.6 Kb	7,021	3.94 Mb	137×
Common kestrel	This study	1.18 Gb	123,820	37.1 Kb	69,121	21.23 Mb	148×
Zebra Finch	GCA_000151805.2	1.23 Gb	124,806	38.6 Kb	37,422	8.24 Mb	6×
American crow	GCA_000691975.1	1.09 Gb	89,646	29.1 Kb	10,547	6.95 Mb	80×
Budgerigar	GCA_000238935.1	1.12 Gb	70,863	55.6 Kb	25,212	10.61 Mb	23×
Little egret	GCA_000687185.1	1.21 Gb	100,662	29.0 Kb	11,791	3.07 Mb	74×
Crested Ibis	GCA_000708225.1	1.22 Gb	130,030	29.1 Kb	59,555	5.21 Mb	105×
Hoatzin	GCA_000692075.1	1.20 Gb	109,627	28.2 Kb	10,256	2.94 Mb	100×
Killdeer	GCA_000708025.2	1.22 Gb	79,162	39.3 Kb	15,167	3.66 Mb	100×
Anna’s hummingbird	GCA_000699085.1	1.11 Gb	124,820	26.7 Kb	54,736	4.05 Mb	110×
Common cuckoo	GCA_000709325.1	1.15 Gb	71,907	38.1 Kb	14,930	2.99 Mb	100×
Rock dove	GCA_000337935.1 (	1.11 Gb	100,099	26.6 Kb	14,923	3.15 Mb	60×
Chicken	GCA_000002315.3	1.23 Gb	24,693	2.89 Mb	23,870	6.38 Mb	70×
Brown kiwi	GCA_001039765.2	1.60 Gb	514,807	16.2 Kb	59	3.96 Mb	37×
Common ostrich	GCA_000698965.1	1.23 Gb	79,564	35.0 Kb	6,915	3.59 Mb	85×

b. Low-quality (scaffold N50 length of < 1 Mb) genome assembly

Species	RefSeq accession	Total Sequence Length	Number of contigs	Contig N50	Number of scaffolds	Scaffold N50	Genome Coverage
Barn Owl	GCA_000687205.1	1.12 Gb	171,345	17.2 Kb	62,122	52.8 Kb	27×
Chuck-will’s-widow	GCA_000700745.1	1.12 Gb	126,789	22.2 Kb	70,122	46.3 Kb	30×

c. Whole genome sequence (WGS); not *de novo* assembled

Common name	Accession	Platform	Number of reads	Read length (bp)	Total bases (bp)	Depth (×) (Genome size: 1.2 Gb)
Himalayan owl	SRR6650820*	HiSeq 2000	251,231,134	100	25,123,113,400	39.78
	SRR6650821*	HiSeq 2500	223,889,662	101	22,612,855,862	
Northern spotted owl	SRR4011597	HiSeq 2500	342,525,114	150	51,378,767,100	42.82
Barred owl	SRR5428115	HiSeq 2500	84,166,646	150	12,624,996,900	31.79
	SRR5428116	HiSeq 2500	84,736,426	150	12,710,463,900	
	SRR5428117	HiSeq 2500	85,390,244	150	12,808,536,600	
Northern boobook	SRR6650819*	HiSeq 2000	264,137,496	100	26,413,749,600	40.44
	SRR6650816*	HiSeq 2500	218,933,136	101	22,112,246,736	
Long-eared owl	SRR6650824*	HiSeq 2000	255,516,898	100	25,551,689,800	39.72
	SRR6650825*	HiSeq 2500	218,933,136	101	22,112,246,736	
Short-eared owl	SRR6650845*	HiSeq 4000	298,420,942	151	45,061,562,242	37.55
Japanese scops-owl	SRR6650842*	HiSeq 4000	344,542,548	151	52,025,924,748	43.35
Barn owl	SRR959575	HiSeq 2000	202,081,972	100	20,208,197,200	26.31
	SRR959576	HiSeq 2000	113,588,130	100	11,358,813,000	
Bald eagle	SRR1176792	HiSeq 2000	164,760,490	100	16,476,049,000	40.91
	SRR1176807	HiSeq 2000	162,569,390	100	16,256,939,000	
	SRR1176808	HiSeq 2000	163,612,546	100	16,361,254,600	
Golden eagle	SRR1586158	HiSeq 2000	195,084,988	100	19,508,498,800	34.08
	SRR1586160	HiSeq 2000	213,829,936	100	21,382,993,600	
Eurasian sparrowhawk	SRR6650840*	HiSeq 2500	504,924,004	101	50,997,324,404	42.50
Northern goshawk	SRR6650838*	HiSeq 2500	508,628,750	101	51,371,503,750	42.81
Cinereous vulture	SRR1251975	HiSeq 2000	472,000,000	100	47,200,000,000	39.33
White-tailed eagle	SRR6650836*	HiSeq 4000	295,742,950	151	44,657,185,450	37.21
Oriental honey-buzzard	SRR6650837*	HiSeq 4000	319,662,296	151	48,269,006,696	40.22
Black kite	SRR6650833*	HiSeq 4000	325,611,358	151	49,167,315,058	40.97
Chinese sparrowhawk	SRR6650832*	HiSeq 4000	267,398,406	151	40,377,159,306	33.65
Turkey vulture	SRR954275	HiSeq 2000	182,156,980	100	18,215,698,000	31.02
	SRR954276	HiSeq 2000	190,104,648	100	19,010,464,800	
Saker falcon	SRR516278	HiSeq 2000	126,646,056	100	12,664,605,600	40.48
	SRR516279	HiSeq 2000	203,861,744	100	20,386,174,400	
	SRR516280	HiSeq 2000	155,290,250	100	15,529,025,000	
Peregrine falcon	SRR516257	HiSeq 2000	167,855,706	100	16,785,570,600	41.17
	SRR516258	HiSeq 2000	153,850,852	100	15,385,085,200	
	SRR516261	HiSeq 2000	172,349,170	100	17,234,917,000	
Eurasian hobby	SRR6650830*	HiSeq 2500	500,017,118	101	50,501,728,918	42.08
Grey-headed woodpecker	SRR6650828*	HiSeq 2000	250,918,932	100	25,091,893,200	20.91
Medium ground-finch	SRR448683	HiSeq 2000	210,033,386	100	21,003,338,600	37.34
	SRR448686	HiSeq 2000	238,058,756	100	23,805,875,600	
White-throated sparrow	SRR1796638	HiSeq 2000	170,444,906	100	17,044,490,600	29.75
	SRR1796642	HiSeq 2000	186,544,754	100	18,654,475,400	
Common canary	SRR2895902	HiSeq 1500	357,144,420	100	35,714,442,000	29.76
Collared flycatcher	ERR637368	HiSeq 2000	372,990,014	100	37,299,001,400	31.08
Little egret	SRR6650826*	HiSeq 2000	227,285,376	100	22,728,537,600	18.94
Striated heron	SRR6650834*	HiSeq 2000	227,895,338	100	22,789,533,800	18.99
Black-faced spoonbill	SRR6650847*	HiSeq 2000	245,189,378	100	24,518,937,800	20.43
Chuck-will's-widow	SRR953452	HiSeq 2000	213,356,630	100	21,335,663,000	35.90
	SRR953453	HiSeq 2000	217,467,226	100	21,746,722,600	
Brown kiwi	ERR519284	HiSeq 2500	322,457,250	100	32,245,725,000	32.88
	ERR519285	HiSeq 2500	72,064,372	100	7,206,437,200	

* denotes whole genome sequences obtained from this study

d. Transcriptome sequencing

Common name	Accession	Platform	Number of reads	Read length (bp)	Total bases (bp)
Eurasian eagle-owl	SRR6650822*	HiSeq 2000	74,807,936	100	7,480,793,600
Oriental scops-owl	SRR6650823*	HiSeq 2000	80,047,874	100	8,004,787,400
Himalayan owl	SRR6650818*	HiSeq 2000	80,236,848	100	8,023,684,800
Northern boobook	SRR6650817*	HiSeq 2000	73,698,344	100	7,369,834,400
Long-eared owl	SRR6650844*	HiSeq 2000	72,575,180	100	7,257,518,000
Eastern buzzard	SRR6650843*	HiSeq 2500	65,713,128	101	6,637,025,928
Eurasian sparrowhawk	SRR6650841*	HiSeq 2500	64,419,598	101	6,506,379,398
Northern goshawk	SRR6650839*	HiSeq 2500	105,925,434	101	10,698,468,834
Cinereous vulture	SRR1265955	HiSeq 2500	60,236,628	100	6,023,662,800
Common kestrel	SRR6650831*	HiSeq 2500	64,250,348	101	6,489,285,148
Saker falcon	SRR522907	HiSeq 2000	76,863,908	100	7,686,390,800
Peregrine falcon	SRR522906	HiSeq 2000	79,571,270	100	7,957,127,000
Eurasian hobby	SRR6650829	HiSeq 2500	60,518,768	101	6,112,395,568
Grey-headed woodpecker	SRR6650827*	HiSeq 2000	79,112,614	100	7,911,261,400
Eurasian siskin	SRR1551783	HiSeq 2000	41,742,212	65, 90	3,801,435,235
Little egret	SRR6650835*	HiSeq 2500	77,748,608	100	7,774,860,800
Striated heron	SRR6650846*	HiSeq 2000	73,160,250	100	7,316,025,000
Black-faced spoonbill	SRR6650848*	HiSeq 2000	82,694,972	100	8,269,497,200

* denotes transcriptome sequences obtained from this study

Table S12. Transposable element statistics for the four bird of prey genomes

a. Eurasian eagle-owl

Type	*Ab initio* based (bp)	Homology based (bp)	Total (bp)	Percentage of genome (%)
DNA	5,321,830	4,528,204	6,016,724	0.48%
LINE	59,572,100	52,388,102	59,874,266	4.76%
LTR	17,695,841	14,253,031	17,788,511	1.41%
Low_complexity	1,957,049	2,017,594	2,025,077	0.16%
Retroposon	1,256	-	1,256	0.00%
SINE	969,396	1,064,867	1,198,180	0.10%
Satellite	442,417	151,797	468,838	0.04%
Simple_repeat	9,072,263	10,318,708	10,336,793	0.82%
TandemRepeat	-	-	21,521,428	1.71%
Unknown	771,686	740,728	867,230	0.07%
Unspecified	2,767,143	-	2,767,143	0.22%
Total_TE	97,746,927	85,408,455	114,298,851	9.09%

b. Oriental scops-owl

Type	*Ab initio* based (bp)	Homology based (bp)	Total (bp)	Percentage of genome (%)
DNA	5,263,514	4,327,304	5,927,333	0.48%
LINE	56,913,491	49,993,098	57,258,859	4.65%
LTR	19,759,573	15,127,553	19,852,639	1.61%
Low_complexity	2,243,449	111,234	2,275,506	0.18%
Retroposon	2,276	-	2,276	0.00%
SINE	913,404	1,018,313	1,136,775	0.09%
Satellite	548,057	271,720	596,703	0.05%
Simple_repeat	9,177,656	1,474,546	9,732,434	0.79%
TandemRepeat	-	-	13,019,635	1.06%
Unknown	759,745	725,087	847,083	0.07%
Unspecified	2,469,624	-	2,469,624	0.20%
Total_TE	97,219,820	72,984,692	107,200,077	8.71%

c. Eastern buzzard

Type	*Ab initio* based (bp)	Homology based (bp)	Total (bp)	Percentage of genome (%)
DNA	5,603,025	5,007,155	6,396,464	0.51%
LINE	41,381,034	33,893,267	41,735,462	3.31%
LTR	31,511,623	27,354,821	31,623,377	2.51%
Low_complexity	2,133,269	2,190,607	2,197,521	0.17%
Retroposon	1,156	-	1,156	0.00%
SINE	1,060,451	1,185,014	1,319,029	0.10%
Satellite	431,142	198,737	458,150	0.04%
Simple_repeat	8,292,936	9,347,676	9,366,893	0.74%
TandemRepeat	-	-	18,908,531	1.50%
Unknown	813,988	783,932	917,221	0.07%
Unspecified	2,408,856	-	2,408,856	0.19%
Total_TE	92,877,476	79,864,568	107,687,623	8.55%

d. Common kestrel

Type	*Ab initio* based (bp)	Homology based (bp)	Total (bp)	Percentage of genome (%)
DNA	4,257,324	3,739,434	5,142,337	0.44%
LINE	43,063,180	37,529,708	43,510,124	3.69%
LTR	15,362,220	12,819,987	15,428,962	1.31%
Low_complexity	2,518,248	2,583,944	2,591,422	0.22%
Retroposon	881	-	881	0.00%
SINE	662,953	883,172	955,565	0.08%
Satellite	207,775	102,354	232,366	0.02%
Simple_repeat	9,804,775	10,734,936	10,751,267	0.91%
TandemRepeat	-	-	8,284,031	0.70%
Unknown	614,646	657,964	735,905	0.06%
Unspecified	1,645,735	-	1,645,735	0.14%
Total_TE	77,520,599	68,999,966	83,921,845	7.11%

Table S13. Whole genome sequencing and mapping statistics for the birds of prey and non-raptor birds

Species	Reference assembly	The number of sequence reads	The number of mapped reads	Mapping rate (%)	Estimated sequencing depth from mapped reads (×)
Eurasian eagle-owl	Eurasian eagle-owl	426,406,536	423,883,463	99.41	35.63
Oriental scops-owl	Oriental scops-owl	509,968,954	497,671,220	97.59	41.47
	Eurasian eagle-owl	509,968,954	484,464,677	95.00	40.72
Himalayan owl	Eurasian eagle-owl	475,120,796	463,937,275	97.65	38.79
Northern spotted owl	Northern spotted owl	342,525,114	329,308,129	96.14	39.93
	Eurasian eagle-owl	342,525,114	322,365,208	94.11	40.25
Barred owl	Northern spotted owl	254,293,316	253,085,927	99.53	30.69
	Eurasian eagle-owl	254,293,316	249,849,100	98.25	31.19
Northern boobook	Eurasian eagle-owl	483,070,632	456,180,964	94.43	38.13
Long-eared owl	Eurasian eagle-owl	534,446,094	517,804,343	96.89	43.32
Short-eared owl	Eurasian eagle-owl	298,420,942	292,213,676	97.92	36.72
Japanese scops-owl	Eurasian eagle-owl	344,542,548	338,549,210	98.26	42.55
Barn owl	Eurasian eagle-owl	315,670,102	250,294,698	79.29	20.83
Eastern buzzard	Eastern buzzard	383,420,678	381,454,295	99.49	31.68
Bald eagle	Bald eagle	490,942,426	479,287,574	97.63	41.34
	Eastern buzzard	490,942,426	482,644,359	98.31	39.69
Golden eagle	Golden eagle	408,914,924	403,943,772	98.78	34.23
	Eastern buzzard	408,914,924	397,378,332	97.18	32.68
Eurasian sparrowhawk	Eastern buzzard	504,924,004	485,432,318	96.14	40.32
Northern goshawk	Eastern buzzard	508,628,750	491,544,168	96.64	40.82
Cinereous vulture	Eastern buzzard	472,000,000	463,442,066	98.19	38.11
White-tailed eagle	Eastern buzzard	295,742,950	292,462,759	98.89	36.31
Oriental honey-buzzard	Eastern buzzard	319,662,296	308,671,755	96.56	38.33
Black kite	Eastern buzzard	325,611,358	319,810,066	98.22	39.71
Chinese sparrowhawk	Eastern buzzard	267,398,406	262,592,596	98.20	32.61
Turkey vulture	Eastern buzzard	372,261,628	301,706,530	81.05	24.81
Common kestrel	Common kestrel	531,996,158	518,208,664	97.41	44.62
Saker falcon	Saker falcon	485,798,050	479,708,329	98.75	41.68
	Common kestrel	485,798,050	472,692,978	97.30	40.30
Peregrine falcon	Peregrine falcon	494,055,728	490,597,776	99.30	42.53
	Common kestrel	494,055,728	484,437,741	98.05	41.30
Eurasian hobby	Common kestrel	500,017,118	485,539,341	97.10	41.80
American crow	Zebra finch	738,841,632	527,764,698	71.43	43.16
Medium ground-finch	Zebra finch	448,092,142	356,172,483	79.49	29.13
White-throated sparrow	Zebra finch	356,989,660	296,542,972	83.07	24.25
Common canary	Zebra finch	357,144,420	261,161,690	73.12	21.57
Collared flycatcher	Zebra finch	372,990,014	303,709,377	81.43	24.84

Table S14. Variant statistics for the birds of prey and non-raptor birds

Species	Reference assembly	All variant sites	Homozygous SNV sites	Heterozygous SNV sites	Indel sites
Eurasian eagle-owl	Eurasian eagle-owl	2,007,402	27,807	1,808,718	170,877
Oriental scops-owl	Oriental scops-owl	8,644,348	19,715	7,880,483	744,150
	Eurasian eagle-owl	45,212,978	34,196,869	8,871,144	2,144,965
Himalayan owl	Eurasian eagle-owl	30,233,090	27,005,757	1,908,218	1,319,115
Northern spotted owl	Northern spotted owl	380,227	55,064	255,118	70,045
	Eurasian eagle-owl	29,463,889	25,445,792	1,015,331	3,002,766
Barred owl	Northern spotted owl	11,863,926	7,598,827	3,089,938	1,175,161
	Eurasian eagle-owl	30,121,988	23,604,477	3,413,634	3,103,877
Northern boobook	Eurasian eagle-owl	52,462,194	47,733,068	2,518,712	2,210,414
Long-eared owl	Eurasian eagle-owl	35,670,717	30,317,154	3,709,009	1,644,554
Short-eared owl	Eurasian eagle-owl	37,742,200	29,588,416	6,187,653	1,966,131
Japanese scops-owl	Eurasian eagle-owl	43,590,517	37,348,510	4,329,977	1,912,030
Barn owl	Eurasian eagle-owl	77,562,229	73,075,569	2,010,995	2,475,665
Eastern buzzard	Eastern buzzard	2,283,626	14,293	2,088,450	180,883
Bald eagle	Bald eagle	1,010,177	97,023	807,219	105,935
	Eastern buzzard	20,542,155	18,490,544	630,353	1,421,258
Golden eagle	Golden eagle	1,835,290	18,725	1,682,140	134,425
	Eastern buzzard	38,382,222	34,751,436	2,177,793	1,452,993
Eurasian sparrowhawk	Eastern buzzard	42,577,883	37,628,360	3,174,952	1,774,571
Northern goshawk	Eastern buzzard	39,466,874	35,855,277	1,959,888	1,651,709
Cinereous vulture	Eastern buzzard	39,205,764	35,746,288	1,914,278	1,545,198
White-tailed eagle	Eastern buzzard	30,118,652	27,187,103	1,689,202	1,242,347
Oriental honey-buzzard	Eastern buzzard	58,016,817	51,403,339	4,390,175	2,223,303
Black kite	Eastern buzzard	32,182,132	28,728,716	2,132,391	1,321,025
Chinese sparrowhawk	Eastern buzzard	42,894,254	37,580,432	3,493,265	1,820,557
Turkey vulture	Eastern buzzard	75,410,762	70,610,796	2,356,688	2,443,278
Common kestrel	Common kestrel	5,698,946	17,843	5,152,212	528,891
Saker falcon	Saker falcon	1,740,466	104,111	1,453,643	182,712
	Common kestrel	18,704,134	16,177,271	1,579,414	947,449
Peregrine falcon	Peregrine falcon	1,598,481	84,009	1,335,088	179,384
	Common kestrel	18,644,180	16,241,792	1,467,194	935,194
Eurasian hobby	Common kestrel	20,292,235	16,229,518	2,927,043	1,135,674
American crow	Zebra finch	73,241,772	70,138,649	1,579,705	1,523,418
Medium ground-finch	Zebra finch	64,983,101	60,762,787	2,320,313	1,900,001
White-throated sparrow	Zebra finch	63,988,552	58,620,584	3,678,356	1,689,612
Common canary	Zebra finch	63,451,159	57,750,404	4,152,188	1,548,567
Collared flycatcher	Zebra finch	62,837,663	58,568,302	2,861,400	1,407,961

Table S15. Blood transcriptome *de novo* assembly and mapping statistics. RPKM is reads per kilobase per million mapped reads.

a. Transcriptome *de novo* assembly results

Species	RNA reads	Assembled transcripts	Non-bacterial transcripts	Candidate unigenes	Non-redundant unigenes	Expressed unigenes (RPKM ≥ 1.0)
Eurasian eagle-owl	74,807,936	192,557	191,773	94,871	43,765	30,948
Oriental scops-owl	80,047,874	188,436	186,833	77,140	35,146	26,197
Himalayan owl	80,236,848	215,142	213,061	105,270	43,741	32,589
Northern boobook	73,698,344	226,633	225,926	113,910	52,541	40,005
Long-eared owl	72,575,180	178,865	176,769	92,214	36,725	28,065
Eastern buzzard	65,713,128	177,945	177,425	82,313	33,088	26,847
Eurasian sparrowhawk	64,419,598	166,403	166,145	75,790	30,635	25,904
Northern goshawk	105,925,434	268,641	267,370	136,735	53,835	39,255
Cinereous vulture	60,236,628	173,662	172,758	89,347	35,912	29,182
Common kestrel	64,250,348	181,982	181,774	81,852	34,577	27,956
Saker falcon	76,863,908	124,489	123,135	40,333	22,158	18,509
Peregrine falcon	79,571,270	117,666	106,738	36,892	21,002	17,750
Eurasian hobby	60,518,768	394,468	394,145	289,855	200,718	162,083
Grey-headed woodpecker	79,112,614	164,135	163,403	89,073	40,888	35,233
Eurasian siskin	41,742,212	72,078	72,030	17,846	12,775	12,619
Little egret	77,748,608	197,944	197,464	91,981	41,795	31,053
Striated heron	73,160,250	167,558	166,990	81,217	37,962	27,845
Black-faced spoonbill	82,694,972	179,098	178,488	94,107	40,848	29,368

b. Transcriptome sequences mapping results to the genome assembly

Species	RNA reads	Mapped reads	Mapping rate (%)	Concordant pair alignment rate (%)
Eurasian eagle-owl	74,807,936	58,030,942	77.6	68.7
Oriental scops-owl	80,047,874	67,101,568	83.8	74.9
Eastern buzzard	65,713,128	58,221,624	88.6	81.5
Common kestrel	64,250,348	55,612,832	86.6	80.2
Saker falcon	76,863,908	53,324,296	69.4	58.1
Peregrine falcon	79,571,270	53,987,662	67.8	59.5
Little egret	77,748,608	64,132,289	82.5	74.0

Table S16. Meta information for the 25 genome-assembled avian species used in this study

Species	Maximum longevity (yrs)	Specimen origin	Avg. body mass (g)	Nocturnality	Major diet	Ref. source
Eurasian eagle-owl	27.8	Wild	2708	Nocturnal	Carnivorous	S12, S13
Northern spotted owl	25	Wild	588.7	Nocturnal	Carnivorous	S13, S14
Oriental scops-owl	N/A (6.8 yrs for Eurasian scops-owl)	N/A (wild)	85	Nocturnal	Carnivorous	S13, S14
Barn Owl	34	Wild	580	Nocturnal	Carnivorous	S13, S14
Downy woodpecker	11.9	Wild	27.0	-	Omnivorous	S13, S14
Golden eagle	48	Captivity	4421	-	Carnivorous	S13, S14
Eastern buzzard	N/A (28.8 yrs for common buzzard)	N/A (wild)	731	-	Carnivorous	S13, S14
Bald eagle	48	Captivity	4650	-	Carnivorous	S13, S14
Saker falcon	15.9	Wild	997.5	-	Carnivorous	S13, S14
Peregrine falcon	25	Unknown	920	-	Carnivorous	S13, S14
Common kestrel	23.8	Wild	214	-	Carnivorous	S13, S14
Zebra Finch	12	Unknown	10	-	Herbivorous	S13, S14
American crow	20	Unknown	412.5	-	Omnivorous	S13, S14
Budgerigar	21	Captivity	27.5	-	Herbivorous	S13, S14
Little egret	22.3	Wild	495	-	Carnivorous	S13, S14, S15
Crested Ibis	25.8	Captivity	1900	-	Carnivorous	S13, S14
Hoatzin	14	Captivity	797.5	-	Herbivorous	S13, S16
Killdeer	10.9	Wild	96.5	-	Omnivorous	S13, S14, S17
Chuck-will's-widow	14.8	Wild	116.3	Nocturnal	Carnivorous	S13, S14
Anna's hummingbird	8.5	Wild	4.3	-	Omnivorous	S13, S14, S17
Common cuckoo	12.9	Wild	115	-	Carnivorous	S13, S14
Rock dove	6.0	Wild	270	-	Herbivorous	S13, S17
Chicken	30	Captivity	914	-	Omnivorous	S13, S14
Brown kiwi	35	Captivity	2527.5	Nocturnal	Omnivorous	S13, S14
Common ostrich	50	Captivity	111000.0	-	Herbivorous	S13, S14

Table S17. Commonly enriched Gene Ontology (GO) categories of expanded gene families in the ancestral branches of Strigiformes, Accipitriformes, and Falconiformes. GO categories also enriched in expanded gene families of brown kiwi were excluded. *P*-value was calculated by Fisher's exact test with a 5% FDR criterion.

GO term	Description	*P*-value in the ancestral branch of Strigiformes	*P*-value in the ancestral branch of Accipitriformes	*P*-value in the ancestral branch of Falconiformes
GO:0007010	cytoskeleton organization	1.13E-07	6.54E-03	3.34E-03
GO:0022603	regulation of anatomical structure morphogenesis	6.27E-07	1.58E-04	2.36E-02
GO:0050877	nervous system process	8.22E-07	1.14E-02	1.80E-02
GO:0044449	contractile fiber part	1.13E-06	8.97E-06	1.65E-02
GO:0007605	sensory perception of sound	4.99E-06	2.05E-03	1.49E-02
GO:0003779	actin binding	5.17E-06	2.88E-03	1.28E-02
GO:0044430	cytoskeletal part	5.42E-06	1.94E-03	2.84E-02
GO:0050954	sensory perception of mechanical stimulus	7.09E-06	2.34E-03	1.60E-02
GO:0031982	vesicle	2.05E-05	3.65E-06	1.02E-03
GO:0051094	positive regulation of developmental process	3.23E-05	4.76E-02	1.37E-02
GO:0050793	regulation of developmental process	5.82E-05	6.41E-03	4.93E-02
GO:0098590	plasma membrane region	7.18E-05	6.82E-04	4.02E-02
GO:0120035	regulation of plasma membrane bounded cell projection organization	1.23E-04	4.02E-04	4.03E-03
GO:0000226	microtubule cytoskeleton organization	2.11E-04	2.86E-02	9.07E-03
GO:0044087	regulation of cellular component biogenesis	2.34E-04	8.43E-03	1.91E-02
GO:0044421	extracellular region part	2.38E-04	8.18E-06	1.46E-03
GO:0005829	cytosol	2.68E-04	3.45E-02	8.48E-03
GO:0071702	organic substance transport	3.31E-04	1.48E-03	3.64E-02
GO:0005604	basement membrane	3.33E-04	1.26E-04	3.96E-03
GO:0070062	extracellular exosome	4.61E-04	8.68E-06	2.82E-03
GO:0043230	extracellular organelle	5.20E-04	9.79E-06	3.00E-03
GO:0045597	positive regulation of cell differentiation	6.21E-04	6.61E-03	1.75E-02
GO:0050896	response to stimulus	6.30E-04	2.02E-02	3.52E-02
GO:0030004	cellular monovalent inorganic cation homeostasis	8.38E-04	2.21E-02	3.53E-03
GO:0044420	extracellular matrix component	1.10E-03	3.42E-04	6.76E-03
GO:0031012	extracellular matrix	1.48E-03	4.13E-04	1.36E-02
GO:0014069	postsynaptic density	1.55E-03	6.28E-04	1.00E-02
GO:0099572	postsynaptic specialization	1.55E-03	6.28E-04	1.00E-02
GO:0031346	positive regulation of cell projection organization	2.20E-03	1.52E-02	4.91E-03
GO:0034612	response to tumor necrosis factor	2.66E-03	4.93E-03	7.17E-03
GO:0055067	monovalent inorganic cation homeostasis	2.66E-03	3.73E-02	6.19E-03
GO:0045595	regulation of cell differentiation	2.83E-03	1.35E-02	3.74E-02
GO:0007612	learning	3.19E-03	1.89E-02	1.81E-02
GO:0031589	cell-substrate adhesion	3.75E-03	1.63E-02	8.94E-04
GO:0006508	proteolysis	4.21E-03	4.16E-03	9.45E-03
GO:0007160	cell-matrix adhesion	4.72E-03	5.34E-03	2.80E-04
GO:0045664	regulation of neuron differentiation	7.05E-03	2.47E-03	2.93E-02
GO:0098542	defense response to other organism	1.01E-02	4.51E-02	3.24E-02
GO:0071356	cellular response to tumor necrosis factor	1.16E-02	2.98E-02	4.45E-03
GO:0051707	response to other organism	1.32E-02	2.90E-02	7.87E-03
GO:0009605	response to external stimulus	1.43E-02	3.41E-02	3.90E-03
GO:1902115	regulation of organelle assembly	1.85E-02	1.89E-02	2.06E-02
GO:0010033	response to organic substance	1.98E-02	4.97E-03	3.60E-02
GO:0050773	regulation of dendrite development	3.59E-02	6.45E-03	8.21E-03
GO:0006810	transport	3.86E-02	4.88E-02	3.88E-02
GO:0071347	cellular response to interleukin-1	3.96E-02	1.32E-02	2.00E-03

Table S18. List of genes showing accelerated d_N/d_S in the three raptor orders

Genes	ω Three raptor branches				ω outgroups
	Strigiformes	Accipitriformes	Falconiformes	Merged	
LOC422316	0.7250	0.2188	0.3676	0.4146	0.1253
RNH1	0.5599	0.3043	0.5248	0.4822	0.1340
RIF1	0.4231	0.5985	0.6027	0.5556	0.3007
VLDLR	0.4052	0.5618	0.4375	0.4731	0.1466
IL4I1	1.3922	0.2168	0.3847	0.4886	0.1801
SKP2	1.3782	0.6191	0.7180	0.9569	0.2997
SMC4	0.4212	0.4064	0.4548	0.4350	0.2166
MUM1	0.9040	0.5793	0.6939	0.7164	0.3299
ORC1	0.5132	0.4786	1.0003	0.7373	0.3508
DNAJC25	1.1660	0.1189	0.4075	0.3706	0.0647
COL3A1	0.5096	0.2897	0.2788	0.3436	0.1746
GMPS	0.1401	0.0521	0.1685	0.1259	0.0166
ITFG1	0.4411	0.4819	0.5602	0.5029	0.1608
LMNA	1.0153	0.9055	0.8907	0.9214	0.4318
DNAJC21	0.5231	0.8603	0.4373	0.6237	0.2616
HELB	0.7658	1.1112	0.4070	0.7014	0.3765
C5	0.4088	0.4190	0.4032	0.4098	0.2447
LOC107048990	0.7351	0.2973	0.3189	0.4411	0.2321
MRPL37	0.7049	0.9437	0.9591	0.8887	0.2786
NCAPG	0.6532	0.5192	0.6578	0.6243	0.3423
TSPO2	4.1893	0.4787	1.2710	1.2814	0.1887
RAD50	0.1508	0.2132	0.2709	0.2286	0.1179
SLC22A7	0.4227	0.4236	0.4662	0.4376	0.2099
SMC6	0.6001	0.3487	0.5704	0.5198	0.3019
ETV4	0.3241	0.7722	0.5582	0.5281	0.2409
LEMD2	0.5947	0.4558	0.4652	0.4546	0.2083
KCNQ1	0.2666	0.0690	0.2292	0.1870	0.0644
CENPC	0.9169	1.3188	1.3028	1.1991	0.7251
SPCS1	0.1741	3.2942	0.6508	1.3882	0.1456
ELAVL2	0.2034	0.0607	0.3010	0.1946	0.0587
KNTC1	0.3165	0.2435	0.4318	0.3490	0.2371
KIAA1551	1.1546	1.3174	0.7757	0.9800	0.6994
CENPL	0.7673	0.5784	0.4658	0.5660	0.2346
DIS3L	0.1537	0.4051	0.1708	0.2379	0.1297
NSMCE4A	1.1903	0.4201	0.8415	0.6702	0.2963
GLIS3	0.9297	0.6221	0.8195	0.7836	0.4803
RHCE	1.3586	1.6996	1.0154	1.2200	0.5970
SNAP47	0.3797	0.1633	0.3045	0.2815	0.1036
TCFL5	0.8234	0.4243	0.5573	0.6004	0.3415
GFM2	0.3309	0.2564	0.4258	0.3401	0.1830
AP1AR	0.4730	0.2743	0.3456	0.3500	0.0881
ORC2	0.8864	0.4046	0.6894	0.6752	0.3308
NANOG	0.7016	0.3990	0.9100	0.6286	0.2764
DNAJC8	0.4367	0.8052	0.0335	0.1867	0.0286
PRPH2	0.4387	0.1431	0.2196	0.2403	0.0870
LOC770429	0.5131	0.2316	0.3097	0.3657	0.1871
FOCAD	0.4374	0.3309	0.4922	0.4310	0.3004
LIG4	0.1824	0.2141	0.3160	0.2448	0.1378
POLRMT	0.2583	0.2572	0.3023	0.2782	0.1846
SS18L1	0.8364	0.1186	0.0581	0.1452	0.0255
KBTBD2	0.0436	0.0154	0.0575	0.0432	0.0099
KPNA7	0.2822	0.3663	0.6127	0.4376	0.2511
MMP1	0.4442	0.9045	0.4847	0.5787	0.2872
RAD21L1	0.6515	0.4188	0.7522	0.6250	0.3461
MIS12	0.4099	0.2780	0.3949	0.3795	0.1602
TBPL2	0.5624	0.3218	0.6313	0.4961	0.2344
LRWD1	0.2260	0.2716	0.4269	0.3063	0.1751
POLR2D	0.0427	0.3664	0.2344	0.1768	0.0251
KIAA1033	0.1552	0.1328	0.1589	0.1366	0.0712
UBE2G2	0.1511	0.0872	0.2136	0.1675	0.0233
TMEM116	0.3217	0.3364	0.3495	0.3190	0.1477

STX8	0.7933	0.1897	0.1626	0.3038	0.1201
CNTD1	1.2119	0.2612	0.5072	0.4879	0.2434
NUP93	0.2211	0.1128	0.1113	0.1364	0.0570
OVOL2	0.1540	0.0696	0.3300	0.1984	0.0502
DBF4	0.4026	0.6240	0.6836	0.5856	0.3440
TECPR1	0.2763	0.2596	0.1825	0.2300	0.1396
MMP10	0.4672	0.3015	0.4835	0.4404	0.2370
LOC100859557	0.5256	0.6479	0.4993	0.5387	0.3572
MEIOB	0.4184	0.2747	0.3330	0.3513	0.1727
ALKBH4	0.4927	0.4464	0.5723	0.5166	0.1860
ING3	0.1429	0.0374	0.2016	0.1333	0.0313
TOPORS	0.4527	0.4135	0.4088	0.4233	0.2882
TGM6L	0.3119	0.2491	0.3588	0.3217	0.1732
ZDHHC12	0.8205	0.6576	0.5786	0.6555	0.2401
HP1BP3	0.2248	0.1323	0.2448	0.2165	0.0939
SLC24A1	0.5810	0.4383	0.3935	0.4639	0.2598
SRP68	0.1117	0.1310	0.0533	0.0921	0.0299
TMEM175	0.4089	0.2286	0.3711	0.3423	0.1590
SPRED2	0.1132	0.1351	0.0933	0.1109	0.0270
TMEM9B	0.0789	1.9266	0.0842	0.2112	0.0232
WDR76	0.2154	0.3973	0.3036	0.3042	0.1728
RHAG	0.5409	0.9078	0.9742	0.8175	0.4571
CPLX4	0.7597	0.0882	0.1211	0.2543	0.0574
A1CF	0.1122	0.1163	0.0611	0.0928	0.0419
MYEF2	0.3063	0.3647	0.2974	0.3117	0.1458
TMEM136-2	0.2489	0.1882	0.2055	0.1874	0.0834
NDUFS3	0.3365	0.2764	0.2446	0.2422	0.0787
ANO5	0.2622	0.0901	0.0808	0.1343	0.0784
FUT11	0.2902	0.3001	0.1422	0.2026	0.1027
L2HGDH	0.2944	0.1769	0.2452	0.2524	0.1130
TRIM25	0.8545	0.9449	0.6694	0.7773	0.4986
GGT5	0.4894	0.6780	0.4471	0.5422	0.3287
FLI1	0.0711	0.2944	0.0851	0.1456	0.0404
MAK16	0.1454	0.3380	0.0665	0.1538	0.0638
UVSSA	0.4039	0.3080	0.4887	0.4105	0.2612
AAGAB	0.2813	0.2481	0.6144	0.3637	0.1667
SNAPC3	0.6118	0.1719	0.4173	0.4124	0.1666
SOD1	0.7430	1.1950	0.7399	0.8194	0.2546
MUC4	0.5334	0.5018	0.3805	0.4498	0.3563
TRMT10C	1.7110	0.3671	0.4010	0.5465	0.3053
SURF6	0.4193	0.3794	0.3994	0.4062	0.2039
CDC37L1	0.7698	0.4973	0.3091	0.4928	0.2516
RMDN3	0.3245	0.2037	0.2693	0.2695	0.1439
FUBP3	0.5092	0.2864	0.1051	0.1954	0.0688
AREG	0.4658	0.2970	0.6075	0.4470	0.1661
MED7	0.0504	0.1272	0.2549	0.1925	0.0479
TPX2	0.3119	0.5454	0.4831	0.4414	0.2940
N4BP2L2	1.0204	0.6232	1.1135	0.8609	0.5532
MRPS11	2.3194	0.5127	0.4985	0.6119	0.1897
ORC3	0.7933	0.4183	0.5177	0.5589	0.3409
APITD1	1.1615	0.3422	0.3609	0.5579	0.2415
CDC20	0.2017	0.2089	0.2715	0.2410	0.1270
CD14	0.4434	0.4728	0.4999	0.4836	0.2875
MMD	0.0797	0.3431	0.0983	0.1359	0.0378
RPAIN	0.5969	0.4616	0.3522	0.4030	0.1821
CHODL	0.2043	0.2240	0.4596	0.3084	0.1409
CEP63	0.5881	0.6250	0.5379	0.5697	0.3658
MTHFS	0.7633	1.1967	0.3747	0.8158	0.1910
DCAF5	0.1936	0.3209	0.1686	0.2083	0.1221
EIF2AK4	0.1558	0.2401	0.1963	0.1998	0.1384
MARS2	0.1934	0.2755	0.2914	0.2675	0.1497
USP30	0.2130	0.2193	0.1992	0.2153	0.1030
RD3	0.6943	0.2254	0.2965	0.3380	0.1105
MGP	0.4539	0.6842	1.3075	0.8335	0.3609
SLMO1	0.1772	0.5650	0.1384	0.2609	0.0923

C3H2orf71	0.6408	0.4491	0.4959	0.5177	0.3859
UTP18	0.3422	0.3808	0.2709	0.3220	0.1941
NUDT19	0.8512	0.4439	0.3953	0.5564	0.2914
OCSTAMP	0.5514	0.4786	0.6797	0.6036	0.3782
PLSCR5	0.3566	0.2182	0.4803	0.3904	0.1749
OPN4-1	0.3445	0.2404	0.4861	0.3658	0.2214
SLC30A5	0.1695	0.1483	0.0900	0.1201	0.0584
RALGAPB	0.0561	0.0569	0.0802	0.0673	0.0372
RLF	0.1986	0.1728	0.2291	0.2093	0.1540
ABCB7	0.1412	0.2699	0.1560	0.1740	0.0917
PPARA	0.0758	0.0634	0.1495	0.1101	0.0358
ACRC	0.4677	0.4080	0.7583	0.5544	0.3506
LOC428778	0.5008	0.4180	0.4442	0.4568	0.2842
LRRC57	0.1208	0.3721	0.1769	0.2066	0.0670
N4BP1	0.5323	0.3726	0.6144	0.5338	0.3709
MRPL45	0.2253	0.2366	0.4585	0.3219	0.1530
METTL6	0.3089	0.3451	0.5078	0.3894	0.1765
ORC6	0.9626	0.3750	0.6522	0.5851	0.3124
PARL	0.2813	0.4540	0.1711	0.2587	0.1026
RP5-1028K7.3	0.7597	1.6145	0.2441	0.4241	0.1928
SMG8	0.1183	0.0688	0.0875	0.0894	0.0511
CCNA2	0.2925	0.4920	0.3058	0.3211	0.1584

Table S19. Gene Ontology (GO) enrichment of genes that have a high level of GC3 biases in the bird of prey genomes. *P*-value was calculated by Fisher's exact test. Only GO categories with *P* < 5.00E-03 are shown.

GO term	Description	*P*-value	FDR
GO:0006928	movement of cell or subcellular component	2.43E-04	9.12E-02
GO:0070309	lens fiber cell morphogenesis	4.04E-04	9.12E-02
GO:0033077	T cell differentiation in thymus	6.14E-04	9.12E-02
GO:0033151	V(D)J recombination	6.14E-04	9.12E-02
GO:0040011	locomotion	9.58E-04	9.12E-02
GO:0002568	somatic diversification of T cell receptor genes	1.20E-03	9.12E-02
GO:0033153	T cell receptor V(D)J recombination	1.20E-03	9.12E-02
GO:0002681	somatic recombination of T cell receptor gene segments	1.20E-03	9.12E-02
GO:0048538	thymus development	2.47E-03	9.12E-02
GO:0051965	positive regulation of synapse assembly	2.47E-03	9.12E-02
GO:0021953	central nervous system neuron differentiation	3.05E-03	9.12E-02
GO:0030217	T cell differentiation	3.23E-03	9.12E-02
GO:0002200	somatic diversification of immune receptors	3.69E-03	9.12E-02
GO:0016444	somatic cell DNA recombination	3.69E-03	9.12E-02
GO:0002562	somatic diversification of immune receptors via germline recombination within a single locus	3.69E-03	9.12E-02
GO:0002089	lens morphogenesis in camera-type eye	3.88E-03	9.12E-02
GO:0048870	cell motility	5.00E-03	9.12E-02
GO:0051963	regulation of synapse assembly	5.23E-03	9.12E-02
GO:0060561	apoptotic process involved in morphogenesis	5.75E-03	9.12E-02
GO:1902742	apoptotic process involved in development	5.75E-03	9.12E-02
GO:0005539	glycosaminoglycan binding	5.95E-03	9.12E-02
GO:0097485	neuron projection guidance	6.18E-03	9.12E-02
GO:0007411	axon guidance	6.18E-03	9.12E-02
GO:0008283	cell proliferation	6.38E-03	9.12E-02
GO:0051960	regulation of nervous system development	6.58E-03	9.12E-02
GO:0072376	protein activation cascade	7.94E-03	9.12E-02
GO:0000904	cell morphogenesis involved in differentiation	8.16E-03	9.12E-02
GO:0016477	cell migration	8.76E-03	9.12E-02
GO:0008219	cell death	9.18E-03	9.12E-02
GO:0050807	regulation of synapse organization	9.31E-03	9.12E-02
GO:0045165	cell fate commitment	9.31E-03	9.12E-02
GO:0030154	cell differentiation	9.61E-03	9.12E-02

Table S20. Statistics regarding highly conserved regions in Strigiformes, Accipitriformes, Falconiformes, and Passeriformes

a. Identification of highly conserved regions (HCRs) in the four avian orders

Orders	Reference genome size	The number of windows (100Kb window, >80% of sufficiently covered)		Highly conserved windows (100Kb window, Adjusted *P*-value < 0.0001)		
		Window count	Non-overlapped length (bp)	Window count	Non-overlapped length (bp)	Percentage
Strigiformes	1,258,075,470	117,593	1,211,056,698	31,477	509,623,240	42.08%
Accipitriformes	1,259,752,395	117,508	1,215,179,433	35,908	579,312,028	47.67%
Falconiformes	1,179,916,286	113,047	1,148,371,940	28,695	454,032,712	39.54%
Passeriformes	1,232,135,591	103,837	1,046,118,050	27,338	420,336,735	40.18%

b. Statistics of genes present in the HCRs

Species	Total number of genes in HCR regions (with 50% coverage of gene)	# of specific genes	# of exclusively shared genes among the three bird of prey orders	# of shared genes among the four orders
Strigiformes	3,423	733	765	420
Accipitriformes	3,897	1,122		
Falconiformes	2,933	736		
Passeriformes	4,694	1,478	-	

Table S21. Commonly enriched Gene Ontology (GO) categories of genes in the highly conserved genomic regions (HCRs) of Strigiformes, Accipitriformes, and Falconiformes. GO categories also enriched in the HCRs of Passeriformes were excluded. *P*-value was calculated by Fisher's exact test with a 5% FDR criterion.

GO term	Description	*P*-value in Strigiformes	*P*-value in Accipitriformes	*P*-value in Falconiformes
GO:0048869	cellular developmental process	8.72E-13	1.78E-10	1.75E-24
GO:0051239	regulation of multicellular organismal process	2.87E-11	1.55E-04	2.10E-15
GO:0050794	regulation of cellular process	5.87E-11	1.79E-08	1.22E-09
GO:0050789	regulation of biological process	3.83E-10	2.53E-08	4.83E-10
GO:0048646	anatomical structure formation involved in morphogenesis	7.78E-10	7.24E-06	9.14E-14
GO:0008283	cell proliferation	9.24E-10	1.40E-05	3.21E-11
GO:0065007	biological regulation	2.98E-09	2.02E-06	1.56E-09
GO:0001763	morphogenesis of a branching structure	9.10E-09	2.34E-06	6.54E-12
GO:0051241	negative regulation of multicellular organismal process	1.22E-08	1.68E-04	3.67E-10
GO:0009888	tissue development	1.33E-08	7.38E-07	1.04E-14
GO:0010941	regulation of cell death	1.80E-08	1.29E-03	2.82E-05
GO:0061138	morphogenesis of a branching epithelium	4.63E-08	8.33E-06	1.04E-11
GO:0030278	regulation of ossification	6.19E-08	1.45E-05	9.42E-08
GO:0048754	branching morphogenesis of an epithelial tube	9.44E-08	1.34E-05	8.74E-13
GO:0048514	blood vessel morphogenesis	2.48E-07	1.13E-04	3.05E-08
GO:0090287	regulation of cellular response to growth factor stimulus	8.73E-07	5.94E-04	2.74E-07
GO:0032501	multicellular organismal process	9.60E-07	2.06E-06	2.20E-19
GO:0035295	tube development	1.44E-06	5.42E-05	2.37E-11
GO:0060429	epithelium development	1.53E-06	2.58E-05	8.96E-13
GO:0060560	developmental growth involved in morphogenesis	1.61E-06	5.95E-04	4.43E-06
GO:0048589	developmental growth	1.77E-06	2.33E-05	4.98E-09
GO:0048468	cell development	1.91E-06	8.23E-07	1.82E-13
GO:0045778	positive regulation of ossification	2.30E-06	1.45E-03	1.20E-04
GO:0033077	T cell differentiation in thymus	3.28E-06	1.24E-03	8.37E-06
GO:0045667	regulation of osteoblast differentiation	3.73E-06	6.06E-04	1.80E-04
GO:0035115	embryonic forelimb morphogenesis	4.41E-06	2.01E-05	5.27E-09
GO:0007423	sensory organ development	4.59E-06	2.18E-06	4.85E-11
GO:0035136	forelimb morphogenesis	5.16E-06	1.21E-04	7.83E-09
GO:0030217	T cell differentiation	5.56E-06	1.44E-03	3.53E-05
GO:0040007	growth	6.20E-06	3.40E-05	2.89E-08
GO:0035148	tube formation	6.38E-06	1.78E-05	4.57E-07
GO:0022603	regulation of anatomical structure morphogenesis	1.13E-05	7.73E-04	9.21E-11
GO:0001822	kidney development	1.28E-05	4.28E-06	1.27E-10
GO:0060070	canonical Wnt signaling pathway	2.71E-05	7.43E-04	3.47E-03
GO:0003006	developmental process involved in reproduction	2.88E-05	5.77E-05	8.53E-05
GO:0072164	mesonephric tubule development	4.37E-05	4.38E-04	4.00E-07
GO:0001654	eye development	5.04E-05	1.12E-06	4.70E-08
GO:0010648	negative regulation of cell communication	7.10E-05	1.64E-04	2.88E-06
GO:0072163	mesonephric epithelium development	7.39E-05	6.93E-04	1.27E-07
GO:0051271	negative regulation of cellular component movement	7.43E-05	1.41E-03	3.29E-05
GO:0001656	metanephros development	8.07E-05	1.62E-05	2.59E-06
GO:0023057	negative regulation of signaling	8.57E-05	1.41E-04	2.48E-06
GO:0072073	kidney epithelium development	8.87E-05	9.14E-04	9.66E-10
GO:0030111	regulation of Wnt signaling pathway	1.25E-04	5.18E-06	1.93E-03
GO:0048812	neuron projection morphogenesis	1.27E-04	3.34E-05	8.00E-04
GO:0042733	embryonic digit morphogenesis	1.35E-04	5.05E-05	3.91E-10
GO:0032989	cellular component morphogenesis	1.41E-04	1.61E-06	7.41E-05
GO:0048858	cell projection morphogenesis	1.42E-04	1.91E-05	7.95E-04
GO:0007178	transmembrane receptor protein serine/threonine kinase signaling pathway	1.49E-04	1.02E-03	7.00E-05
GO:0001823	mesonephros development	1.56E-04	4.17E-04	6.85E-04
GO:0022414	reproductive process	1.61E-04	3.28E-04	2.26E-04
GO:0009880	embryonic pattern specification	1.66E-04	4.97E-05	3.22E-05
GO:0048568	embryonic organ development	1.77E-04	5.68E-04	5.48E-04
GO:0120039	plasma membrane bounded cell projection morphogenesis	1.96E-04	5.65E-05	5.61E-04

GO:0048588	developmental cell growth	1.98E-04	4.92E-04	2.49E-03
GO:0007409	axonogenesis	2.34E-04	4.54E-04	2.24E-05
GO:0006928	movement of cell or subcellular component	2.47E-04	7.94E-07	1.34E-11
GO:0002062	chondrocyte differentiation	2.49E-04	1.12E-03	3.22E-05
GO:0009968	negative regulation of signal transduction	3.29E-04	1.13E-04	2.15E-05
GO:2000027	regulation of organ morphogenesis	3.37E-04	1.49E-03	7.59E-14
GO:0048585	negative regulation of response to stimulus	3.59E-04	6.32E-05	3.54E-05
GO:0003279	cardiac septum development	4.12E-04	4.96E-04	9.07E-05
GO:0010718	positive regulation of epithelial to mesenchymal transition	4.12E-04	3.65E-04	6.13E-06
GO:0010463	mesenchymal cell proliferation	4.62E-04	3.93E-04	3.17E-06
GO:0048762	mesenchymal cell differentiation	5.32E-04	1.45E-03	3.58E-06
GO:0043010	camera-type eye development	5.66E-04	1.80E-04	1.57E-05
GO:0060272	embryonic skeletal joint morphogenesis	5.75E-04	1.07E-04	3.67E-07
GO:0060828	regulation of canonical Wnt signaling pathway	6.59E-04	3.20E-05	4.08E-03
GO:0048736	appendage development	6.78E-04	6.47E-05	7.24E-04
GO:0060173	limb development	6.78E-04	6.47E-05	7.24E-04
GO:1901214	regulation of neuron death	1.20E-03	7.53E-04	1.25E-04
GO:0030279	negative regulation of ossification	1.38E-03	2.72E-04	7.56E-05
GO:0046483	heterocycle metabolic process	1.42E-03	1.37E-03	1.45E-04
GO:0010721	negative regulation of cell development	1.52E-03	3.83E-04	2.99E-06
GO:0060441	epithelial tube branching involved in lung morphogenesis	1.59E-03	7.97E-04	5.91E-04
GO:0050679	positive regulation of epithelial cell proliferation	2.16E-03	3.38E-04	2.24E-05
GO:0030178	negative regulation of Wnt signaling pathway	2.32E-03	4.18E-04	9.01E-05
GO:0072111	cell proliferation involved in kidney development	2.50E-03	2.45E-04	5.15E-05
GO:0051961	negative regulation of nervous system development	3.19E-03	1.12E-03	7.88E-04

Table S22. Commonly enriched KEGG pathways of genes in the highly conserved genomic regions (HCRs) of Strigiformes, Accipitriformes, and Falconiformes. KEGG pathways also enriched in the HCRs of Passeriformes were excluded. *P*-value was calculated by Fisher's exact test with a 5% FDR criterion.

KEGG	Description	*P*-value in Strigiformes	*P*-value in Accipitriformes	*P*-value in Falconiformes
ko04360	Axon guidance	1.88E-06	2.20E-05	1.86E-05
ko04550	Signaling pathways regulating pluripotency of stem cells	5.11E-06	2.18E-05	3.47E-05
ko05224	Breast cancer	9.93E-06	3.16E-04	1.93E-05
ko04310	Wnt signaling pathway	1.92E-05	2.18E-05	1.06E-04
ko05202	Transcriptional misregulation in cancer	2.39E-05	3.15E-04	2.35E-07
ko05225	Hepatocellular carcinoma	3.45E-05	6.22E-06	3.21E-06
ko05226	Gastric cancer	3.60E-05	3.33E-05	1.44E-05
ko05165	Human papillomavirus infection	4.28E-05	3.30E-04	8.17E-04
ko05217	Basal cell carcinoma	6.95E-05	8.15E-04	3.11E-06
ko05205	Proteoglycans in cancer	1.25E-03	1.67E-03	6.08E-05
ko05221	Acute myeloid leukemia	2.79E-03	2.29E-04	1.19E-03
ko04320	Dorso-ventral axis formation	3.06E-03	2.62E-04	1.18E-03

Table S23. Strigiformes specific GO enrichment of genes in the highly conserved genomic regions (HCRs). *P*-value was calculated by Fisher's exact test.

GO term	Description	*P*-value	FDR
GO:0032270	positive regulation of cellular protein metabolic process	8.18E-07	4.47E-05
GO:0051247	positive regulation of protein metabolic process	1.96E-06	9.49E-05
GO:0032268	regulation of cellular protein metabolic process	1.07E-05	4.36E-04
GO:0051246	regulation of protein metabolic process	1.34E-05	5.32E-04
GO:0031401	positive regulation of protein modification process	2.12E-05	8.12E-04
GO:0007498	mesoderm development	4.28E-05	1.55E-03
GO:0034483	heparan sulfate sulfotransferase activity	4.79E-05	1.72E-03
GO:0046651	lymphocyte proliferation	4.93E-05	1.74E-03
GO:0032943	mononuclear cell proliferation	4.93E-05	1.74E-03
GO:0010942	positive regulation of cell death	6.09E-05	2.08E-03
GO:0043068	positive regulation of programmed cell death	1.24E-04	3.87E-03
GO:0030501	positive regulation of bone mineralization	1.45E-04	4.34E-03
GO:0043065	positive regulation of apoptotic process	1.60E-04	4.68E-03
GO:0070169	positive regulation of biomineral tissue development	2.49E-04	6.82E-03
GO:0070372	regulation of ERK1 and ERK2 cascade	2.93E-04	7.92E-03
GO:0043408	regulation of MAPK cascade	3.01E-04	8.10E-03
GO:0001047	core promoter binding	3.36E-04	8.83E-03
GO:0048645	animal organ formation	3.50E-04	9.04E-03
GO:0010894	negative regulation of steroid biosynthetic process	3.50E-04	9.04E-03
GO:0045939	negative regulation of steroid metabolic process	3.50E-04	9.04E-03
GO:1905208	negative regulation of cardiocyte differentiation	5.09E-04	1.23E-02
GO:0043589	skin morphogenesis	5.09E-04	1.23E-02
GO:0010596	negative regulation of endothelial cell migration	5.63E-04	1.35E-02
GO:0045668	negative regulation of osteoblast differentiation	5.63E-04	1.35E-02
GO:0031399	regulation of protein modification process	5.77E-04	1.37E-02
GO:0045669	positive regulation of osteoblast differentiation	6.51E-04	1.50E-02
GO:1990138	neuron projection extension	6.78E-04	1.54E-02
GO:0010633	negative regulation of epithelial cell migration	6.78E-04	1.54E-02
GO:0008201	heparin binding	6.94E-04	1.56E-02
GO:1901343	negative regulation of vasculature development	7.80E-04	1.73E-02
GO:0060324	face development	7.88E-04	1.73E-02
GO:0045687	positive regulation of glial cell differentiation	7.88E-04	1.73E-02
GO:0060740	prostate gland epithelium morphogenesis	7.88E-04	1.73E-02
GO:0006090	pyruvate metabolic process	8.47E-04	1.84E-02
GO:0030010	establishment of cell polarity	8.83E-04	1.89E-02
GO:0000983	transcription factor activity, RNA polymerase II core promoter sequence-specific DNA binding	8.97E-04	1.91E-02
GO:0001889	liver development	9.70E-04	2.04E-02
GO:0051101	regulation of DNA binding	9.81E-04	2.05E-02
GO:0010975	regulation of neuron projection development	1.05E-03	2.17E-02
GO:0071542	dopaminergic neuron differentiation	1.07E-03	2.20E-02
GO:0044451	nucleoplasm part	1.17E-03	2.37E-02
GO:0016049	cell growth	1.17E-03	2.37E-02
GO:0019216	regulation of lipid metabolic process	1.21E-03	2.44E-02
GO:2001234	negative regulation of apoptotic signaling pathway	1.41E-03	2.78E-02
GO:0050919	negative chemotaxis	1.42E-03	2.78E-02
GO:0035282	segmentation	1.50E-03	2.87E-02
GO:0045862	positive regulation of proteolysis	1.50E-03	2.87E-02
GO:0002718	regulation of cytokine production involved in immune response	1.50E-03	2.87E-02
GO:0007369	gastrulation	1.54E-03	2.92E-02
GO:0048640	negative regulation of developmental growth	1.55E-03	2.92E-02
GO:0001655	urogenital system development	1.55E-03	2.92E-02
GO:0000790	nuclear chromatin	1.60E-03	2.98E-02
GO:0003712	transcription cofactor activity	1.67E-03	3.09E-02
GO:0004714	transmembrane receptor protein tyrosine kinase activity	1.69E-03	3.11E-02
GO:2000116	regulation of cysteine-type endopeptidase activity	1.69E-03	3.11E-02
GO:0043281	regulation of cysteine-type endopeptidase activity involved in apoptotic process	1.72E-03	3.16E-02
GO:0007165	signal transduction	1.91E-03	3.48E-02
GO:1903322	positive regulation of protein modification by small protein conjugation or removal	1.91E-03	3.48E-02

GO:0042326	negative regulation of phosphorylation	1.98E-03	3.59E-02
GO:0045765	regulation of angiogenesis	1.99E-03	3.60E-02
GO:0022602	ovulation cycle process	2.15E-03	3.82E-02
GO:0003179	heart valve morphogenesis	2.16E-03	3.82E-02
GO:0014015	positive regulation of gliogenesis	2.16E-03	3.82E-02
GO:0060393	regulation of pathway-restricted SMAD protein phosphorylation	2.24E-03	3.95E-02
GO:0050798	activated T cell proliferation	2.32E-03	3.95E-02
GO:1902285	semaphorin-plexin signaling pathway involved in neuron projection guidance	2.32E-03	3.95E-02
GO:0021855	hypothalamus cell migration	2.32E-03	3.95E-02
GO:0046880	regulation of follicle-stimulating hormone secretion	2.32E-03	3.95E-02
GO:1901166	neural crest cell migration involved in autonomic nervous system development	2.32E-03	3.95E-02
GO:0045661	regulation of myoblast differentiation	2.32E-03	3.95E-02
GO:0061314	Notch signaling involved in heart development	2.32E-03	3.95E-02
GO:0015015	heparan sulfate proteoglycan biosynthetic process, enzymatic modification	2.32E-03	3.95E-02
GO:0006189	'de novo' IMP biosynthetic process	2.32E-03	3.95E-02
GO:2000726	negative regulation of cardiac muscle cell differentiation	2.32E-03	3.95E-02
GO:0048714	positive regulation of oligodendrocyte differentiation	2.32E-03	3.95E-02
GO:0002043	blood vessel endothelial cell proliferation involved in sprouting angiogenesis	2.32E-03	3.95E-02
GO:0033151	V(D)J recombination	2.35E-03	3.95E-02
GO:0021675	nerve development	2.35E-03	3.95E-02
GO:0042100	B cell proliferation	2.35E-03	3.95E-02
GO:0045577	regulation of B cell differentiation	2.35E-03	3.95E-02
GO:0001934	positive regulation of protein phosphorylation	2.39E-03	4.00E-02
GO:0043409	negative regulation of MAPK cascade	2.41E-03	4.01E-02
GO:0030099	myeloid cell differentiation	2.41E-03	4.01E-02
GO:0000796	condensin complex	2.42E-03	4.02E-02
GO:0034770	histone H4-K20 methylation	2.50E-03	4.02E-02
GO:0021517	ventral spinal cord development	2.50E-03	4.02E-02
GO:0007221	positive regulation of transcription of Notch receptor target	2.50E-03	4.02E-02
GO:0034091	regulation of maintenance of sister chromatid cohesion	2.50E-03	4.02E-02
GO:0003184	pulmonary valve morphogenesis	2.50E-03	4.02E-02
GO:0021800	cerebral cortex tangential migration	2.50E-03	4.02E-02
GO:0048617	embryonic foregut morphogenesis	2.50E-03	4.02E-02
GO:0034182	regulation of maintenance of mitotic sister chromatid cohesion	2.50E-03	4.02E-02
GO:0003149	membranous septum morphogenesis	2.50E-03	4.02E-02
GO:0032276	regulation of gonadotropin secretion	2.50E-03	4.02E-02
GO:0042325	regulation of phosphorylation	2.53E-03	4.06E-02
GO:0001933	negative regulation of protein phosphorylation	2.63E-03	4.21E-02
GO:0005021	vascular endothelial growth factor-activated receptor activity	2.70E-03	4.30E-02
GO:0048511	rhythmic process	2.70E-03	4.30E-02
GO:0042327	positive regulation of phosphorylation	2.71E-03	4.30E-02
GO:1905114	cell surface receptor signaling pathway involved in cell-cell signaling	2.73E-03	4.32E-02
GO:0001932	regulation of protein phosphorylation	2.84E-03	4.47E-02
GO:0055006	cardiac cell development	2.92E-03	4.56E-02
GO:0010719	negative regulation of epithelial to mesenchymal transition	2.92E-03	4.56E-02
GO:0008585	female gonad development	2.92E-03	4.56E-02
GO:0035265	organ growth	2.92E-03	4.56E-02
GO:0001756	somitogenesis	3.21E-03	4.94E-02

Table S24. Strigiformes specifically enriched KEGG pathways of genes in the highly conserved genomic regions (HCRs). *P*-value was calculated by Fisher's exact test.

KEGG	Description	*P*-value	FDR
ko05210	Colorectal cancer	1.01E-03	1.85E-02
ko01522	Endocrine resistance	3.03E-03	4.00E-02
ko05215	Prostate cancer	1.19E-03	2.00E-02
ko05213	Endometrial cancer	1.95E-04	4.59E-03
ko04510	Focal adhesion	8.97E-04	1.85E-02
ko04213	Longevity regulating pathway - multiple species	3.91E-03	4.76E-02
ko00534	Glycosaminoglycan biosynthesis - heparan sulfate / heparin	1.13E-04	3.00E-03
ko04916	Melanogenesis	6.97E-04	1.54E-02
ko04391	Hippo signaling pathway - fly	2.77E-03	3.94E-02
ko04520	Adherens junction	4.17E-05	1.37E-03

Table S25. Accipitriformes specific GO enrichment of genes in the highly conserved genomic regions (HCRs). *P*-value was calculated by Fisher's exact test.

GO term	Description	*P*-value	FDR
GO:0048813	dendrite morphogenesis	8.55E-05	4.67E-03
GO:0060976	coronary vasculature development	1.15E-04	5.95E-03
GO:0005515	protein binding	1.70E-04	8.36E-03
GO:0019321	pentose metabolic process	2.45E-04	1.15E-02
GO:0046548	retinal rod cell development	2.45E-04	1.15E-02
GO:0009755	hormone-mediated signaling pathway	3.39E-04	1.54E-02
GO:0050890	cognition	3.40E-04	1.54E-02
GO:0007611	learning or memory	3.43E-04	1.54E-02
GO:0071840	cellular component organization or biogenesis	3.52E-04	1.57E-02
GO:0005930	axoneme	3.54E-04	1.57E-02
GO:0043167	ion binding	3.64E-04	1.61E-02
GO:0046040	IMP metabolic process	3.83E-04	1.66E-02
GO:0006188	IMP biosynthetic process	3.83E-04	1.66E-02
GO:0016043	cellular component organization	4.21E-04	1.78E-02
GO:0030030	cell projection organization	7.09E-04	2.78E-02
GO:0051056	regulation of small GTPase mediated signal transduction	9.69E-04	3.65E-02
GO:0014069	postsynaptic density	1.22E-03	4.37E-02
GO:0099572	postsynaptic specialization	1.22E-03	4.37E-02
GO:0042462	eye photoreceptor cell development	1.25E-03	4.43E-02
GO:0042461	photoreceptor cell development	1.25E-03	4.43E-02
GO:0009168	purine ribonucleoside monophosphate biosynthetic process	1.30E-03	4.57E-02
GO:0061549	sympathetic ganglion development	1.35E-03	4.61E-02
GO:0030323	respiratory tube development	1.35E-03	4.61E-02
GO:0044030	regulation of DNA methylation	1.35E-03	4.61E-02
GO:0043101	purine-containing compound salvage	1.35E-03	4.61E-02
GO:0043401	steroid hormone mediated signaling pathway	1.45E-03	4.79E-02

Table S26. Accipitriformes specifically enriched KEGG pathways of genes in the highly conserved genomic regions (HCRs). *P*-value was calculated by Fisher's exact test.

KEGG	Description	*P*-value	FDR
ko05220	Chronic myeloid leukemia	7.94E-04	1.60E-02
ko04152	AMPK signaling pathway	2.00E-03	3.54E-02
ko00280	Valine, leucine and isoleucine degradation	2.29E-03	3.86E-02
ko05211	Renal cell carcinoma	2.58E-03	4.15E-02

Table S27. Falconiformes specific GO enrichment of genes in the highly conserved genomic regions (HCRs). *P*-value was calculated by Fisher's exact test. Only GO categories with *P* < 1.00E-03 are shown.

GO term	Description	*P*-value	FDR
GO:0051216	cartilage development	2.27E-08	1.01E-06
GO:0048863	stem cell differentiation	2.90E-07	1.12E-05
GO:0040008	regulation of growth	6.86E-07	2.49E-05
GO:0003156	regulation of animal organ formation	7.88E-07	2.82E-05
GO:2000826	regulation of heart morphogenesis	1.14E-06	3.99E-05
GO:2001053	regulation of mesenchymal cell apoptotic process	1.91E-06	6.55E-05
GO:0030856	regulation of epithelial cell differentiation	2.78E-06	9.14E-05
GO:0048864	stem cell development	4.20E-06	1.33E-04
GO:0014032	neural crest cell development	8.55E-06	2.56E-04
GO:0048665	neuron fate specification	8.55E-06	2.56E-04
GO:0031128	developmental induction	9.87E-06	2.90E-04
GO:2000736	regulation of stem cell differentiation	9.99E-06	2.92E-04
GO:0060688	regulation of morphogenesis of a branching structure	1.18E-05	3.39E-04
GO:0001667	ameboidal-type cell migration	1.40E-05	3.91E-04
GO:2000738	positive regulation of stem cell differentiation	1.42E-05	3.95E-04
GO:0048638	regulation of developmental growth	1.86E-05	5.12E-04
GO:0051149	positive regulation of muscle cell differentiation	2.00E-05	5.48E-04
GO:0010464	regulation of mesenchymal cell proliferation	3.01E-05	7.86E-04
GO:0009791	post-embryonic development	3.07E-05	7.96E-04
GO:0021915	neural tube development	3.22E-05	8.23E-04
GO:0007507	heart development	4.16E-05	1.04E-03
GO:1903706	regulation of hemopoiesis	4.54E-05	1.11E-03
GO:0048557	embryonic digestive tract morphogenesis	4.57E-05	1.11E-03
GO:2000136	regulation of cell proliferation involved in heart morphogenesis	4.57E-05	1.11E-03
GO:0072079	nephron tubule formation	4.57E-05	1.11E-03
GO:0030857	negative regulation of epithelial cell differentiation	4.95E-05	1.19E-03
GO:0048806	genitalia development	4.95E-05	1.19E-03
GO:2001054	negative regulation of mesenchymal cell apoptotic process	5.15E-05	1.23E-03
GO:2000177	regulation of neural precursor cell proliferation	6.91E-05	1.62E-03
GO:0002053	positive regulation of mesenchymal cell proliferation	6.95E-05	1.62E-03
GO:1901360	organic cyclic compound metabolic process	7.35E-05	1.69E-03
GO:1905209	positive regulation of cardiocyte differentiation	8.15E-05	1.83E-03
GO:0061217	regulation of mesonephros development	8.15E-05	1.83E-03
GO:1901215	negative regulation of neuron death	8.37E-05	1.87E-03
GO:0046622	positive regulation of organ growth	9.07E-05	1.99E-03
GO:0071407	cellular response to organic cyclic compound	1.07E-04	2.33E-03
GO:0002063	chondrocyte development	1.12E-04	2.43E-03
GO:0048048	embryonic eye morphogenesis	1.12E-04	2.43E-03
GO:0046620	regulation of organ growth	1.15E-04	2.48E-03
GO:0010634	positive regulation of epithelial cell migration	1.20E-04	2.56E-03
GO:0050680	negative regulation of epithelial cell proliferation	1.20E-04	2.56E-03
GO:0048103	somatic stem cell division	1.23E-04	2.59E-03
GO:0036003	positive regulation of transcription from RNA polymerase II promoter in response to stress	1.23E-04	2.59E-03
GO:2000696	regulation of epithelial cell differentiation involved in kidney development	1.23E-04	2.59E-03
GO:1905331	negative regulation of morphogenesis of an epithelium	1.23E-04	2.59E-03
GO:0009798	axis specification	1.24E-04	2.59E-03
GO:0048839	inner ear development	1.35E-04	2.81E-03
GO:0048872	homeostasis of number of cells	1.41E-04	2.92E-03
GO:2000243	positive regulation of reproductive process	1.49E-04	3.05E-03
GO:0045926	negative regulation of growth	1.71E-04	3.49E-03
GO:1901213	regulation of transcription from RNA polymerase II promoter involved in heart development	1.80E-04	3.61E-03
GO:0090185	negative regulation of kidney development	1.80E-04	3.61E-03
GO:0021904	dorsal/ventral neural tube patterning	1.80E-04	3.61E-03
GO:0090101	negative regulation of transmembrane receptor protein serine/threonine kinase signaling pathway	1.89E-04	3.77E-03
GO:0060479	lung cell differentiation	2.20E-04	4.33E-03
GO:0021954	central nervous system neuron development	2.20E-04	4.33E-03

GO:0090288	negative regulation of cellular response to growth factor stimulus	2.33E-04	4.54E-03
GO:0001837	epithelial to mesenchymal transition	2.41E-04	4.68E-03
GO:0060602	branch elongation of an epithelium	2.47E-04	4.77E-03
GO:0072182	regulation of nephron tubule epithelial cell differentiation	2.47E-04	4.77E-03
GO:0035385	Roundabout signaling pathway	2.67E-04	4.98E-03
GO:0061074	regulation of neural retina development	2.67E-04	4.98E-03
GO:0036302	atrioventricular canal development	2.67E-04	4.98E-03
GO:1902866	regulation of retina development in camera-type eye	2.67E-04	4.98E-03
GO:0021877	forebrain neuron fate commitment	2.67E-04	4.98E-03
GO:0010453	regulation of cell fate commitment	2.85E-04	5.21E-03
GO:0048566	embryonic digestive tract development	2.85E-04	5.21E-03
GO:0060065	uterus development	2.85E-04	5.21E-03
GO:0035909	aorta morphogenesis	2.85E-04	5.21E-03
GO:0048713	regulation of oligodendrocyte differentiation	2.85E-04	5.21E-03
GO:0090189	regulation of branching involved in ureteric bud morphogenesis	2.85E-04	5.21E-03
GO:0042474	middle ear morphogenesis	2.85E-04	5.21E-03
GO:0048565	digestive tract development	3.37E-04	6.04E-03
GO:0014706	striated muscle tissue development	3.49E-04	6.24E-03
GO:0061005	cell differentiation involved in kidney development	3.61E-04	6.39E-03
GO:0072001	renal system development	3.61E-04	6.39E-03
GO:0045992	negative regulation of embryonic development	3.61E-04	6.39E-03
GO:0017145	stem cell division	3.61E-04	6.39E-03
GO:0003007	heart morphogenesis	3.68E-04	6.48E-03
GO:0043542	endothelial cell migration	3.68E-04	6.48E-03
GO:0005102	receptor binding	3.93E-04	6.90E-03
GO:0071772	response to BMP	3.95E-04	6.90E-03
GO:0031369	translation initiation factor binding	4.46E-04	7.63E-03
GO:2001026	regulation of endothelial cell chemotaxis	4.48E-04	7.63E-03
GO:0070168	negative regulation of biomineral tissue development	4.48E-04	7.63E-03
GO:0007399	nervous system development	4.53E-04	7.70E-03
GO:0034641	cellular nitrogen compound metabolic process	5.48E-04	9.20E-03
GO:0030858	positive regulation of epithelial cell differentiation	5.56E-04	9.29E-03
GO:0050867	positive regulation of cell activation	5.61E-04	9.33E-03
GO:0072132	mesenchyme morphogenesis	5.91E-04	9.76E-03
GO:0060487	lung epithelial cell differentiation	6.72E-04	1.09E-02
GO:0021983	pituitary gland development	6.72E-04	1.09E-02
GO:0048546	digestive tract morphogenesis	6.85E-04	1.10E-02
GO:2000179	positive regulation of neural precursor cell proliferation	6.91E-04	1.11E-02
GO:0060349	bone morphogenesis	7.00E-04	1.12E-02
GO:0008544	epidermis development	7.00E-04	1.12E-02
GO:0048639	positive regulation of developmental growth	7.68E-04	1.21E-02
GO:0001525	angiogenesis	7.99E-04	1.25E-02
GO:0022029	telencephalon cell migration	9.17E-04	1.41E-02
GO:0072175	epithelial tube formation	9.17E-04	1.41E-02
GO:0001759	organ induction	9.67E-04	1.46E-02
GO:1905276	regulation of epithelial tube formation	9.67E-04	1.46E-02
GO:0048596	embryonic camera-type eye morphogenesis	9.67E-04	1.46E-02
GO:0060037	pharyngeal system development	9.67E-04	1.46E-02
GO:0021522	spinal cord motor neuron differentiation	9.67E-04	1.46E-02

Table S28. Falconiformes specifically enriched KEGG pathways of genes in the highly conserved genomic regions (HCRs). *P*-value was calculated by Fisher's exact test.

KEGG	Description	*P*-value	FDR
ko04013	MAPK signaling pathway - fly	7.31E-04	1.92E-02
ko04010	MAPK signaling pathway	1.08E-03	2.39E-02

Table S29. Passeriformes specific GO enrichment of genes in the highly conserved genomic regions (HCRs). *P*-value was calculated by Fisher's exact test.

GO term	Description	*P*-value	FDR
GO:0046332	SMAD binding	3.50E-05	2.71E-03
GO:0008038	neuron recognition	3.68E-05	2.83E-03
GO:0044446	intracellular organelle part	2.33E-04	1.50E-02
GO:0071634	regulation of transforming growth factor beta production	3.34E-04	2.03E-02
GO:2001028	positive regulation of endothelial cell chemotaxis	4.92E-04	2.85E-02
GO:0060573	cell fate specification involved in pattern specification	4.92E-04	2.85E-02
GO:0044422	organelle part	7.32E-04	4.02E-02
GO:0090575	RNA polymerase II transcription factor complex	8.86E-04	4.79E-02

Table S30. Commonly enriched Gene Ontology (GO) categories of expanded gene families in the common ancestor of Strigiformes and brown kiwi. *P*-value was calculated by Fisher's exact test with a 5% FDR criterion.

GO term	Description	*P*-value in the common ancestor of Strigiformes	*P*-value in brown kiwi
GO:0048869	cellular developmental process	2.01E-05	1.16E-02
GO:0007186	G-protein coupled receptor signaling pathway	8.34E-04	6.44E-03
GO:0060600	dichotomous subdivision of an epithelial terminal unit	1.60E-03	2.12E-04
GO:0043169	cation binding	2.37E-03	5.96E-03
GO:0046872	metal ion binding	3.00E-03	1.16E-02
GO:0140097	catalytic activity, acting on DNA	3.21E-03	2.74E-02
GO:0004175	endopeptidase activity	3.31E-03	2.77E-02
GO:0050808	synapse organization	3.62E-03	2.90E-02
GO:0008037	cell recognition	4.35E-03	1.53E-03
GO:0007411	axon guidance	4.52E-03	5.98E-03
GO:0097485	neuron projection guidance	4.80E-03	6.15E-03
GO:0010954	positive regulation of protein processing	5.78E-03	1.09E-03
GO:0043542	endothelial cell migration	7.46E-03	9.21E-03
GO:1903319	positive regulation of protein maturation	8.29E-03	1.45E-03
GO:0034061	DNA polymerase activity	8.95E-03	7.69E-03
GO:0106030	neuron projection fasciculation	9.22E-03	1.09E-03
GO:0007413	axonal fasciculation	9.22E-03	1.09E-03
GO:0001764	neuron migration	9.35E-03	4.91E-02
GO:0070613	regulation of protein processing	9.61E-03	1.28E-02
GO:1903317	regulation of protein maturation	1.11E-02	1.39E-02
GO:0045095	keratin filament	1.15E-02	1.15E-03
GO:0071897	DNA biosynthetic process	1.71E-02	2.00E-02
GO:0007606	sensory perception of chemical stimulus	1.92E-02	1.48E-03
GO:0008038	neuron recognition	1.95E-02	2.10E-03
GO:0004888	transmembrane signaling receptor activity	2.13E-02	5.75E-03
GO:0060089	molecular transducer activity	2.29E-02	1.16E-02
GO:0002039	p53 binding	2.32E-02	2.79E-02
GO:0051606	detection of stimulus	2.54E-02	7.33E-03
GO:0001843	neural tube closure	2.78E-02	2.48E-02
GO:0099600	transmembrane receptor activity	2.84E-02	7.18E-03
GO:0060606	tube closure	2.95E-02	2.55E-02
GO:0050906	detection of stimulus involved in sensory perception	3.04E-02	1.85E-03
GO:0004872	receptor activity	3.66E-02	9.68E-03
GO:0004930	G-protein coupled receptor activity	4.20E-02	3.06E-03
GO:0007608	sensory perception of smell	4.58E-02	7.17E-04
GO:0004871	signal transducer activity	4.67E-02	1.26E-02
GO:0008270	zinc ion binding	4.89E-02	4.40E-03
GO:0050907	detection of chemical stimulus involved in sensory perception	4.91E-02	5.99E-04
GO:0004553	hydrolase activity, hydrolyzing O-glycosyl compounds	4.93E-02	4.21E-03

Table S31. Commonly enriched Gene Ontology (GO) categories of contracted gene families in the common ancestor of Strigiformes and brown kiwi. *P*-value was calculated by Fisher's exact test with a 5% FDR criterion.

GO term	Description	*P*-value in the common ancestor of Strigiformes	*P*-value in brown kiwi
GO:0044815	DNA packaging complex	4.75E-02	2.40E-02
GO:0000786	nucleosome	3.53E-02	1.66E-02
GO:0005200	structural constituent of cytoskeleton	1.03E-02	1.71E-06
GO:0005198	structural molecule activity	1.96E-02	1.15E-02
GO:0005882	intermediate filament	4.34E-02	2.37E-04

Table S32. Gene Ontology (GO) enrichment of gene families that were expanded in size the present nocturnal bird species genomes. *P*-value was calculated by Fisher's exact test with a 5% FDR criterion.

GO term	Description	Fold-change of Avg. gene number	*P*-value
GO:0014069	postsynaptic density	1.52	2.00E-06
GO:0099572	postsynaptic specialization	1.52	2.00E-06
GO:0043167	ion binding	2.11	1.75E-05
GO:0044459	plasma membrane part	1.63	2.38E-05
GO:0044456	synapse part	1.58	8.51E-05
GO:0045202	synapse	1.61	1.51E-04
GO:0046872	metal ion binding	2.22	1.71E-04
GO:0043169	cation binding	2.24	1.75E-04
GO:0097676	histone H3-K36 dimethylation	1.84	1.92E-04
GO:0071415	cellular response to purine-containing compound	2.10	2.45E-04
GO:0034765	regulation of ion transmembrane transport	1.66	2.77E-04
GO:0016043	cellular component organization	1.77	3.08E-04
GO:0007635	chemosensory behavior	1.53	3.21E-04
GO:0030054	cell junction	1.52	3.23E-04
GO:0050808	synapse organization	1.55	3.24E-04
GO:0004714	transmembrane receptor protein tyrosine kinase activity	1.94	3.75E-04
GO:0070062	extracellular exosome	1.66	4.03E-04
GO:0034762	regulation of transmembrane transport	1.66	4.76E-04
GO:0040011	locomotion	1.63	4.79E-04
GO:1903561	extracellular vesicle	1.66	5.40E-04
GO:0043230	extracellular organelle	1.66	5.46E-04
GO:0071840	cellular component organization or biogenesis	1.77	5.56E-04
GO:0043235	receptor complex	1.70	5.70E-04
GO:0022604	regulation of cell morphogenesis	1.55	6.71E-04

Table S33. Gene Ontology (GO) enrichment of gene families that were contracted in size the present nocturnal bird species genomes. *P*-value was calculated by Fisher's exact test with a 5% FDR criterion.

GO term	Description	Fold-change of Avg. gene number	*P*-value
GO:0045321	aminopeptidase activity	0.59	5.63E-05
GO:0008121	ubiquinol-cytochrome-c reductase activity	0.39	1.02E-04
GO:0016679	oxidoreductase activity, acting on diphenols and related substances as donors	0.39	1.02E-04
GO:0005675	holo TFIIH complex	0.38	1.35E-04
GO:0046985	positive regulation of hemoglobin biosynthetic process	0.58	3.18E-04
GO:0070985	TFIIK complex	0.38	4.00E-04
GO:0006366	transcription from RNA polymerase II promoter	0.55	4.14E-04
GO:0001664	G-protein coupled receptor binding	0.59	5.37E-04
GO:1903747	regulation of establishment of protein localization to mitochondrion	0.52	5.91E-04
GO:0046984	regulation of hemoglobin biosynthetic process	0.58	9.42E-04
GO:0006357	regulation of transcription from RNA polymerase II promoter	0.53	1.08E-03
GO:0043226	organelle	0.53	1.15E-03
GO:0051049	regulation of transport	0.59	1.31E-03
GO:0004177	aminopeptidase activity	0.59	1.33E-03
GO:1903749	positive regulation of establishment of protein localization to mitochondrion	0.49	1.39E-03
GO:0046579	positive regulation of Ras protein signal transduction	0.39	1.46E-03
GO:0048585	negative regulation of response to stimulus	0.59	1.66E-03
GO:0048518	positive regulation of biological process	0.58	1.81E-03
GO:0010314	phosphatidylinositol-5-phosphate binding	0.59	2.06E-03
GO:0032806	carboxy-terminal domain protein kinase complex	0.38	2.22E-03
GO:0007517	muscle organ development	0.56	2.30E-03
GO:0008283	cell proliferation	0.57	2.41E-03
GO:0002521	leukocyte differentiation	0.53	2.43E-03
GO:0003690	double-stranded DNA binding	0.54	2.50E-03
GO:0010035	response to inorganic substance	0.59	2.53E-03
GO:0051057	positive regulation of small GTPase mediated signal transduction	0.39	2.72E-03
GO:1990837	sequence-specific double-stranded DNA binding	0.54	2.82E-03
GO:0044282	small molecule catabolic process	0.58	2.84E-03
GO:0019752	carboxylic acid metabolic process	0.58	2.88E-03
GO:0001067	regulatory region nucleic acid binding	0.56	2.90E-03
GO:0001817	regulation of cytokine production	0.59	2.92E-03
GO:0061575	cyclin-dependent protein serine/threonine kinase activator activity	0.39	2.94E-03
GO:0031077	post-embryonic camera-type eye development	0.51	3.07E-03
GO:0001819	positive regulation of cytokine production	0.56	3.10E-03
GO:0043484	regulation of RNA splicing	0.57	3.39E-03
GO:0016054	organic acid catabolic process	0.56	3.42E-03
GO:0046395	carboxylic acid catabolic process	0.56	3.42E-03
GO:0043227	membrane-bounded organelle	0.54	3.52E-03
GO:0048522	positive regulation of cellular process	0.59	3.58E-03
GO:0000976	transcription regulatory region sequence-specific DNA binding	0.55	3.75E-03
GO:0002724	regulation of T cell cytokine production	0.47	3.77E-03
GO:0070493	thrombin-activated receptor signaling pathway	0.34	3.77E-03
GO:0080134	regulation of response to stress	0.54	3.84E-03
GO:0002699	positive regulation of immune effector process	0.48	3.87E-03
GO:1903955	positive regulation of protein targeting to mitochondrion	0.49	4.16E-03
GO:0010821	regulation of mitochondrion organization	0.57	4.18E-03
GO:0061061	muscle structure development	0.56	4.31E-03
GO:0000900	translation repressor activity, nucleic acid binding	0.29	4.36E-03
GO:0030217	T cell differentiation	0.55	4.39E-03
GO:0010822	positive regulation of mitochondrion organization	0.53	4.77E-03
GO:0030098	lymphocyte differentiation	0.55	4.80E-03
GO:0030317	flagellated sperm motility	0.59	4.96E-03
GO:0045737	positive regulation of cyclin-dependent protein serine/threonine kinase activity	0.38	5.02E-03
GO:0007602	phototransduction	0.53	6.01E-03
GO:0009583	detection of light stimulus	0.52	8.78E-03

Table S34. Gene Ontology (GO) enrichment of PSGs that were shared in two and/or three nocturnal bird groups. *P*-value was calculated by Fisher's exact test.

GO term	Description	*P*-value	*FDR*
GO:1990823	response to leukemia inhibitory factor	3.98E-03	2.67E-01
GO:1990830	cellular response to leukemia inhibitory factor	3.98E-03	2.67E-01
GO:0070129	regulation of mitochondrial translation	5.38E-03	2.67E-01
GO:0008406	gonad development	5.86E-03	2.67E-01
GO:0035025	positive regulation of Rho protein signal transduction	7.94E-03	2.67E-01
GO:0007416	synapse assembly	1.09E-02	2.67E-01
GO:0007283	spermatogenesis	1.43E-02	2.67E-01
GO:0048232	male gamete generation	1.43E-02	2.67E-01
GO:0007098	centrosome cycle	1.44E-02	2.67E-01
GO:0022414	reproductive process	1.72E-02	2.67E-01
GO:0043588	skin development	1.82E-02	2.67E-01
GO:0008584	male gonad development	1.82E-02	2.67E-01
GO:0006614	SRP-dependent cotranslational protein targeting to membrane	1.82E-02	2.67E-01
GO:0007276	gamete generation	1.98E-02	2.67E-01
GO:0034097	response to cytokine	2.08E-02	2.67E-01
GO:0046579	positive regulation of Ras protein signal transduction	2.24E-02	2.67E-01
GO:0006613	cotranslational protein targeting to membrane	2.24E-02	2.67E-01
GO:0051301	cell division	2.30E-02	2.67E-01
GO:0038202	TORC1 signaling	2.39E-02	2.67E-01
GO:0090646	mitochondrial tRNA processing	2.39E-02	2.67E-01
GO:0050832	defense response to fungus	2.39E-02	2.67E-01
GO:0006590	thyroid hormone generation	2.39E-02	2.67E-01
GO:1900483	regulation of protein targeting to vacuolar membrane	2.39E-02	2.67E-01
GO:0042088	T-helper 1 type immune response	2.39E-02	2.67E-01
GO:0033148	positive regulation of intracellular estrogen receptor signaling pathway	2.39E-02	2.67E-01
GO:0030309	poly-N-acetyllactosamine metabolic process	2.39E-02	2.67E-01
GO:0010626	negative regulation of Schwann cell proliferation	2.39E-02	2.67E-01
GO:0010624	regulation of Schwann cell proliferation	2.39E-02	2.67E-01
GO:0043308	eosinophil degranulation	2.39E-02	2.67E-01
GO:0043307	eosinophil activation	2.39E-02	2.67E-01
GO:0032817	regulation of natural killer cell proliferation	2.39E-02	2.67E-01
GO:0032819	positive regulation of natural killer cell proliferation	2.39E-02	2.67E-01
GO:0035493	SNARE complex assembly	2.39E-02	2.67E-01
GO:1902187	negative regulation of viral release from host cell	2.39E-02	2.67E-01
GO:0000964	mitochondrial RNA 5'-end processing	2.39E-02	2.67E-01
GO:0000963	mitochondrial RNA processing	2.39E-02	2.67E-01
GO:0000966	RNA 5'-end processing	2.39E-02	2.67E-01
GO:0051140	regulation of NK T cell proliferation	2.39E-02	2.67E-01
GO:0051142	positive regulation of NK T cell proliferation	2.39E-02	2.67E-01
GO:0045900	negative regulation of translational elongation	2.39E-02	2.67E-01
GO:0032725	positive regulation of granulocyte macrophage colony-stimulating factor production	2.39E-02	2.67E-01
GO:1902713	regulation of interferon-gamma secretion	2.39E-02	2.67E-01
GO:1902715	positive regulation of interferon-gamma secretion	2.39E-02	2.67E-01
GO:0002278	eosinophil activation involved in immune response	2.39E-02	2.67E-01
GO:0016576	histone dephosphorylation	2.39E-02	2.67E-01
GO:0046884	follicle-stimulating hormone secretion	2.39E-02	2.67E-01
GO:0071474	cellular hyperosmotic response	2.39E-02	2.67E-01
GO:0031204	posttranslational protein targeting to membrane, translocation	2.39E-02	2.67E-01
GO:0030311	poly-N-acetyllactosamine biosynthetic process	2.39E-02	2.67E-01
GO:0060253	negative regulation of glial cell proliferation	2.39E-02	2.67E-01
GO:0032645	regulation of granulocyte macrophage colony-stimulating factor production	2.39E-02	2.67E-01
GO:0098928	presynaptic signal transduction	2.39E-02	2.67E-01
GO:0051133	regulation of NK T cell activation	2.39E-02	2.67E-01
GO:0051135	positive regulation of NK T cell activation	2.39E-02	2.67E-01
GO:1903564	regulation of protein localization to cilium	2.39E-02	2.67E-01
GO:0099526	presynapse to nucleus signaling pathway	2.39E-02	2.67E-01
GO:0032740	positive regulation of interleukin-17 production	2.39E-02	2.67E-01
GO:0043520	regulation of myosin II filament assembly	2.39E-02	2.67E-01
GO:1903568	negative regulation of protein localization to ciliary membrane	2.39E-02	2.67E-01

GO:0043519	regulation of myosin II filament organization	2.39E-02	2.67E-01
GO:1903567	regulation of protein localization to ciliary membrane	2.39E-02	2.67E-01
GO:1903565	negative regulation of protein localization to cilium	2.39E-02	2.67E-01
GO:0042060	wound healing	2.69E-02	2.71E-01
GO:0051057	positive regulation of small GTPase mediated signal transduction	2.69E-02	2.71E-01
GO:0007131	reciprocal meiotic recombination	2.69E-02	2.71E-01
GO:0035825	homologous recombination	2.69E-02	2.71E-01
GO:0006310	DNA recombination	2.83E-02	2.73E-01
GO:0098840	protein transport along microtubule	3.18E-02	2.73E-01
GO:0099118	microtubule-based protein transport	3.18E-02	2.73E-01
GO:0072599	establishment of protein localization to endoplasmic reticulum	3.18E-02	2.73E-01
GO:0042073	intraciliary transport	3.18E-02	2.73E-01
GO:0045047	protein targeting to ER	3.18E-02	2.73E-01
GO:0048609	multicellular organismal reproductive process	3.22E-02	2.73E-01
GO:0035023	regulation of Rho protein signal transduction	3.35E-02	2.73E-01
GO:0042102	positive regulation of T cell proliferation	3.71E-02	2.73E-01
GO:0071345	cellular response to cytokine stimulus	4.09E-02	2.73E-01
GO:0051225	spindle assembly	4.26E-02	2.73E-01
GO:0070972	protein localization to endoplasmic reticulum	4.26E-02	2.73E-01
GO:0031023	microtubule organizing center organization	4.26E-02	2.73E-01
GO:1903046	meiotic cell cycle process	4.28E-02	2.73E-01
GO:0048608	reproductive structure development	4.28E-02	2.73E-01
GO:0006972	hyperosmotic response	4.72E-02	2.73E-01
GO:0032926	negative regulation of activin receptor signaling pathway	4.72E-02	2.73E-01
GO:1900060	negative regulation of ceramide biosynthetic process	4.72E-02	2.73E-01
GO:0032729	positive regulation of interferon-gamma production	4.72E-02	2.73E-01
GO:0097286	iron ion import	4.72E-02	2.73E-01
GO:1902525	regulation of protein monoubiquitination	4.72E-02	2.73E-01
GO:0043306	positive regulation of mast cell degranulation	4.72E-02	2.73E-01
GO:0002888	positive regulation of myeloid leukocyte mediated immunity	4.72E-02	2.73E-01
GO:0051968	positive regulation of synaptic transmission, glutamatergic	4.72E-02	2.73E-01
GO:0032660	regulation of interleukin-17 production	4.72E-02	2.73E-01
GO:1902916	positive regulation of protein polyubiquitination	4.72E-02	2.73E-01
GO:0051382	kinetochore assembly	4.72E-02	2.73E-01
GO:1903595	positive regulation of histamine secretion by mast cell	4.72E-02	2.73E-01
GO:0035418	protein localization to synapse	4.72E-02	2.73E-01
GO:0045176	apical protein localization	4.72E-02	2.73E-01
GO:1903593	regulation of histamine secretion by mast cell	4.72E-02	2.73E-01
GO:0050910	detection of mechanical stimulus involved in sensory perception of sound	4.72E-02	2.73E-01
GO:1905342	positive regulation of protein localization to kinetochore	4.72E-02	2.73E-01
GO:0032925	regulation of activin receptor signaling pathway	4.72E-02	2.73E-01
GO:0060005	vestibular reflex	4.72E-02	2.73E-01
GO:0032274	gonadotropin secretion	4.72E-02	2.73E-01
GO:0033008	positive regulation of mast cell activation involved in immune response	4.72E-02	2.73E-01
GO:0033692	cellular polysaccharide biosynthetic process	4.72E-02	2.73E-01
GO:1904424	regulation of GTP binding	4.72E-02	2.73E-01
GO:0035655	interleukin-18-mediated signaling pathway	4.72E-02	2.73E-01
GO:0014902	myotube differentiation	4.72E-02	2.73E-01
GO:0046597	negative regulation of viral entry into host cell	4.72E-02	2.73E-01
GO:0002323	natural killer cell activation involved in immune response	4.72E-02	2.73E-01
GO:0090155	negative regulation of sphingolipid biosynthetic process	4.72E-02	2.73E-01
GO:0090156	cellular sphingolipid homeostasis	4.72E-02	2.73E-01
GO:0046641	positive regulation of alpha-beta T cell proliferation	4.72E-02	2.73E-01
GO:0043320	natural killer cell degranulation	4.72E-02	2.73E-01
GO:1902914	regulation of protein polyubiquitination	4.72E-02	2.73E-01
GO:1905340	regulation of protein localization to kinetochore	4.72E-02	2.73E-01
GO:0032275	luteinizing hormone secretion	4.72E-02	2.73E-01
GO:0070131	positive regulation of mitochondrial translation	4.72E-02	2.73E-01
GO:1903335	regulation of vacuolar transport	4.72E-02	2.73E-01
GO:0006620	posttranslational protein targeting to endoplasmic reticulum membrane	4.72E-02	2.73E-01
GO:0002573	myeloid leukocyte differentiation	4.84E-02	2.73E-01

Table S35. List of genes showing accelerated d_N/d_S in the nocturnal birds

Gene	ω Nocturnal birds branches				ω outgroups
	The ancestral branch of Strigiformes	Chuck-will's-widow	Brown kiwi	Merged	
RDH8	0.4098	0.2631	0.4149	0.3573	0.0630
PDE6B	0.2270	0.2128	0.1369	0.1720	0.0579
LOC100858777	0.5158	0.2636	0.6814	0.4287	0.1009
PPEF2	0.2939	0.3382	0.2188	0.2936	0.1081
SLC24A1	1.8907	0.7179	0.6050	0.7305	0.2656
LOC770429	1.8810	0.4821	0.4012	0.5092	0.1971
ACTN1	0.4484	0.0408	0.0320	0.1136	0.0226
CPLX4	2.0519	1.5421	0.1849	0.5086	0.0671
GRK7	1.2989	0.7244	0.2820	0.5997	0.2282
GBE	0.5387	0.6466	2.3148	0.7049	0.1294
ENO2	0.2890	0.0457	0.0976	0.0833	0.0080
MUM1	1.3949	0.8561	0.4759	0.7825	0.3691
BEND3	0.0906	0.1107	0.6125	0.2253	0.0757
KCNIP2	0.0627	0.1542	0.0286	0.0945	0.0177
PDC	4.0970	0.2920	0.5936	0.4637	0.1031
RUNDC3B	0.4267	0.3511	0.1407	0.3266	0.0697
TMEM136-2	0.8141	0.3970	0.2174	0.4389	0.0890
RHO	0.0620	0.1328	0.1149	0.1197	0.0431
RRH	0.4084	0.2198	0.4350	0.3110	0.1312
CRYAA	0.0935	0.3236	0.1191	0.2035	0.0460
FAM83A	0.2657	0.2274	0.3965	0.3106	0.1495
RLBP1	0.1033	0.0674	0.1733	0.1110	0.0351
ZNF407	1.2569	0.6139	0.5760	0.6260	0.3920
SGCG	0.0752	0.1392	0.2843	0.1695	0.0306
CEP55	2.1652	1.0990	0.4801	0.9708	0.4224
CASR	0.0434	0.0661	0.0999	0.0766	0.0289
FKBP6	0.9669	0.6302	0.4384	0.7471	0.3342
CHST10	0.1899	0.2257	0.4136	0.2634	0.0656
PDLIM3	1.1664	0.2484	0.4213	0.3938	0.1494
ADRA1A	0.2044	0.1836	0.4901	0.2776	0.0884
GPR146	0.2949	0.2438	0.4403	0.2995	0.0568
FHL1	0.7873	0.1589	0.1746	0.2654	0.0652
RP11-463C8.4	0.4798	0.7896	0.6159	0.6598	0.2637
RFXANK	1.0069	0.3856	1.3770	0.7037	0.1394
DRD2	0.1987	0.1552	0.9198	0.2854	0.1019
NPHP3	0.1000	0.1792	0.1727	0.1714	0.0884
AAK1	0.1670	0.4417	0.1812	0.2513	0.1322
PRICKLE1	0.1153	0.0451	0.1724	0.0961	0.0375
KIF26B	0.1321	0.1685	0.2146	0.1836	0.1123
CALHM2	0.0371	0.0694	0.1310	0.0838	0.0294
HSPA2	0.0830	0.0681	0.0162	0.0455	0.0131
BEGAIN	0.1063	0.2151	0.2644	0.2021	0.0875
C3H2orf71	1.4416	0.6548	0.4084	0.5798	0.3960
GAS7	0.1300	0.2561	0.1441	0.2105	0.0705
RPE65	0.1248	0.0339	0.1030	0.0692	0.0310
ARL6	0.8677	0.2652	0.6592	0.3914	0.0567
LRRN3	0.3543	0.1801	0.3305	0.2472	0.1234
TSNAX	0.3182	0.3185	0.2394	0.2957	0.0851
SPNS3	1.2045	0.5252	0.6965	0.5962	0.3011
OPN4-1	0.5534	0.4355	0.4605	0.4597	0.2325
DCLK3	0.3268	0.3693	0.6218	0.4242	0.2054
FBXO21	1.3829	0.1001	0.2019	0.1695	0.0516
LOC428319	1.1610	0.1940	0.7668	0.3171	0.1562
C1H12ORF4	0.1800	0.1460	0.1880	0.1647	0.0609
BEST1	0.2125	0.1894	0.1408	0.1806	0.1041
GSG1L2	0.9560	0.4434	0.5273	0.5901	0.2750
ACER1	0.4939	0.5089	0.2949	0.4532	0.1643
SHANK2	0.1780	0.0744	0.1410	0.1268	0.0629
CCDC51	1.4425	0.3465	0.5441	0.4442	0.2130

TRMT10A	0.3208	2.2641	0.6503	0.7142	0.2537
G6PC2	0.1540	0.5672	0.8092	0.4531	0.1321
LOC107052104	0.6353	0.6346	0.3803	0.5589	0.3019
QSOX2	0.2591	0.3615	0.3344	0.3289	0.1679
TFEC	0.3179	0.5371	0.2269	0.3406	0.1057
GPRIN2	1.3676	0.7682	0.5216	0.6787	0.4014
TOX	0.6672	0.3162	0.2454	0.2865	0.0838
STX18	0.9082	0.4426	0.2132	0.3017	0.0945
HSF3	0.7621	0.2831	0.7257	0.3688	0.1769
INPP5J	0.1166	0.1781	0.2149	0.1717	0.0954
TEKT1	0.8477	0.9842	0.6502	0.8270	0.3987
CSE1L	0.0598	0.0506	0.0378	0.0481	0.0153
MDM4	0.7803	0.5120	0.2855	0.4364	0.1861
MYOT	0.7442	0.2031	0.2157	0.2403	0.1066
NOS2	0.8812	0.3062	0.3670	0.3453	0.2163

Table S36. Olfactory receptors identified in 25 avian genomes. *P*-value was calculated by Mann-Whitney *U* test, after removing two outlier species, chicken and zebra finch.

Species	Number of intact (functional) OR genes					Number of partial OR genes	Number of pseudogenes
	Total	α	γ all	γ	γ-c		
Eurasian eagle-owl	56	11	45	27	18	94	104
Northern spotted owl	88	11	77	30	47	167	154
Oriental scops-owl	38	9	29	22	7	172	82
Barn owl	30	7	23	16	7	67	74
Downy woodpecker	19	0	19	16	3	36	33
Golden eagle	39	9	30	25	5	55	73
Eastern buzzard	38	9	29	19	10	48	66
Bald eagle	40	10	30	20	10	40	66
Zebra finch	167	1	166	3	163	284	377
American crow	13	4	9	8	1	26	32
Budgerigar	26	2	24	23	1	45	38
Saker falcon	25	7	18	14	4	24	51
Peregrine falcon	25	8	17	14	3	25	49
Common kestrel	25	10	15	14	1	22	42
Little egret	90	7	83	18	65	120	166
Crested ibis	33	5	28	24	4	55	74
Hoatzin	53	9	44	24	20	84	126
Killdeer	49	10	39	32	7	59	86
Chuck-will's-widow	34	6	28	26	2	54	58
Anna's hummingbird	23	2	21	17	4	72	85
Common cuckoo	29	7	22	17	5	38	53
Rock dove	36	7	29	18	11	122	112
Chicken	279	7	272	40	232	426	539
Common ostrich	53	22	31	26	5	27	71
Brown kiwi	53	15	38	30	8	63	101
P-value (nocturnal vs. diurnal)	0.053	0.079	0.080	**0.027**	0.11	-	-
P-value (owls vs. others)	0.084	0.095	0.100	0.18	**0.040**	-	-

Table S37. The diversity of olfactory receptors in 25 avian genomes. *P*-value was calculated by Mann-Whitney *U* test.

Species	Diversity of OR genes by Shannon entropy			
	α	γ all	γ	γ-c
Eurasian eagle-owl	1.001	1.305	1.249	0.610
Northern spotted owl	0.978	1.076	1.265	0.478
Oriental scops-owl	0.895	1.178	1.142	0.432
Barn owl	0.884	1.267	1.125	0.773
Downy woodpecker	-	1.169	1.148	0.174
Golden eagle	0.862	1.247	1.218	0.317
Eastern buzzard	0.847	1.156	1.143	0.285
Bald eagle	0.897	1.159	1.139	0.357
Zebra finch	-	0.485	0.651	0.433
American crow	0.682	1.098	0.998	-
Budgerigar	0.350	0.989	0.947	-
Saker falcon	0.918	1.193	1.061	0.521
Peregrine falcon	0.855	1.143	1.062	0.236
Common kestrel	0.873	1.084	1.055	-
Little egret	0.761	0.954	1.150	0.624
Crested ibis	0.731	1.361	1.312	0.492
Hoatzin	0.898	1.310	1.281	0.680
Killdeer	0.965	1.307	1.277	0.448
Chuck-will's-widow	0.823	1.237	1.188	0.323
Anna's hummingbird	0.379	1.204	1.144	0.274
Common cuckoo	0.845	1.247	1.223	0.256
Rock dove	0.826	1.273	1.142	0.618
Chicken	0.803	0.866	1.081	0.600
Common ostrich	1.037	1.219	1.169	0.416
Brown kiwi	1.045	1.267	1.151	0.620
P-value (nocturnal vs. diurnal)	**0.027**	0.16	0.13	0.086
P-value (owls vs. others)	**0.041**	0.34	0.24	0.094

Table S38. Sensory system associated genes showing accelerated d_N/d_S in the nocturnal birds

Associated sensory system	Gene	ω Three nocturnal branches				ω outgroups
		The ancestral branch of Strigiformes	Chuck-will's-widow	Brown kiwi	Merged	
Hearing	*ATG5*	0.1894	0.0971	0.0854	0.1051	0.0627
Hearing	*ATP8B1*	0.1148	0.1124	0.1165	0.1142	0.0993
Hearing	*CDH2*	0.1848	0.0898	0.0644	0.0849	0.0416
Hearing	*CEMIP*	0.1241	0.1136	0.1311	0.1214	0.0907
Hearing	*CUX1*	0.2219	0.0996	0.0738	0.0891	0.0677
Hearing	*DFNB59*	0.0979	0.0961	0.1630	0.1050	0.0872
Hearing	*DVL3*	3.0003	0.0398	0.0185	0.0248	0.0183
Hearing	*ENTPD2*	0.1328	0.1406	0.1790	0.1609	0.1169
Hearing	*EYA1*	0.1798	0.1716	0.1312	0.1740	0.0259
Hearing	*FAM65B*	0.1754	0.1673	0.1396	0.1566	0.1225
Hearing	*FGF3*	0.6052	0.1124	0.1930	0.1730	0.1061
Hearing	*FREM2*	0.1839	0.1486	0.1488	0.1496	0.1444
Hearing	*GRXCR1*	0.9108	0.1006	0.1889	0.1392	0.0757
Hearing	*LGR5*	0.1291	0.1884	0.2737	0.2186	0.1274
Hearing	*MBP*	0.1625	0.1769	0.2855	0.1977	0.1532
Hearing	*MCOLN3*	0.1917	0.2126	0.1496	0.1889	0.1478
Hearing	*OPA1*	0.1441	0.0704	0.0874	0.0784	0.0561
Hearing	*OTOA*	0.6539	0.4380	0.3596	0.4281	0.3303
Hearing	*OTOGL*	0.6029	0.3451	0.2580	0.2942	0.2554
Hearing	*PTPRQ*	0.3033	0.2292	0.2375	0.2354	0.1941
Hearing	*RAC1*	0.1033	0.3430	0.0275	0.2079	0.0208
Hearing	*SCN8A*	0.0291	0.0687	0.0540	0.0567	0.0162
Hearing	*SCRIB*	0.0782	0.1259	0.1898	0.0979	0.0761
Hearing	*SLC26A2*	0.2770	0.2236	0.2412	0.2346	0.1955
Hearing	*SLITRK6*	0.1920	0.2145	0.1722	0.1987	0.1465
Hearing	*SUPT6H*	0.0885	0.0242	0.0338	0.0334	0.0187
Hearing	*TMEM132E*	0.0570	0.0430	0.0341	0.0412	0.0282
Hearing	*TTC8*	0.1442	0.1771	0.3274	0.1974	0.1363
Hearing	*USH1G*	0.2130	0.0363	0.0614	0.0652	0.0301
Hearing	*USH2A*	0.3548	0.3061	0.3425	0.3377	0.2257
Hearing	*XPC*	0.3814	0.2982	0.5424	0.3752	0.2890
Hearing/Circadian rhythm	*BDNF*	0.3643	0.1203	0.0777	0.1429	0.0373
Hearing/Circadian rhythm	*DRD2*	0.1987	0.1552	0.9198	0.2854	0.1019
Circadian rhythm	*RPE65*	0.1248	0.0339	0.1030	0.0692	0.0310
Circadian rhythm	*ARNTL2*	1.3777	0.1533	0.2053	0.2008	0.1372
Circadian rhythm	*CASP1*	0.3965	0.6995	0.6113	0.6194	0.3946
Circadian rhythm	*DPYD*	0.2093	0.2351	0.2043	0.2171	0.1772
Circadian rhythm	*FBXL21*	0.1398	0.1204	0.2340	0.1404	0.0931
Circadian rhythm	*GSK3B*	78.8389	0.3286	0.3998	0.4242	0.0608
Circadian rhythm	*KCNH7*	0.1898	0.2387	0.1485	0.1890	0.1152
Circadian rhythm	*MAPK10*	0.9519	0.1575	0.1085	0.1469	0.0507
Circadian rhythm	*NOS2*	0.8811	0.3062	0.3670	0.3453	0.2163
Circadian rhythm	*NR1D2*	0.1797	0.2452	0.1391	0.1988	0.1274
Circadian rhythm	*PHLPP1*	0.0568	0.0580	0.1597	0.0965	0.0525
Circadian rhythm	*PMCH*	999	0.6953	0.6341	0.6973	0.4137
Circadian rhythm	*PPARA*	0.4024	0.0610	0.0966	0.0858	0.0373
Circadian rhythm	*PPARGC1A*	0.2171	0.4580	0.2960	0.3532	0.1827
Circadian rhythm	*PRKG2*	0.2854	0.2165	0.3120	0.2688	0.1993
Circadian rhythm	*SLC6A4*	0.5796	0.1080	0.1155	0.1328	0.0888

Supplementary Methods

Genome and transcriptome sequencing

We sequenced the genomes and transcriptomes from 20 avian species (16 birds of prey and four non-raptor birds) using the Illumina HiSeq platforms (HiSeq2000, HiSeq2500, and HiSeq4000) according to the manufacturer's sample preparation protocols. The detailed sequencing platform information for each species and data are shown below. To build reference genome assemblies of the four raptor species, we constructed eleven genomic libraries with different insert sizes (170bp, 500bp, 700bp, 2 Kb, 5 Kb, 10 Kb, and 15 Kb for the Eurasian eagle-owl, oriental scops-owl, and eastern buzzard, 350bp, 550bp, 2 Kb, 5 Kb, 10 Kb, and 15 Kb for common kestrel) for each species.

Species	Common name	Data	Sequencing platform
Bubo bubo	Eurasian eagle-owl	Assembly	HiSeq2500 for short insert libraries, HiSeq2000 for long-mate pair libraries
		Transcriptome	HiSeq2000
Otus sunia	Oriental scops-owl	Assembly	HiSeq2500 for short insert libraries, HiSeq2000 for long-mate pair libraries
		Transcriptome	HiSeq2500
Strix nivicolum	Himalayan owl	Whole genome	HiSeq2000, HiSeq2500
		Transcriptome	HiSeq2000
Ninox japonica	Northern boobook	Whole genome	HiSeq2000, HiSeq2500
		Transcriptome	HiSeq2000
Asio otus	Long-eared owl	Whole genome	HiSeq2000, HiSeq2500
		Transcriptome	HiSeq2000
Asio flammeus	Short-eared owl	Whole genome	HiSeq4000
Otus semitorques	Japanese scops-owl	Whole genome	HiSeq4000
Buteo japonicus	Eastern buzzard	Assembly	HiSeq2500 for short insert libraries, HiSeq2000 for long-mate pair libraries
		Transcriptome	HiSeq2500
Accipiter nisus	Eurasian sparrowhawk	Whole genome	HiSeq2500
		Transcriptome	HiSeq2500
Accipiter gentilis	Northern goshawk	Whole genome	HiSeq2500
		Transcriptome	HiSeq2500
Haliaeetus albicilla	White-tailed eagle	Whole genome	HiSeq4000
Pernis ptilorhynchus	Oriental honey-buzzard	Whole genome	HiSeq4000
Milvus migrans	Black kite	Whole genome	HiSeq4000
Accipiter soloensis	Chinese sparrowhawk	Whole genome	HiSeq4000
Falco tinnunculus	Common kestrel	Assembly	HiSeq2500 for shot-insert and long-mate pair libraries
		Whole genome	HiSeq2500
		Transcriptome	HiSeq2500
Falco subbuteo	Eurasian hobby	Whole genome	HiSeq2500
		Transcriptome	HiSeq2500
Picus canus	Grey-headed woodpecker	Whole genome	HiSeq2000
		Transcriptome	HiSeq2000
Egretta garzetta	Little egret	Whole genome	HiSeq2000
		Transcriptome	HiSeq2500
Butorides striata	Striated heron	Whole genome	HiSeq2000
		Transcriptome	HiSeq2500
Platalea minor	Black-faced spoonbill	Whole genome	HiSeq2000
		Transcriptome	HiSeq2000

Species identification

Species of the sequenced samples were confirmed by mapping their DNA sequences to previously reported mitochondrial sequences (*COI* and *CYTB* genes) for closely related species using BWA-MEM [S18] with default options. Variants were identified using the mpileup command in SAMtools [S19]. The consensus sequences were generated using the vcf2fq command. The *COI* gene of common kestrel was sequenced by Sanger method. Phylogenetic reconstruction was performed using MrBayes 3.2 software [S20] with the "lset=mixed rates=invgamma" substitution model specifications. Species sampling in the phylogeny was designed to include all species from the families Accipitridae, Ardeidae, Falconidae, Picidae, Strigidae, and Threskiornithidae that occur in South Korea. Species that could not be included were *Aviceda leuphotes* (Accipitridae), *Dendrocopos hyperythrus* (Picidae), and *Threskiornis melanocephalus* (Threskiornithidae). In case of the two latter species, congeneric species were included. KM364882 sequence was first attributed to *B. buteo burmanicus*, a junior synonym of *B. refectus*; the sampling locality is outside the known range of *B. refectus* and suggests that it is a misidentified *B. japonicus*. The latter hypothesis is also supported by comparative analyses with the tRNAGlu-Pseudo-control Region sequences [S21].

Sequence filtering criteria

To reduce sequencing error effects in assembling the bird of prey genomes (Eurasian eagle-owl, oriental scops-owl, eastern buzzard, and common kestrel), we filtered out PCR duplicated, low quality, and adaptor contaminated reads. The filtering criteria for exclusion were as follows:

1) Reads were considered PCR duplicates if read1 (left) and read2 (right) of the two paired end reads were identical. The PCR duplicated reads were filtered out remaining one unique read pairs.

2) Reads with sequencing adapter contamination were filtered out.

   Sequencing adapter left= "*GATCGGAAGAGCACACGTCTGAACTCCAGTCAC*"

   Sequencing adapter right= "*GATCGGAAGAGCGTCGTGTAGGGAAAGAGTGT*"

3) Reads with ambiguous base (N) for more than 5% of the reads were filtered out.

4) Reads with an average base quality below 20 (<Q20) were filtered out.

5) Reads with junction adapter contamination for mate-pair libraries were filtered out.

   Junction adapter left = "*CTGTCTCTTATACACATCT*"

   Junction adapter right = "*AGATGTGTATAAGAGACAG*"

6) To filter out low-quality read ends, three bases of 5'-end and eight bases of 3'-end of each read from short insert libraries were trimmed.

7) Each read from long-mate pair libraries were trimmed into 50bp (one base of 3'-end of each read for Eurasian eagle-owl, oriental scops-owl, and eastern buzzard; 51 bases of 3'-end of each read for common kestrel)

Repeat annotation

For the annotation of repetitive elements for the assembled bird of prey genomes, we searched the bird of prey genomes for tandem repeats using the Tandem Repeats Finder (version 4.07b) [S22]. Transposable elements (TEs) were identified in the genomes by homology-based and *ab initio*-based approaches. For the homology-based approach, we identified repeats using Repbase (version 19.03) [23] with RepeatMasker (version 4.0.5) [S24] and RMBlast (version 2.2.28) [S25]. For the *ab initio*-based approach, we used RepeatModeler (version 1.0.7) [S26]. All predicted repetitive elements were merged for statistics by in-house scripts. Roughly ~9.2 % of the bird of prey genomes were predicted as transposable elements, which are similar in composition to the other avian genomes [S2].

Supplementary References

S1. Hanna ZR, Henderson JB, Wall JD, Emerling CA, Fuchs J, Runckel C, et al. Northern Spotted Owl (Strix occidentalis caurina) Genome: Divergence with the Barred Owl (Strix varia) and Characterization of Light-Associated Genes. Genome Biol Evol. 2017;9:2522–2545

S2. Zhang G, Li C, Li Q, Li B, Larkin DM, Lee C, et al. Comparative genomics reveals insights into avian genome evolution and adaptation. Science. 2014;346:1311–1320.

S3. Van Den Bussche RA, Judkins ME, Montague MJ, Warren WC. A Resource of Genome-Wide Single Nucleotide Polymorphisms (Snps) for the Conservation and Management of Golden Eagles. J Raptor Res. 2017;51:368–377.

S4. Chung O, Jin S, Cho YS, Lim J, Kim H, Jho S, et al. The first whole genome and transcriptome of the cinereous vulture reveals adaptation in the gastric and immune defense systems and possible convergent evolution between the Old and New World vultures. Genome Biol. 2015;16:215.

S5. Zhan X, Pan S, Wang J, Dixon A, He J, Muller MG, et al. Peregrine and saker falcon genome sequences provide insights into evolution of a predatory lifestyle. Nat Genet. 2013;45:563–566.

S6. Ganapathy G, Howard JT, Ward JM, Li J, Li B, Li Y, et al. High-coverage sequencing and annotated assemblies of the budgerigar genome. Gigascience. 2014;3:11.

S7. Warren WC, Clayton DF, Ellegren H, Arnold AP, Hillier LW, Künstner A, et al. The genome of a songbird. Nature. 2010;464:757–762.

S8. Videvall E, Cornwallis CK, Palinauskas V, Valkiūnas G, Hellgren O. The avian transcriptome response to malaria infection. Mol Biol Evol. 2015;32:1255–1267.

S9. Shapiro MD, Kronenberg Z, Li C, Domyan ET, Pan H, Campbell M, et al. Genomic diversity and evolution of the head crest in the rock pigeon. Science 2013;339:1063–1067.

S10. International Chicken Genome Sequencing Consortium. Sequence and comparative analysis of the chicken genome provide unique perspectives on vertebrate evolution. Nature. 2004;432:695–716.

S11. Le Duc D, Renaud G, Krishnan A, Almén MS, Huynen L, Prohaska SJ, et al. Kiwi genome provides insights into evolution of a nocturnal lifestyle. Genome Biol. 2015;16:147.

S12. Heimo M. Owls of the World: A Photographic Guide. Richmond Hill: Firefly Books; 2012.

S13. Handbook of the Birds of the World Alive. http://www.hbw.com. Accessed 16 November 2017.

S14. Tacutu R, Craig T, Budovsky A, Wuttke D, Lehmann G, Taranukha D, et al. Human Ageing Genomic Resources: Integrated databases and tools for the biology and genetics of ageing. Nucleic Acids Res. 2013;41:D1027–D1033.

S15. James H, James AK, Robert G, Peter H. The Herons Handbook. London:Bloomsbury Publishing; 2010. p. 175–180.

S16. Müllner A. Breeding ecology and related life-history traits of the Hoatzin, Opisthocomus hoazin, in a primary rainforest habitat. Universität Würzburg. 2004; Dissertation.

S17. Animal Diversity Web. http://animaldiversity.org. Accessed 16 November 2017

S18. Li H. Aligning sequence reads, clone sequences and assembly contigs with BWA-MEM. ArXiv. 2013;1303:3997.

S19. Li H, Handsaker B, Wysoker A, Fennell T, Ruan J, Homer N, et al. The Sequence Alignment/Map format and SAMtools. Bioinformatics. 2009;25:2078–2079.

S20. Ronquist F, Teslenko M, van der Mark P, Ayres DL, Darling A, Höhna S, et al. MrBayes 3.2: efficient Bayesian phylogenetic inference and model choice across a large model space. Syst Biol. 2012;61:539–542.

S21. Kruckenhauser L, Haring E, Pinsker W, Riesing MJ, Winkler H, Wink M, et al. Genetic vs. morphological differentiation of Old World buzzards (genus Buteo, Accipitridae). Zool Scr. 2014;33:197–211.

S22. Benson G. Tandem repeats finder: a program to analyze DNA sequences. Nucleic Acids Res. 1999;27:573–580.

S23. Jurka J, Kapitonov VV, Pavlicek A, Klonowski P, Kohany O, Walichiewicz J. Repbase Update, a database of eukaryotic repetitive elements. Cytogenet Genome Res. 2005;110:462-467.

S24. Bedell JA, Korf I, Gish W. MaskerAid: a performance enhancement to RepeatMasker. Bioinformatics. 2000;16:1040–1041.

S25. RMBlast. http://www.repeatmasker.org/RMBlast.html. Accessed 16 Aug 2016.

S26. Abrusán G, Grundmann N, DeMester L, Makalowski W. TEclass--a tool for automated classification of unknown eukaryotic transposable elements. Bioinformatics. 2009;25:1329–1330.
