# Supplementary material for: Raptor genomes reveal evolutionary signatures of predatory and nocturnal lifestyles
Source: Genome Biol. 2019 Aug 29;20:181. doi: 10.1186/s13059-019-1793-1 (PMC6714440; doi:10.1186/s13059-019-1793-1)
Supplement: Supplementary file 3 — Review history. (DOCX 48 kb) [file 13059_2019_1793_MOESM3_ESM.docx]

Review History

**First round of review**

**Reviewer 1**

**Are you able to assess all statistics in the manuscript, including the appropriateness of statistical tests used?**

No, I do not feel adequately qualified to assess the statistics.

**Comments to author:**

*Please consider the following questions:*

*- Are the methods appropriate to the aims of the study, are they well described, and are necessary controls included? If not, please specify what is required.*

I am happy that the methods are consistent with the aims

*- Are the conclusions adequately supported by the data shown? If not, please explain*

Yes

*- Are sufficient details provided to allow replication and comparison with related analyses that may have been performed? If not, please specify what is required.*

Yes

*- Does the work represent a significant advance over previously published studies?*

Yes

*- Is the paper of broad interest to others in the field, or of outstanding interest to a broad audience of biologists?*

Broad interest to authors in the field

This is paper that describes the sequencing of 3 raptor genomes and identifies signatures of selection with respect to phenotype.

Overall I was happy with this paper however I do find myself somewhat under-qualified to comment on the statistical aspects of the study. For ignoramuses like me therefore I wonder if the authors could spell out a little more clearly (and in places other than the methods) why the results they obtained in terms of over-representation of gene families and specifically when they were talking about the genome, and when the transcriptome - it is not always clear.

Other than that, they may wish to comment on how close their genomes are to chromosome level assemblies and the role of EBRs and HSBs in establishing signatures of selection.

Overall however I am happy with this manuscript

**Reviewer 2**

**Are you able to assess all statistics in the manuscript, including the appropriateness of statistical tests used?**

Yes, and I have assessed the statistics in my report.

**Comments to author:**

General comments:

The strength of the study by Cho et al is that they generate and utilize the genomes of many bird species (over 53 total), to investigate genetic features specific to birds of prey, and thereby identified candidate genes and their genetic changes that may give rise to predatory traits in these species. This is a first. One can tell that a lot of work also went into this study.

However, the weaknesses are: there is a lot of inconsistency in which data sets and species are used for different analyses; higher quality genome assemblies are mixed with lower quality assemblies for many analyses thereby reducing the findings to what could mainly be found with the lower quality assemblies; some analyses reveal so many changes and others very few, with statistical tools that I fear may not get rid of a lot of false positives; and the paper is not prepared well enough. For these reasons, I feel the study needs a major overhaul before it can be considered for publication. Specific concerns and suggestions for improvements are highlighted below.

Specific comments:

Abstract, line 13. Why 'probably convergent' and not more clearly convergent?

Line 15. Should mention the tissue from which the vision and circadian genes were differentially expressed in. In the main text, this seems to be blood tissue. But the authors do not explain why vision (and maybe even circadian) genes make sense as differentially regulated in blood or raptors compared to non-raptor species. The blood is not thought to be involved in vision; the brain is.

Page 4, line 5. Suggest changing to "apex predator", as ref 1 says that the common ancestor of the raptor clade of the current study was an apex predator.

Page 5, line 14. The authors need to define what is high and low quality genome assemblies here in the main text. Here they are apparently using N50 Scaffold size to define quality. In ref 1 and 6 that they cite for many of the other genomes, they defined high quality as N50 scaffold > 1Mb and low quality N50 scaffold < 1Mb (typically in the 25-100Kb range). It looks like the authors are doing the same here, but not being explicit about it. In addition, I think the authors are using genomes with 4 quality ranges: 1) N50 Scaffold > 10Mb; 2) N50 Scaffold between 1-10Mb; 3) N50 Scaffold in the Kb range; and 4) Resequencing as they call it, where they do not assemble the genome de-novo but use the same or other species as a reference to get a pseudo-reference based assembly. They should have a table where they put all the genomes they used, published and their current unpublished ones, and break them down into these 4 quality levels, with not only N50 Scaffold levels listed (Table S15), but also N50 Contig, average QV value, genome size, sequence coverage, along with the accession number. Table S6-S9 do not satisfy this need. Table S15 has the N50 scaffold numbers for only species sequenced in this study, and further has a lot of metadata that should be in a separate table.

Also what one group considers high quality is different than another group. For example, the G10K Vertebrate Genomes Project considers a genome to be high quality when the N50 contig > 1Mb, N50 Scaffold > 10Mb, and QV40, which none of the genomes of this study reach, I assume since they all use short reads https://vertebrategenomesproject.org/technology. The authors should acknowledge that the quality metric they used is more specific to this paper or based on ref 1 and 6 (Jarvis et al 2014 and Zhang et al 2014).

The authors present the re-sequencing data in a confusing manner. Re-sequencing implies that there is a reference individual with a high-quality sequence, and re-sequencing of another individual of the 'same' species was done. Instead the authors present re-sequencing of many species where there is no reference, but present it as a new de-novo assembly, using another closely related species as a reference to align to. I think this is a serious flaw of the study. When they use another species as a reference, it creates a lot of variants that are really species-specific variants and not individual variants within a species. Further, it is not a de-novo genome assembly and can't be presented as such. Findings with these shot gun reference-based assemblies are going to be clearly different than with de-novo assemblies, and should be treated separately in the paper.

Page 5, line 19. Should include in the sentence the type of assembly conducted: short read, paired end libraries of different insert sizes, and SoapDenovo or Platanus assembly algorithm.

Page 6, lines 2-6. Here is where the list of 58 genomes used (25 birds of prey + 23 non-raptors) should be cited in a table, with the quality metrics and accession numbers.

Page 6, line 11. For here and many other places in the paper, gene duplications are discussed, the authors should consider the possibility that some of these duplications are gene assembly errors of haplotypes not being properly collapsed. See Korlach et al 2017 GigaScience, which includes the hummingbird and zebra finch assemblies used in this paper. This should at least be mentioned in the discussion.

Page 6, line 23. I do not think these two cited publications are representative of what the field finds, as adaptive convergence being rare. There are many studies that have found adaptive convergent changes in the genomes of distantly related species (e.g. Ref 1 and 6, and Pfenning et al 2014 of that same issue in Science). Because the current study did not find many genes with this specific analysis, it does not mean it is rare among species. Even findings later in the current paper on dN/dS analyses find many convergent changes in raptors, contradicting this statement.

Page 7, line 3. Correct spelling to 'play'.

Page 7, line 13 and elsewhere. The authors seem to use the higher quality genomes no differently from the lower quality genomes? The analyses are going to be limited by the lower quality genomes. It seems to me that they should do some analyses with the high quality genomes only and determine if the overall results are different from when including the lower quality genomes. This will validate or invalidate the need for the high quality genomes, and give a reason why they focus on making such a statement in the beginning of the paper.

Page 7, line 21. What does citation 12 on mammals have to do with highly conserved genomic regions of the results presented in the rest of the sentence? 79 enriched functional gene categories that are thereby convergent in raptors is a lot. Are the authors sure that they do not many false positives in this analysis? Do some of these functional categories have lots of redundancies because of possibly containing the same genes? This flies in the face of the previous comment of adaptive convergence being rare.

Page 9, lines 11-14. Should cite Figure S6 for the burrowing owl result. They also state in the supplement that 'target specific amino acid substation' approach was used to find such amino acid changes, but they do not say how this was done. This terminology is similar to what Zhang et al 2014 abbreviated TAAS, in collaboration with researchers in Seoul Korea. Is this the approach the authors used?

Page 10, line 5. For the two outlier species (chicken and zebra finch) with many more gene olfactory receptor duplications, I predict they are due to the issue mentioned above of haplotypes not be properly sorted and included in the assembly as artificial gene duplications. Following the approaches in Korlach et al 2017 (half of read depth read coverage), I think the authors could check to see if this is the case.

Page 13. Overall the methods section does not use the supplementary figures and tables well enough or at all to explain to the reader what was done.

Page 13, line 4. One does not euthanize (kill) an animal to treat its wound. Do the authors mean anesthetized animal while the wound is being treated, or they mean they used tissues from animals that were euthanized because they were wounded and weren't going to survive?

Page 13, line 11. The assembly method needs to be mentioned and cited. The method appears to be that similar to Zhang et al 2014 Science used on bird species. The authors should cite the supplemental tables here for the assemblies, including the ones that mention library insert sizes.

Page 14, line 1 and before. This sentence says only one species had been used for another assembly algorithm, whereas when I read the supplement it looks like multiple species were tested on the different assembly algorithms and then the best one chosen of each. This is inconsistent between the main text and supplement. The different methods and species should be mentioned here, the criterion for choosing which assembly is better, and the appropriate supplemental figures/tables cited.

Page 14, line 7. What is homozygous variants? These two terms are contradictory. Homozygous means identical nucleotides, not variants.

Page 15, line 5. Genes with premature stop-codons were excluded. Why? This does not sound like a good idea. But on the olfactory receptors, we read later that they were not excluded. This is inconsistent between analyses.

Page 15, line 18. These divergence time numbers from Jarvis et al 2014 and TimeTree online have different dates for the some of the same divergence times. TimeTree tends to use older divergence dates than what was calculated from the whole genome scale trees in Jarvis et al. Cracraft et al 2015 Science justifies use of the dates from the Jarvis et al study. In either case, the authors need to be clear which dates they used when they were different from the two sources, and the reason why they chose one over the other.

Page 16, line 16. Why only the middle 10Kb of the 100Kb conserved block of sequence was used?

Figure 1. Not all 53 species used are here in the tree, like pigeon and hoatzin. Why not? Re-sequence implies that there is an initial assembly of that species which was not the case. I think cross-species reference based is a better term. I feel uneasy to know that there is non de-novo assemblies being grouped together in the tree with higher quality de-novo assemblies. Where do the bird drawings come from?

Figure 2. Not all birds of prey used in this study are here in the tree figure. Why not? I am finding it difficult to follow what the Venn diagram is showing. It is not cited in the main text. The only panel cited in the main text is figure 2c. This an example of poor preparation of the manuscript. For figure 2b, i suspect a lot of these duplications and gene contractions are artifacts of the assemblies. What are they gains and losses relative to who? Which reference? Have the authors checked in the raw reads of some of these species to see if they genes were really lost? See Haug-Baltzell et al (2015 Front. Neurosci. 9: Article 361, 1-16) who found that in many of these bird genomes, difficult to assemble dopamine receptor genes appeared as losses in some of the assemblies (particularly lower quality ones), when they were actually present in the raw reads. Figure 2c, what does higher z-score mean?

Figure 3. Panel a, what do the colors mean, different species? Should state this. State which method was used to construct the tree, and from one gene or many genes? Panel b, what does the x-axis value mean? Write it in the graph. Should still show the values for Piciformes and Psittaciformes.

Supplement comments:

Figure S1. What are the apparent ID numbers next to each species?

Figure S2. Put x and y axis labels in the figure; only need one label each for the entire figure. What is meaning of why oriental scops-owl has two k-mer peaks?

Table S1 and S2. Many of the citations are incorrect for the source of the genomes. For example, many of these genomes are from citations 1 and 6, but citation 2 and others are listed that did not generate these genomes (like for the Barn Owl and Bald Eagle). Warren et al 2010 Nature should be listed for the zebra finch, not ref 7. Instead of saying re-sequencing, how about saying reference-based?

Table S4. I don't understand numbers of the last 2 columns. One of them is sequencing depth (coverage) and the other?

Table S7. I thought 8 species with more complete data was mentioned in the main text, and here it says 4?

Table S13. These results prove that the authors can't used 're-sequenced' species to call variants within that species when using as a reference another species. There are 10X-20X more nucleotide variants found in the so-called re-sequenced individuals.

Table S20 and others like it. Some of these dN/dS values in bold are very close to the outgroup value, making me question are they really significantly different in raptors. In other tables like this, none of the values are bolded, making me believe the authors are being inconsistent in the way they present their results here too.

**Authors Response**

**Point-by-point responses to the reviewers’ comments:**

Reviewer #1:

Please consider the following questions:

- Are the methods appropriate to the aims of the study, are they well described, and are necessary controls included? If not, please specify what is required.

I am happy that the methods are consistent with the aims

- Are the conclusions adequately supported by the data shown? If not, please explain

Yes

- Are sufficient details provided to allow replication and comparison with related analyses that may have been performed? If not, please specify what is required.

Yes

- Does the work represent a significant advance over previously published studies?

Yes

- Is the paper of broad interest to others in the field, or of outstanding interest to a broad audience of biologists?

Broad interest to authors in the field

*>>> Thank you for the positive feedback.*

This is paper that describes the sequencing of 3 raptor genomes and identifies signatures of selection with respect to phenotype. Overall I was happy with this paper however I do find myself somewhat under-qualified to comment on the statistical aspects of the study.

For ignoramuses like me therefore I wonder if the authors could spell out a little more clearly (and in places other than the methods) why the results they obtained in terms of over-representation of gene families and specifically when they were talking about the genome, and when the transcriptome - it is not always clear.

*>>> We apologize for that. We have edited the manuscript extensively to make it clear.*

Other than that, they may wish to comment on how close their genomes are to chromosome level assemblies and the role of EBRs and HSBs in establishing signatures of selection.

Overall however I am happy with this manuscript

*>>> Thank you for the constructive suggestions.*

*As the reviewer suggested, genome structural comparisons at chromosome level including evolutionary breakpoint regions (EBRs) and homologous synteny blocks (HSBs) could provide insights into different evolutionary histories in avian species (Zhang G, et al. Science 2014; Larkin DM, et al. Genome Res. 2009; Farré M et al. Genome Biol. Evol. 2016). To accommodate the reviewer’s suggestion, we have tried to identify EBRs and HSBs, but the SyntenyTracker software for the analyses was not available anymore.*

*So far, very few avian genomes, such as chicken, zebra finch, and turkey, have been successfully assembled at chromosome level. However, genome assemblies based on NGS short-read sequencing methods like our four raptor genomes usually possess a relatively high-level of error rate in the genomic structure aspect, making difficult to compare genomic structures accurately; actually, Farré M et al. identified EBRs within scaffolds, but the result showed very high levels of false positives (~22%) and false negatives (33-45%). Therefore, we have focused on the evolutionary selection analysis at gene sequence level for the raptor/nocturnal bird genomes in this manuscript.*

*Having expressed the above, the Avian Phylogenomics Project (leaded by Tom Gilbert, Erich Jarvis, and Guojie Zhang) has updated/generated new chromosome level genomes from at least one species per avian order (https://vertebrategenomesproject.org/). They have constructed avian genomes with PacBio long reads for initial assembly, BioNano optical maps for intermediate range scaffolding, and Hi-C for long-range scaffolding, aiming to generate near error-free chromosome-level reference genome assembly. We think that the avian genomes by this project could us encourage us to analyze avian genome architecture.*

Reviewer #2:

General comments:

The strength of the study by Cho et al is that they generate and utilize the genomes of many bird species (over 53 total), to investigate genetic features specific to birds of prey, and thereby identified candidate genes and their genetic changes that may give rise to predatory traits in these species. This is a first. One can tell that a lot of work also went into this study.

However, the weaknesses are:

there is a lot of inconsistency in which data sets and species are used for different analyses; higher quality genome assemblies are mixed with lower quality assemblies for many analyses thereby reducing the findings to what could mainly be found with the lower quality assemblies;

*>>> Thank you for the feedback.*

*In the initial manuscript, we used high-quality assemblies only for the comparisons among three raptor orders (except the GC3 bias analysis that was separately calculated for each species genome), and used both high- and low-quality assemblies when we analyzed the nocturnality. This is because there were only two nocturnal bird groups (true owls and kiwi) when considering only high-quality genomes. Since several avian clades have adapted to a nocturnal lifestyle independently (Le Duc D et al. Bioessays 2015), we thought that comparisons with more independent nocturnal bird groups have a benefit by reducing species/order specific and keeping common evolutionary signatures.*

*To accommodate the reviewer’s criticism, we have updated the gene expansion/contraction analysis using high-quality assemblies only (excluding low-quality barn owl and chuck-will’s-widow), of which analysis is the mostly affected by the assembly quality. Also, we added a new filtering analysis based on sequence depth information to remove false-positives derived from the assembly quality (see Figure S9 and Methods). As a result, we could identify some sensory perception (such as smell) associated gene families were expanded in the nocturnal birds (Supplementary Table S30), which may be related to the functional trade-offs in the sensory systems. Accordingly, we have revised/added related texts in the main text (see below point-by-point responses).*

*On the other hand, we kept other gene sequence-level comparison analyses using both high- and low-quality genomes for the nocturnality, as low quality genomes could also play an important role by reducing species/order specific and keeping common evolutionary signatures.*

some analyses reveal so many changes and others very few, with statistical tools that I fear may not get rid of a lot of false positives;

*>>> We disagree with this point. Each analysis has been conducted using widely used methods with similar criteria, and evolutionary signature can be found differently depending on the analysis.*

*For the false-positive issue on the gene expansion/contraction analysis, we added a filtering analysis based on read depth information to get rid of possible false-positives derived from assembly process as described above. and the paper is not prepared well enough.*

For these reasons, I feel the study needs a major overhaul before it can be considered for publication. Specific concerns and suggestions for improvements are highlighted below.

*>>> We have revised the overall manuscript to clarify our findings. (please see below point-by-point responses.)*

Specific comments:

Abstract, line 13. Why 'probably convergent' and not more clearly convergent?

*>>> We changed ‘probably convergent’ to ‘convergent’.*

Line 15. Should mention the tissue from which the vision and circadian genes were differentially expressed in. In the main text, this seems to be blood tissue. But the authors do not explain why vision (and maybe even circadian) genes make sense as differentially regulated in blood or raptors compared to non-raptor species. The blood is not thought to be involved in vision; the brain is.

*>>> Good point. We agree with the reviewer’s criticism on blood tissue issue for vision gene expression analysis.*

*To analyze expression profiles of vision genes, it would be best to analyze retinal tissue transcriptome as Wu Y et al. (Sci. Rep. 2016) did; however, Wu Y et al. investigated selection signature only at DNA sequence level, and did not compare expression profiles between nocturnal and diurnal raptors. Unfortunately, it is extremely difficult to acquire fresh retinal tissues required for these analyses, partly because the raptors are wild and also because they are protected by law in Korea. Given these limitations, we would like to compare gene expression profiles between nocturnal and diurnal birds using blood transcriptome to hint adaptive expression changes of owls. Actually, it has been reported that vision-associated genes that were mainly expressed in retina and pineal gland were also expressed in blood and other organ (GeneCard database).*

*For the circadian rhythm genes, it has been reported that several genes and compounds expressed in other tissues could affect circadian rhythm by regulating the expression level of circadian clock associated genes (Govindarajan K et al. PLoS One 2016; Zhang F et al. Chronobiol. Int. 2018) and that circadian rhythm associated genes were differentially expressed in blood at human circadian phase (Lech K et al, J. Biol. Rhythms, 2016; Laing EE et al, Elife, 2017).*

*To accommodate the reviewer’s point, we first added tissue information in the abstract as follows:*

*“Additionally, we found that a suite of genes associated with vision and circadian rhythm were differentially expressed in blood tissue between nocturnal and diurnal raptors, possibly indicating adaptive expression change during the transition to nocturnality.”*

*Also, we added a limitation of our gene expression analysis for vision genes in the Results and discussion section as follows:*

*“However, the comparison of gene expressions based on blood transcriptome could not represent gene expression profiles of vision system, and therefore, further studies are needed to confirm our results (e.g., analyzing expression profiles of retinal tissue transcriptome).”*

Page 4, line 5. Suggest changing to "apex predator", as ref 1 says that the common ancestor of the raptor clade of the current study was an apex predator.

*>>> We changed.*

Page 5, line 14. The authors need to define what is high and low quality genome assemblies here in the main text. Here they are apparently using N50 Scaffold size to define quality. In ref 1 and 6 that they cite for many of the other genomes, they defined high quality as N50 scaffold > 1Mb and low quality N50 scaffold < 1Mb (typically in the 25-100Kb range). It looks like the authors are doing the same here, but not being explicit about it. In addition, I think the authors are using genomes with 4 quality ranges: 1) N50 Scaffold > 10Mb; 2) N50 Scaffold between 1-10Mb; 3) N50 Scaffold in the Kb range; and 4) Resequencing as they call it, where they do not assemble the genome de-novo but use the same or other species as a reference to get a pseudo-reference based assembly.

*>>> Yes, we defined high quality as scaffold N50 length of >1Mb and low quality as scaffold N50 length of <1Mb, similar to that Guojie Z et al. (Science 2014) did; more precisely, Guojie Z et al. used a sequence depth criterion (high [>50×] coverage and low [~30×] coverage) instead of scaffold N50 length. Therefore, we added an explanation for the high/low quality criterion as follows:*

*“By assessing assembly statistics, transcripts mapping results, and single-copy orthologs mapping results (Additional file 1: Tables S7, S8, and S10), we obtained the final reference genomes for the four raptor species at a high-quality, resulting in scaffold N50 sizes from 7.49 to 29.92 Mb; we defined as high quality genome if scaffold N50 length of > 1 Mb and as low quality genome if scaffold N50 length of < 1 Mb, similar to the previous studies [1, 6] (Additional file 1: Table S11).”*

*For the whole genome sequenced but not de novo assembled genomes (mis-called as ‘resequencing’ in the initial manuscript), we separately defined them as ‘whole genome sequence (WGS)’ as follows:*

*“The whole genome sequences of the twelve raptors and four non-raptor birds were not assembled, but aligned to the reference genomes of the closely related species for comparison purposes to remove possible bias derived from a small number of raptor/nocturnal species genomes; the whole genome sequenced but not assembled genomes were hereinafter referred to as whole genome sequence (WGS).”*

They should have a table where they put all the genomes they used, published and their current unpublished ones, and break them down into these 4 quality levels, with not only N50 Scaffold levels listed (Table S15), but also N50 Contig, average QV value, genome size, sequence coverage, along with the accession number. Table S6-S9 do not satisfy this need. Table S15 has the N50 scaffold numbers for only species sequenced in this study, and further has a lot of metadata that should be in a separate table.

*>>> We added a table that put all the genomes we used with additional assembly metrics (Additional file 1: Table S11). Also, we split metadata for the genome assembled species into Table S16.*

Also what one group considers high quality is different than another group. For example, the G10K Vertebrate Genomes Project considers a genome to be high quality when the N50 contig > 1Mb, N50 Scaffold > 10Mb, and QV40, which none of the genomes of this study reach, I assume since they all use short reads https://vertebrategenomesproject.org/technology. The authors should acknowledge that the quality metric they used is more specific to this paper or based on ref 1 and 6 (Jarvis et al 2014 and Zhang et al 2014).

*>>> Yes, the Vertebrate Genomes Project (VGP) by Genome 10K consortium used much higher criterion for the high quality genome, as the VGP has built animal genome upto chromosome levels using long read sequencing and whole genome mapping technologies. We added the more detailed quality metrics more detail in Table S11.*

The authors present the re-sequencing data in a confusing manner. Re-sequencing implies that there is a reference individual with a high-quality sequence, and re-sequencing of another individual of the 'same' species was done. Instead the authors present re-sequencing of many species where there is no reference, but present it as a new de-novo assembly, using another closely related species as a reference to align to.

*>>> Yes, the reviewer is right. We revised re-sequencing to whole genome sequence (WGS).*

I think this is a serious flaw of the study. When they use another species as a reference, it creates a lot of variants that are really species-specific variants and not individual variants within a species.

*>>> Good point. Aligning to the different species’ reference genome could identify many species-specific variants, not individual variants. For example, different copy numbered regions could generate false heterozygous variants.*

*However, we would like to emphasize that our analyses using WGS data focused to identify commonly shared genetic signatures among raptors or nocturnal birds by restricting to the well-aligned/conserved genomic regions and that we did not focus on any species- and individual-specific variants/signatures. Actually, we used WGS data in two analyses (target specific amino acid substitution and highly conserved regions [HCRs]) in order to remove species-specific amino acid sequences and possible bias derived from comparisons with a small number of genomes in the HCRs analysis.*

*Having expressed above, we have successfully analyzed many animal genomes by comparing whole genome sequences without reference genome to the reference genome of closely relative species (Tiger genome, Cho YS et al. Nat. Commun. 2013; Minke whale genome, Yim HS et al. Nat. Genet. 2014; Leopard genome, Kim S et al. Genome Biol. and so on).*

Further, it is not a de-novo genome assembly and can't be presented as such. Findings with these shot gun reference-based assemblies are going to be clearly different than with de-novo assemblies, and should be treated separately in the paper.

*>>> We separately called it as ‘WGS’.*

Page 5, line 19. Should include in the sentence the type of assembly conducted: short read, paired end libraries of different insert sizes, and SoapDenovo or Platanus assembly algorithm.

*>>> This is related to above points, and the WGS data were not de novo assembled. This mis-understanding is due to our unclear statements and we have fixed this as follows:*

*“The whole genome sequences of the twelve raptors and four non-raptor birds were not assembled, but aligned to the reference genomes of the closely related species for comparison purposes to remove possible bias derived from a small number of raptor/nocturnal species genomes; the whole genome sequenced but not assembled genomes were hereinafter referred to as whole genome sequence (WGS).”*

*We also added texts for sequencing and assembly for four de novo assembled genomes more detail as follows (see the Raptor genome sequencing and assembly part of the Results and discussion section):*

*“We applied whole-genome shotgun sequencing and de novo assembly strategies [6, 10-12] to build reference genomes of the four raptor species (Eurasian eagle-owl, oriental scops-owl, eastern buzzard, and common kestrel). The extracted DNA samples from wild individuals were sequenced using Illumina HiSeq platforms at high coverage (>185×) using various insert sizes of short-insert (170bp, 500bp, and 700bp for the two owls and eastern buzzard, and 350bp and 550bp for the common kestrel) and long-mate pair libraries (2 Kb, 5 Kb, 10 Kb, and 15 Kb; Additional file 1: Tables S4 and S5). The four raptor genomes showed relatively higher levels of genomic diversity compared to the previously assembled genomes of eagles and falcons (Additional file 1: Figures S2 and S3). Therefore, we tried to assemble reference genomes of the four raptor species using both SOAPdenove2 [10] and Platanus [11] software in various conditions (Additional file 1: Tables S6-S8). Protein-coding genes (~16,000 to 18,000 genes) for these assemblies were predicted by combining de novo and homologous gene prediction methods with whole blood transcriptome data (Additional file 1: Table S9). By assessing assembly statistics, transcripts mapping results, and single-copy orthologs mapping results (Additional file 1: Tables S7, S8, and S10), we obtained the final reference genomes for the four raptor species at a high-quality, resulting in scaffold N50 sizes from 7.49 to 29.92 Mb; we defined as high quality genome if scaffold N50 length of > 1 Mb and as low quality genome if scaffold N50 length of < 1 Mb, similar to the previous studies [1, 6] (Additional file 1: Table S11).”*

Page 6, lines 2-6. Here is where the list of 48 genomes used (25 birds of prey + 23 non-raptors) should be cited in a table, with the quality metrics and accession numbers.

*>>> Thank you. We added a table and cited it (Table S11).*

*In order to make it easier to understand, we also added texts for orthologous gene cluster using only assembled genomes as follows (see the first paragraph of ‘Evolutionary analysis of raptors compared to non-raptor birds’ of the Results and discussion section):*

*“First, gene family clusters were constructed using a total of 25 assembled avian genomes (both 23 high and two low quality genomes; Additional file 1: Tables S11 and S16). Of the 29,115 orthologous gene families found in the 25 avian genomes, 12,662 were found in the all raptor genomes (Fig. 2a; Additional file 1: Figure S4). Based on the comparison of orthologous gene families among the only 23 high quality avian genomes, 132 expanded and 556 contracted, 69 expanded and 1,283 contracted, and 26 expanded and 551 contracted gene families were found in the common ancestors of Strigiformes, Accipitriformes, and Falconiformes, respectively, compared with the common ancestors of each raptor order and its sister group (Fig. 2b).”*

Page 6, line 11. For here and many other places in the paper, gene duplications are discussed, the authors should consider the possibility that some of these duplications are gene assembly errors of haplotypes not being properly collapsed. See Korlach et al 2017 GigaScience, which includes the hummingbird and zebra finch assemblies used in this paper. This should at least be mentioned in the discussion.

*>>> Very good point. We agree with the reviewer’s point and have updated our analyses.*

*To remove false-positives derived from assembly errors, we calculated the average depth coverage of genes in raptors and nocturnal birds (see Figure S9 and Methods). As a result, we found that some of the genes showed low depth coverage (at less than half of average depth) compared to average mapping depth coverage, indicating that misassembled genes/regions were existed.*

*Therefore, we updated the gene expansion and contraction analysis by filtering out the genes having abnormal depth of coverage (less than half of average depth coverage or more than twice of average depth coverage). Additionally, we also exclude low-quality nocturnal genomes in this analysis as described above.*

*To accommodate the reviewer’s criticisms, we also added texts this issue in the Conclusions section as follows:*

*“At the same time, it is important to note that genome assembly based on short read sequencing methods could possess incomplete genomic regions, thus causing an erroneous result in comparative evolutionary analyses [36, 37]. Therefore, the candidate genes identified in this study need to be further confirmed with additional genomic data, and functional studies of candidate genes will be needed to understand the molecular mechanisms of adaptation.”*

Page 6, line 23. I do not think these two cited publications are representative of what the field finds, as adaptive convergence being rare. There are many studies that have found adaptive convergent changes in the genomes of distantly related species (e.g. Ref 1 and 6, and Pfenning et al 2014 of that same issue in Science). Because the current study did not find many genes with this specific analysis, it does not mean it is rare among species. Even findings later in the current paper on dN/dS analyses find many convergent changes in raptors, contradicting this statement.

*>>> We agree with the reviewer’s criticism, and remove it.*

Page 7, line 3. Correct spelling to 'play'.

*>>> Thanks, we corrected.*

Page 7, line 13 and elsewhere. The authors seem to use the higher quality genomes no differently from the lower quality genomes? The analyses are going to be limited by the lower quality genomes. It seems to me that they should do some analyses with the high quality genomes only and determine if the overall results are different from when including the lower quality genomes. This will validate or invalidate the need for the high quality genomes, and give a reason why they focus on making such a statement in the beginning of the paper.

*>>> This is related to above points.*

*As we described above, we updated the gene expansion/contraction analysis using only high quality genomes. On the other hand, in order to remove false derived from comparisons with a small number of genomes, we used both high and low quality assembled genomes for positive selection, specific amino acid sequence, and GC3 bias analyses that are conducted on well matched/conserved regions.*

*Since several avian clades have adapted to a nocturnal lifestyle independently (Le Duc D et al. Bioessays 2015), we thought that comparisons with more independent nocturnal bird groups have a benefit by reducing species/order specific and keeping common evolutionary signatures. As the reviewer criticized, lower quality assemblies could make reduce the findings, but we think this is a matter of choice for a trade-off between sensitivity and specificity in usual comparative genome analyses.*

Page 7, line 21. What does citation 12 on mammals have to do with highly conserved genomic regions of the results presented in the rest of the sentence? 79 enriched functional gene categories that are thereby convergent in raptorsis a lot. Are the authors sure that they do not many false positives in this analysis? Do some of these functional categories have lots of redundancies because of possibly containing the same genes? This flies in the face of the previous comment of adaptive convergence being rare.

*>>> We apologize for the lack of clarity on our part. We used the same method of ref 12 for highly conserved genomic regions (HCRs) analysis. We removed the citation 12 in that sentence (the same citation was in the Methods).*

*We applied a proper statistical test and used very strict criteria: 1) q-value <0.0001; 2) only middle 10Kb out of 100 Kb of each sliding window to remove false positives. Also, we calculated HCRs in Passeriformes as a control group and compared them with those of the three raptor orders in order to identify raptor specific HCRs.*

*We also removed “adaptive convergence being rare” as described above.*

Page 9, lines 11-14. Should cite Figure S6 for the burrowing owl result. They also state in the supplement that 'target specific amino acid substation' approach was used to find such amino acid changes, but they do not say how this was done. This terminology is similar to what Zhang et al 2014 abbreviated TAAS, in collaboration with researchers in Seoul Korea. Is this the approach the authors used?

*>>> We cited Figure S6 here. Yes, our method is almost similar with that Zhang et al did (TAAS), but our analysis includes an additional filtering step using WGS data and NCBI nr database to remove false positives. The target species-specific amino acid substitution approach was already described in Methods (we also added the citation) as follows:*

*“We identified target species-specific amino acid sequences [6]. To filter out biases derived from individual-specific variants, we used all of the raptor WGS data by mapping to the Eurasian eagle-owl genome for Strigiformes, the eastern buzzard genome for Accipitriformes, and the common kestrel genome for Falconiformes. The mapping was conducted using BWA-MEM, and consensus sequences were generated using SAMtools with the default options, except the “-d 5” option. When we identified the specific amino acid sequences, protein sequences of other birds from the NCBI database were also compared. We also checked multiple-sequence alignments manually to remove artifacts.”*

Page 10, line 5. For the two outlier species (chicken and zebra finch) with many more gene olfactory receptor duplications, I predict they are due to the issue mentioned above of haplotypes not be properly sorted and included in the assembly as artificial gene duplications. Following the approaches in Korlach et al 2017 (half of read depth read coverage), I think the authors could check to see if this is the case.

*>>> Khan I et al. (Mol. Biol. Evol. 2015) analyzed olfactory receptors (ORs) in avian genomes. They already analyzed and discussed on why chicken and zebra finch possess many ORs in their genomes. They found that there was no correlation between the normalized mapping depth of OR genes and sequencing depth, guessing that chicken and zebra finch genomes recovered many OR genes as their genomes were assembled using traditional longer sequencing (Sanger) method. Therefore, we cited this study.*

Page 13. Overall the methods section does not use the supplementary figures and tables well enough or at all to explain to the reader what was done.

*>>> We updated the Methods section by using Supplementary data.*

Page 13, line 4. One does not euthanize (kill) an animal to treat its wound. Do the authors mean anesthetized animal while the wound is being treated, or they mean they used tissues from animals that were euthanized because they were wounded and weren't going to survive?

*>>> Yes, the reviewer is right. We revised as follows:*

*“All blood samples used for genome and transcriptome sequencing were collected from individuals being euthanized due to poor survival during wound treatment of rescued animals…”*

Page 13, line 11. The assembly method needs to be mentioned and cited. The method appears to be that similar to Zhang et al 2014 Science used on bird species. The authors should cite the supplemental tables here for the assemblies, including the ones that mention library insert sizes.

*>>> The assembly methods were already described in the ‘Genome assembly and annotation’ part in the Methods section. We now added texts for assembly method more detail in the main text as described above. Also, we cited supplementary figures/tables in the Methods.*

Page 14, line 1 and before. This sentence says only one species had been used for another assembly algorithm, whereas when I read the supplement it looks like multiple species were tested on the different assembly algorithms and then the best one chosen of each. This is inconsistent between the main text and supplement. The different methods and species should be mentioned here, the criterion for choosing which assembly is better, and the appropriate supplemental figures/tables cited.

*>>> We apologize for the lack of clarity on our part. As the reviewer said, we have assembled four raptor genomes using both two assemblers (SOAPdenovo2 and Platanus). We revised the texts and cited supplementary data to make it clear as follows:*

*“As the four raptor genomes showed relatively higher levels of genomic diversity (Additional file 1: Figures S2 and S3), we assembled reference genomes of the four raptor species using both SOAPdenove2 [10] and Platanus [11] software; the Platanus assembler is more efficient for highly heterozygous genomes [11]. When performing the SOAPdenovo2 assembler, we applied various K-mer values (33, 43, 53, and 63) to obtain fragments with long contiguity. To reduce the number of gaps in the scaffolds, we closed the gaps using the short-insert library reads in two iterations. To correct base-pair level errors, we performed two iterations of aligning the short-insert library reads to the gap-closed scaffolds using BWA-MEM [38] and calling variants using SAMtools [39]. In this process, homozygous variants were assumed as erroneous sequences from the assembly process, and thus substituted for the correction purpose (Additional file 1: Table S7).”*

Page 14, line 7. What is homozygous variants? These two terms are contradictory. Homozygous means identical nucleotides, not variants.

*>>> Homozygous variants mean two copies of nucleotides were the same each other but different from the nucleotide of the reference.*

Page 15, line 5. Genes with premature stop-codons were excluded. Why? This does not sound like a good idea. But on the olfactory receptors, we read later that they were not excluded. This is inconsistent between analyses.

*>>> Our major analyses focused on intact protein-coding genes. Therefore, we excluded genes with premature stop-codons in the gene annotation process. For the olfactory receptors (ORs), we independently predicted OR genes, to compare the olfactory receptors across avian clades more precisely by searching genomes with more ORs sequence queries (commonly used methods to identify ORs). The pseudogenes were results of the independent OR annotation process, and we used only intact OR genes in the comparison and Figure 3 as described in the legend and Methods. To make it clear, we revised related texts.*

Page 15, line 18. These divergence time numbers from Jarvis et al 2014 and TimeTree online have different dates for the some of the same divergence times. TimeTree tends to use older divergence dates than what was calculated from the whole genome scale trees in Jarvis et al. Cracraft et al 2015 Science justifies use of the dates from the Jarvis et al study. In either case, the authors need to be clear which dates they used when they were different from the two sources, and the reason why they chose one over the other.

*>>> We used divergence times from TimeTree database as a guide date to calculate divergence times among representative avian species (Fig. 2b). When we estimated divergence times among raptors (Fig. 1), we found that divergence times of TimeTree and Jarvis et al Science 2014 were quite different because of the different phylogenetic topology. Therefore, we estimated divergence times among raptors by constraining to divergence times of Jarvis et al as the minimum and divergence times of TimeTree as the maximum. Accordingly, we updated the related methods to make it clear as follows:*

*“To estimate divergence times among the birds of prey (Fig. 1), the date of the node between downy woodpecker and Eurasian eagle-owl constrained to 61-78 MYA and common kestrel and budgerigar was constrained to 60-80 MYA according to the divergence times from the previous studies [1, 6] and TimeTree; as the divergence times and phylogenetic topologies of the previous studies [1, 6] and TimeTree were quite different, we used the divergence times from the previous studies as the minimum and the divergence times from the TimeTree database as the maximum constraints.”*

Page 16, line 16. Why only the middle 10Kb of the 100Kb conserved block of sequence was used?

*>>> Both ends of scaffolds have usually incorrect sequences and many gaps, causing low mapping depths and inaccurate variants. To remove those regions, we used middle of 10Kb out of the 100Kb window. Also, the middle 10Kb of the conserved block was concatenated with the next middle 10Kb if the next block were also highly conserved. We added texts for why we used only the middle 10Kb in the Methods as follows:*

*“As both ends of scaffolds have usually incorrect sequences and many gaps, the middle 10 Kb of each significantly different window were only considered as HCRs (Additional file 1: Table S20).”*

Figure 1. Not all 53 species used are here in the tree, like pigeon and hoatzin. Why not? Re-sequence implies that there is an initial assembly of that species which was not the case. I think cross-species reference based is a better term. I feel uneasy to know that there is non de-novo assemblies being grouped together in the tree with higher quality de-novo assemblies. Where do the bird drawings come from?

*>>> Fig. 1 was drawn to emphasize that our raptor genomes extend the genomic coverage of the raptor species. Therefore, we drew Figure 1 by focusing on raptor branches with their genomic data types and phylogenetic relationships (the phylogenetic tree for all the assembled avian genomes was shown in the Fig. 2b).*

*We revised resequencing to WGS as described above. The bird illustrations were drawn by a designer Ara Jo in our group. We acknowledged her in the Acknowledgements section.*

Figure 2. Not all birds of prey used in this study are here in the tree figure. Why not? I am finding it difficult to follow what the Venn diagram is showing. It is not cited in the main text. The only panel cited in the main text is figure 2c. This an example of poor preparation of the manuscript. For figure 2b, i suspect a lot of these duplications and gene contractions are artifacts of the assemblies. What are they gains and losses relative to who? Which reference? Have the authors checked in the raw reads of some of these species to see if they genes were really lost? See Haug-Baltzell et al (2015 Front. Neurosci. 9: Article 361, 1-16) who found that in many of these bird genomes, difficult to assemble dopamine receptor genes appeared as losses in some of the assemblies (particularly lower quality ones), when they were actually present in the raw reads. Figure 2c, what does higher z-score mean?

*>>> The tree in the Fig. 2b was for assembled genomes (both high and low quality) to analyze gene expansion/contraction. Therefore, WGS birds of prey were not included.*

*For the gene gain/loss analysis, we updated the analysis using high-quality genomes only and added a filtering step based on the mapping depth coverage as described above. Accordingly, we have updated Fig. 2b using only high-quality genomes.*

*Fig. 2a represents the orthologous gene cluster result. We added texts for the orthologous gene cluster result and cited this in the main text as follows:*

*“First, gene family clusters were constructed using a total of 25 assembled avian genomes (both 23 high and two low quality genomes; Additional file 1: Tables S11 and S16). Of the 29,115 orthologous gene families found in the 25 avian genomes, 12,662 were found in the all raptor genomes (Fig. 2a; Additional file 1: Figure S4). Based on the comparison of orthologous gene families among the only 23 high quality avian genomes, 132 expanded and 556 contracted, 69 expanded and 1,283 contracted, and 26 expanded and 551 contracted gene families were found in the common ancestors of Strigiformes, Accipitriformes, and Falconiformes, respectively, compared with the common ancestors of each raptor order and its sister group (Fig. 2b).”*

*The higher Z-score in the Figure 2c means the higher GC3-bias. It has been suggested that genes with higher GC3-bias are more adaptable to external stresses (Tatarinova TV et al, J. Clin. Inverst. 1999), and therefore, we speculated that enriched function by GC3-biased genes may be related to evolution and adaptation of the raptors/nocturnal birds.*

Figure 3. Panel a, what do the colors mean, different species? Should state this. State which method was used to construct the tree, and from one gene or many genes? Panel b, what does the x-axis value mean? Write it in the graph. Should still show the values for Piciformes and Psittaciformes.

*>>> Yes, colors in Fig. 3a mean different species. We now stated this in the legend.*

*The tree was constructed using ClustalW2 when classifying the OR clade-assign steps as described in the Methods section. We now added these in the legend as follows:*

*“The phylogeny of the α and γ olfactory receptor (OR) genes identified in 25 avian genomes. The phylogeny information was constructed for intact OR genes only using ClustalW2 software. Colors in the label mean different avian species.”*

*Also, we added the x-axis in the Fig. 3b. For the values for Piciformes and Psittaciformes regarding ORs, we already explained why they could not be calculated in the legend (“The diversity of α ORs in Piciformes and γ-c ORs in Psittaciformes were not calculated as the number of identified OR genes were smaller than two.”).*

Supplement comments:

Figure S1. What are the apparent ID numbers next to each species?

*>>> Id numbers are NCBI accession ID. We added this in the legend.*

Figure S2. Put x and y axis labels in the figure; only need one label each for the entire figure. What is meaning of why oriental scops-owl has two k-mer peaks?

*>>> We added labels. The double peaks in k-mer analysis of oriental scops-owl indicate that the oriental scops-owl has a high degree of heterozygosity between the genomes of the two haploids (see Liu B et al. arXiv:1308.2012).*

Table S1 and S2. Many of the citations are incorrect for the source of the genomes. For example, many of these genomes are from citations 1 and 6, but citation 2 and others are listed that did not generate these genomes (like for the Barn Owl and Bald Eagle). Warren et al 2010 Nature should be listed for the zebra finch, not ref 7. Instead of saying re-sequencing, how about saying reference-based?

*>>> The citations in supplementary files were separated from those of the main text (see Supplementary References in Additional file 1). We updated a citation form in supplementary files as “S1, S2, S3, ...”.*

Table S4. I don't understand numbers of the last 2 columns. One of them is sequencing depth (coverage) and the other?

*>>> We apologize for the lack of clarity. Both the last 2 columns are sequencing depth (coverage), but we presented left- and right- reads separately. We updated Tables S4-S5 by integrating both end reads.*

Table S7. I thought 8 species with more complete data was mentioned in the main text, and here it says 4?

*>>> We apologize for the lack of clarity. As we described, we assembled four raptor genomes, and the other WGS genomes were not assembled.*

Table S13. These results prove that the authors can't used 're-sequenced' species to call variants within that species when using as a reference another species. There are 10X-20X more nucleotide variants found in the so-called re-sequenced individuals.

*>>> As described above, we revised re-sequenced/resequencing to WGS.*

Table S20 and others like it. Some of these dN/dS values in bold are very close to the outgroup value, making me question are they really significantly different in raptors. In other tables like this, none of the values are bolded, making me believe the authors are being inconsistent in the way they present their results here too.

*>>> Yes, the referee is right. In the raptor beak analysis, we did not apply a statistical test, as no beak associated genes were significant. Therefore, we removed this result.*

*For the other dN/dN analyses, we applied a statistical criterion and used significant results only.*

**Second round of review**

**Reviewer 1**

Not invited for re-review.

**Reviewer 2**

**The authors were very responsive to my comments, performed additional analyses, cleaned up** many analyses, improved the writing, and all as a result, the paper is much improved. I only have a few minor remaining concerns:

The authors now further guarantee that there are not looking at false duplications by filter out genes that have less than half the depth of coverage of raw reads against the genomes. This is a good idea. But they should also note that sex chromosomes will have half the coverage when the heterogametic sex (female birds). Is sequenced. So they may have eliminated some of the sex chromosome genes from their analyses, but not the autosome genes.

Page 6, line 7. I recommend revising to: "The whole genome sequences (WGS) of the 12 additional raptors…."

Page 12, last sentence. Change "could" to "may". Also include visual brain regions, besides the retina, for future transcriptome measures.

Figure S2, mention that individuals with 2 peaks in the k-mer plot represent greater heterozygosity in them.

**Authors Response**

**Point-by-point responses to the reviewers’ comments:**

Reviewer #2: The authors were very responsive to my comments, performed additional analyses, cleaned up many analyses, improved the writing, and all as a result, the paper is much improved. I only have a few minor remaining concerns:

The authors now further guarantee that there are not looking at false duplications by filter out genes that have less than half the depth of coverage of raw reads against the genomes. This is a good idea. But they should also note that sex chromosomes will have half the coverage when the heterogametic sex (female birds). Is sequenced. So they may have eliminated some of the sex chromosome genes from their analyses, but not the autosome genes.

*Good point. We updated the filtering process in gene duplication analysis by using different depth coverage criteria on genes in autosomal scaffolds (1/2 < average depth coverage < 2) and genes in sex chromosomal scaffolds (1/4 < average depth coverage < 2). Accordingly, we also updated related results and texts; there was no difference in the major findings/results.*

Page 6, line 7. I recommend revising to: "The whole genome sequences (WGS) of the 12 additional raptors…."

*We revised as suggested.*

Page 12, last sentence. Change "could" to "may". Also include visual brain regions, besides the retina, for future transcriptome measures.

*We revised as suggested.*

Figure S2, mention that individuals with 2 peaks in the k-mer plot represent greater heterozygosity in them.

*We added.*
